# Site-selective carbonylation of arenes *via* C(sp^2^)–H thianthrenation: direct access to 1,2-diarylethanones†‡

**DOI:** 10.1039/d3sc02402d

**Published:** 2023-06-16

**Authors:** Jiajun Zhang, Le-Cheng Wang, Zhi-Peng Bao, Xiao-Feng Wu

**Affiliations:** a Dalian National Laboratory for Clean Energy, Dalian Institute of Chemical Physics, Chinese Academy of Sciences 116023 Dalian Liaoning China xwu2020@dicp.ac.cn; b Leibniz-Institut für Katalyse e.V. Albert-Einstein-Straße 29a 18059 Rostock Germany Xiao-Feng.Wu@catalysis.de

## Abstract

Herein, a new reaction for the site-selective carbonylation of arenes *via* C(sp^2^)–H thianthrenation under mild conditions has been developed. With low loadings of palladium catalysts, various desired 1,2-diarylethanones are produced in good yields. This strategy also enables the late-stage modification of complex molecules, which was previously challenging with similar carbonylative Negishi-type reactions.

## Abstract 2

Herein, a new reaction for the site-selective carbonylation of arenes *via* C(sp^2^)–H thianthrenation under mild conditions has been developed.

## Graphical abstract

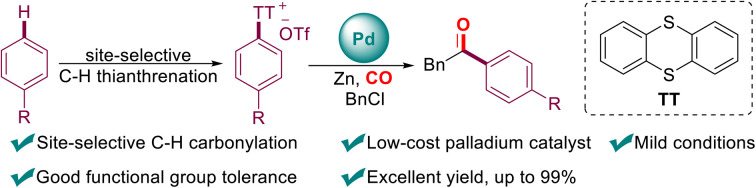

## Introduction

**Scheme 1:**
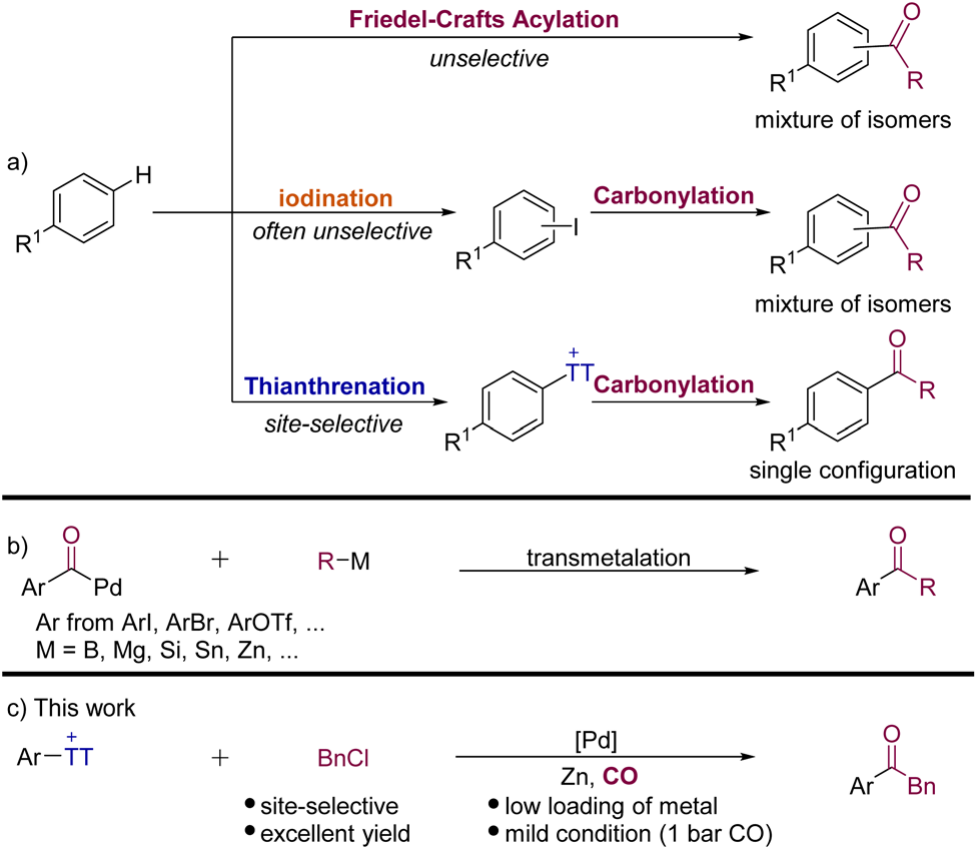
(a) Conceptual representation of various strategies for undirected C–H acylation of arenes; (b) palladium-catalyzed carbonylative coupling reactions; (c) this work: Palladium-catalyzed carbonylative Negishi-type coupling of aryl thianthrenium salts with benzyl chlorides.

The activation and direct functionalization of inert bonds have always been a hot topic pursued by chemists,^1^ especially the C–H bond activation of aromatic hydrocarbons which can directly modify natural products and also macromolecules at the late stage. Among the known transformations, Friedel–Crafts acylation is a classic reaction for the C–H bond functionalization of arenes (Scheme 1a).^2^ However, its related low regioselectivity and over-acylation often cause synthetic problems. The halogenation of arenes to give aryl halides has been proven to be effective,^3^ but the synthesis of complex aromatic halides often involves site-selectivity and tolerance issues (Scheme 1a).^4^ On the other hand, C(sp^2^)–H thianthrenation has been recognized as an important means for the direct functionalization of arenes. Its mild reaction conditions and high regioselectivity are considered as the greatest advantages.^5^ Hence, aryl thianthrenium salts have been successfully used to construct C–C, C–B, C–N, and C–Si bonds, *etc.*^5,6^ More recently, the advantage of aryl thianthrenium salts has been applied by Cornella, Ritter and their co-workers in nickel-catalyzed synthesis of aryl halides from aryl thianthrenium salts.^7^ This protocol can produce aryl halides that would not readily be available by direct halogenation of arenes.

Transition-metal-catalyzed carbonylative cross-coupling reactions that form C–C bonds have become a powerful platform to synthesize ketones and their derivatives.^8^ In recent years, many palladium-catalyzed carbonylative coupling reactions for constructing C–C bonds have been established. According to different carbon nucleophilic reagents, the reactions can be divided into Suzuki (B),^9^ Kumada (Mg),^10^ Hiyama (Si),^11^ Stille (Sn),^12^ and Negishi (Zn) reactions.^8,13^ These reactions have been quite well developed, which provide convenient pathways for constructing ketones.

Although the carbonylative Negishi-type reaction is among the most efficient methods to obtain ketones from aryl halides, one of the long-standing challenges is competitive direct coupling reaction and also the availability and stability of substrates limited the applications of this carbonylative cross-coupling reaction. In order to overcome these limitations, alternative methods have gradually been discovered, such as the direct C–H functionalization reaction of arenes^14^ and alternative organometallic reagents.^8^ Rueping and Zhu,^15^ Gong,^16^ Hu,^17^ Lian,^18^ and others^19^ have achieved considerable progress in the field of carbonylative Negishi-type reactions, independently. However, the required organic halides are often not available and generally cannot be accessed in high selectivity from complex arenes. This problem can be perfectly solved by using aryl thianthrenium salts!

We investigated the carbonylative reaction of aryl thianthrenium salt TT-1a with benzyl chloride in the presence of a palladium catalyst and a metal agent (Table 1). Zinc was found to be crucial for the reaction, and other reagents such as manganese and iron did not produce any desired carbonylation product, which is consistent with the involvement of an organozinc intermediate (Table 1, entries 2 and 3). A preference for polar solvents such as MeCN was observed as larger amounts of unreacted initial materials remained when the reaction was conducted in less polar solvents, such as toluene (Table 1, entry 4), which could be explained by the faster rate of oxidative addition of zinc into benzyl chlorides in polar solvents.^20^ While nickel is the preferred transition metal for reductive aryl–alkyl bond formations,^21^ we identified palladium to be the catalyst of choice for this carbonylation of aryl thianthrenium salts. Various palladium catalysts have good effects on the reaction (see the ESI, Table S1,‡ entries 7–9). A series of bulky phosphine ligands were tested (see the ESI, Table S1,‡ entries 10–16). Due to the high reactivity of aryl thianthrenium salts, we attempted to use other inexpensive catalysts for the reaction, such as nickel and copper. Disappointingly, the target product was not detected (Table 1, entries 9 and 10). Surprisingly, when the pressure of carbon monoxide was decreased to 1 bar, the reaction still proceeded efficiently (Table 1, entry 11), which is still a challenge in transition metal-catalyzed carbonylation reactions.

**Table 1:** Optimization of reaction conditions^a^

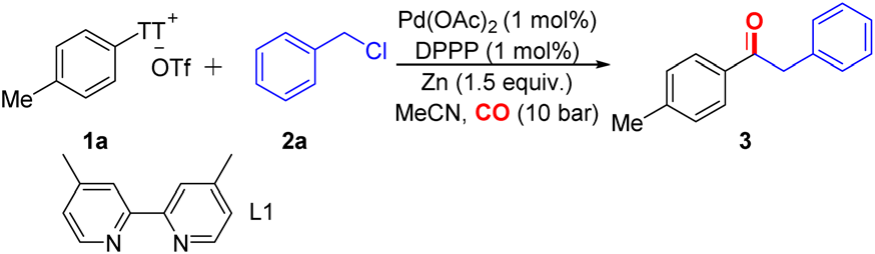		
Entry^a^	Variation from standard conditions	Yield^b^
1	None	99%^c^ (97%)
2	Mn instead of Zn	n.o.
3	Fe instead of Zn	n.o.
4	Toluene instead of MeCN	<5%
5	No catalyst	n.o.
6	No ligand	Trace
7	Pd(TFA)_2_ instead of Pd(OAc)_2_	94%
8	Xantphos instead of DPPP	31%
9	Ni(acac)_2_ + L1 as catalyst	n.o.
10	CuI + L1 as catalyst	n.o.
11	1 bar CO	91%

a Reaction conditions: 1a (0.2 mmol), 2a (0.3 mmol), CO (10 bar), Pd(OAc)_2_ (1 mol%), DPPP (1 mol%), Zn (0.3 mmol) in MeCN (2 mL) at 80 °C for 20 h.

b Yields were determined by GC-FID analysis using *n*-dodecane as the internal standard.

c Yield of the isolated product. n.o. = not observed.

**Scheme 2:**
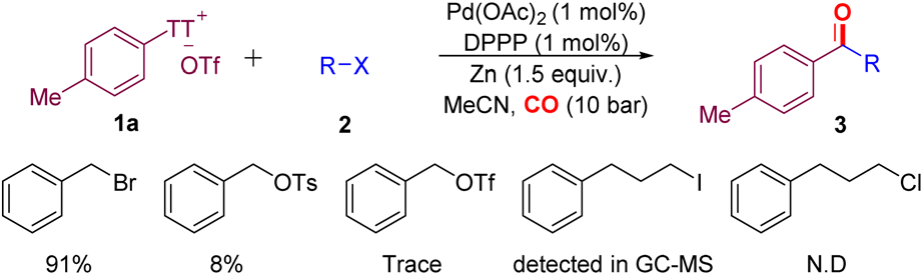
Testing of alkyl (pseudo)halides.

Different alkyl halides were tested under the optimal conditions in the presence of zinc powder (Scheme 2). Benzyl bromide can achieve an isolated yield of 91%. At the same time, some leaving groups, such as OTs and OTf, were also checked. The results showed that when the leaving group was replaced, the reaction could not proceed effectively, even in the presence of an additional amount of NaI or NaBr. Surprisingly, when using long-chain alkyl iodide, we can still detect the target product on GC-MS (see the ESI,‡ pp S8 for details). The lack of reactivity of alkyl chloride was also expected.

**Scheme 3:**
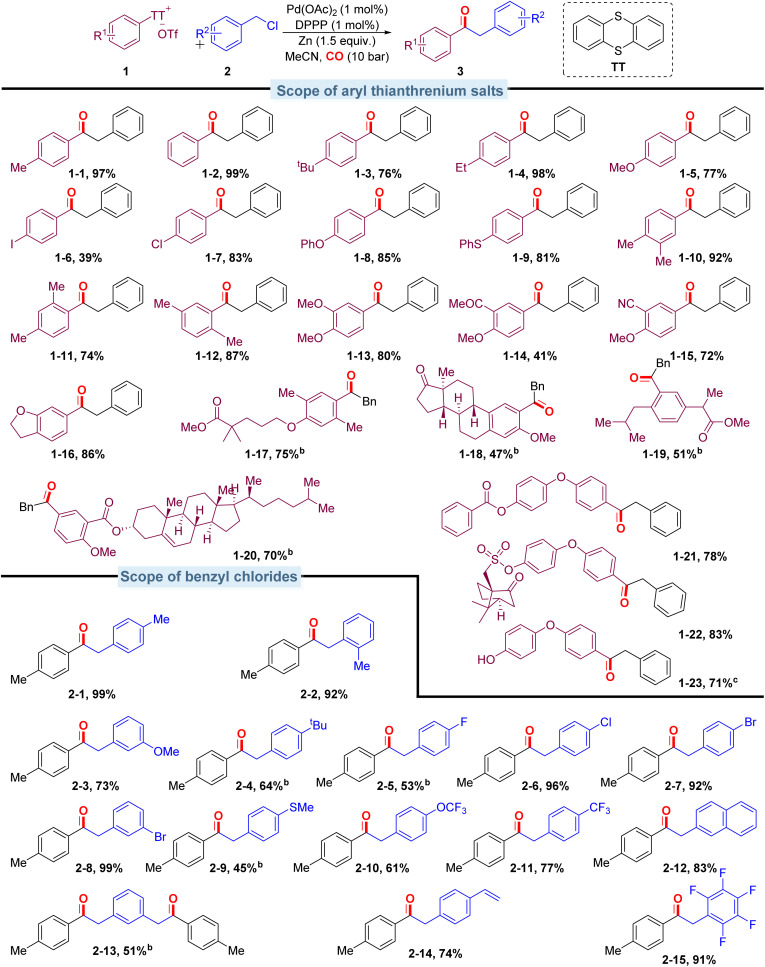
Scope of different aryl thianthrenium salts and benzyl chlorides. ^a^Reaction conditions: aryl thianthrenium salts (0.2 mmol), benzyl chlorides (0.3 mmol), Pd(OAc)_2_ (1 mol%), DPPP (1 mol%), Zinc powder (0.3 mmol), MeCN (2 mL), CO (10 bar), 80 °C, 20 h, isolated yields. ^b^48 h. ^c^With 5-(4-(4-((6-methylpicolinoyl)oxy)phenoxy)phenyl)-5*H*-thianthren-5-ium as the substrate.

With the optimized conditions in hand, we evaluated the scope of aryl thianthrenium salts by reacting them with benzyl chloride (Scheme 3). This method shows excellent compatibility with a broad range of aryl thianthrenium salts, and the corresponding products were isolated in moderate to excellent yields. Aryl thianthrenium salts bearing electron-neutral substituents (Scheme 3, 1-2) and monosubstituted groups including methyl, *tert*-butyl, ethyl, methoxy, iodo-, and chloro- (Scheme 3, 1-1, 1-3–1-7) furnished the corresponding products with moderate to good yields. Aryl thianthrenium salts bearing phenoxy or thiophenyl substituents (Scheme 3, 1-8, 1-9) reacted well and efficiently in this palladium-catalyzed carbonylation protocol. Various disubstituted substrates (Scheme 3, 1-10–1-15) also underwent the targeted carbonylative transformation successfully and gave the corresponding products in moderate to good yields. Functional groups such as cyano and acetyl groups were also compatible under the optimized conditions, and the corresponding products were produced without any problem (Scheme 3, 1-14, 1-15). The substrate based on benzofuran can gave the targeted product in excellent isolated yield as well (Scheme 3, 1-16). To further illustrate the utility of this carbonylation strategy, we sought to integrate the carbonylation approach into the diversification of complex molecules; carbonyls were subsequently installed into various pharmaceutically relevant heterocyclic substrates containing commercial natural estrogen (Scheme 3, 1-18, from estrone), commercial drug derivatives (Scheme 3, 1-17, from Gemfibrozil, Scheme 3, 1-19, from ibuprofen), natural products (Scheme 3, 1-20, from cholesterol), and others (Scheme 3, 1-21–1-23) in reasonably good to excellent yields.

The carbonylation protocol also displayed good compatibility across a range of benzyl chlorides. As shown in Scheme 3, when 4-methylbenzyl chloride was introduced, the product (Scheme 3, 2-1) was isolated in 99% yield. *Ortho*-methyl-substituted benzyl chloride gave the corresponding ketone (Scheme 3, 2-2) in good yield. Both electron-donating and electron-withdrawing substituted benzyl chlorides were successfully transformed into the desired ketones in good yields (45–99%; Scheme 3, 2-3–2-11). The reason for the lower yield of benzyl chloride substituted with methylthio- and *tert*-butyl is that the substrate has not been completely converted. Extending the reaction time can improve the yield, and the direct coupling products do not increase due to the extension of time. Additionally, the naphthalene motif could also be successfully incorporated into the desired products with excellent efficiency (Scheme 3, 2-12). In particular, 1,3-bis(chloroethyl)benzene modified with dual reaction sites is also compatible, and the yield was up to 51%. As the reactions proceed under mild conditions, sensitive functionalities alkene-substituent and penta-fluoro-substituent are all tolerated (Scheme 3, 2-14–2-15).

**Scheme 4:**
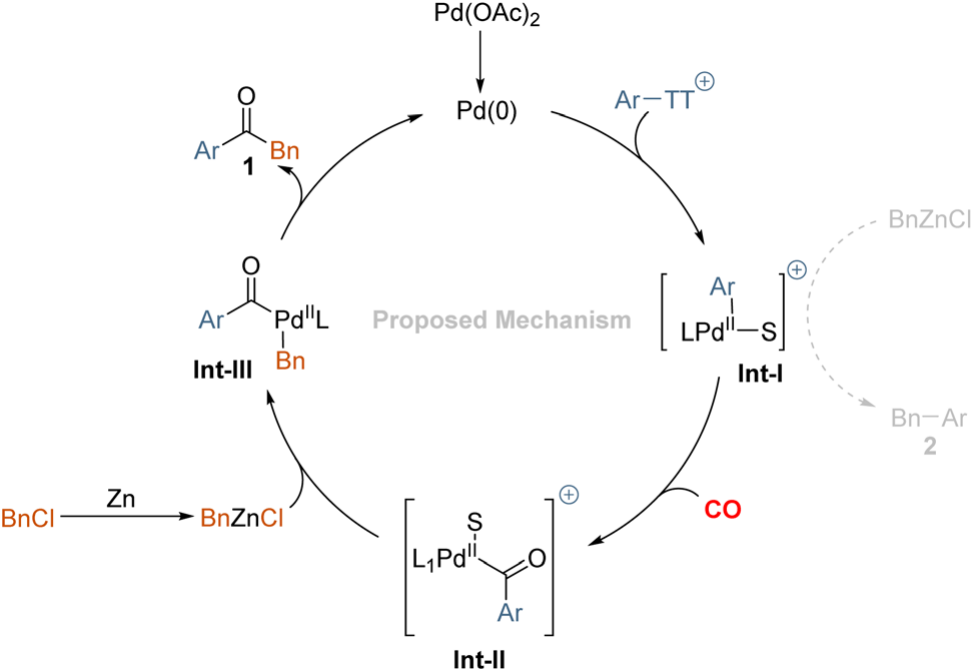
Proposed mechanism.

Based on these results, the following reaction mechanism is proposed in Scheme 4. We believe an oxidative addition-transmetalation-reductive elimination sequence is operative in this transformation. In principle, the active palladium (0) catalyst, as well as the zinc species, might undergo addition to the aryl thianthrenium salts and the benzyl chloride. Although the reduction potential of benzyl chloride is lower than that of aryl thianthrenium salts, that will lead to the reduction of aryl thianthrenium salts more easily. According to literature research, the oxidation addition of zinc is carried out through an inner sphere electron transfer involving a bridging ligand.^22^ Hence, we conclude that the desired product 1 is mainly produced *via* the oxidative addition transmetalation-reductive elimination sequence. After oxidative addition of palladium(0) to aryl thianthrenium salts, the insertion of carbon monoxide takes place to give the acylpalladium complex. Subsequent reaction with the *in situ* formed benzylic zinc reagent provides 1,2-diphenylmethane after reductive elimination. Byproduct 2, which resulted primarily from Int-I undergoes transmetalation directly with the *in situ* generated organic zinc reagent.

## Conclusions

In conclusion, we have developed a site-selective carbonylation method for carbonylation of aryl thianthrenium salts, which allows the production of 1,2-diarylethanones that cannot be obtained with similar efficiency by other carbonylation methods. The excellent site-selectivity and robust reactivity enable us to engage complex fragments, which could be of value in medicinal chemistry. We believe this work represents a valuable conceptual extension to existing carbonylation cross-coupling reactions with improved efficiency, reactivity, and synthetic utility.

## Data availability

All data supporting the findings of this study are available within the article and its ESI file.‡

## Author contributions

X.-F. W. conceived this project. J. Z., L.-C. W. and Z.-P. B. performed all the experiments and prepared the ESI.‡ X.-F. W. and J. Z. wrote and revised the manuscript.

## Conflicts of interest

There are no conflicts to declare.

## Supplementary Material

SC-014-D3SC02402D-s001
