# Supplementary material for: Site-selective carbonylation of arenes via C(sp2)–H thianthrenation: direct access to 1,2-diarylethanones
Source: Chem Sci. 2023 Jun 16;14(28):7637–41. doi: 10.1039/d3sc02402d (PMC10355103; doi:10.1039/d3sc02402d)

Electronic Supplementary Material (ESI) for Chemical Science.
This journal is © The Royal Society of Chemistry 2023

Supporting Information

Site-Selective Carbonylation of Arenes via C(sp^2)-H Thianthrenation: Direct Access to 1,2-Diarylethanones

Jiajun Zhang,[a] [b] Le-Cheng Wang,[a] [b] Zhi-Peng Bao,[a] [b] Xiao-Feng Wu*[a] [b]

[a] Dalian National Laboratory for Clean Energy, Dalian Institute of Chemical Physics, Chinese Academy of Sciences, 116023 Dalian, Liaoning, China

[b] Leibniz-Institut für Katalyse e. V., Albert-Einstein-Straβe 29a, 18059 Rostock, Germany

xwu2020@dicp.ac.cn; Xiao-Feng.Wu@catalysis.de

Table of Contents

1. General Information S2
2. Synthesis of the starting materials S3
3. General Procedure S5
4. Testing of alkyl (pseudo)halides S6
5. Characterization of Products S8
6. References S18
7. Copy of ^{1}H and ^{13}C NMR Spectra of Products S19

1. General Information

Unless otherwise noted, all reactions were carried out under a carbon monoxide or nitrogen atmosphere. All reagents were from commercial sources, all solvents are extra dry solvents and used as received without further purification. Column chromatography was performed on silica gel (200-300 meshes) using petroleum ether (b.p. 60-90 ^{o}C) and ethyl acetate as the eluents. ^{1}H and ^{13}C NMR spectra were taken on Bruker AVANCE III 400 MHz or 700 MHz spectrometers and spectral data were reported in ppm relative to tetramethylsilane (TMS) as the internal standard and $CDCl_3$ or DMSO-D_6 as solvent. All coupling constants (J) are reported in Hz with the following abbreviations: s = singlet, d = doublet, dd = double doublet, t = triplet, dt = double triplet, q = quatriplet, m = multiplet, br = broad. Gas chromatography (GC) analyses were performed on an Agilent HP-7890A instrument with a FID detector and HP-5 capillary column (polydimethylsiloxane with 5% phenyl groups, 30 m, 0.32 mm i.d. 0.25 μm film thickness) using argon as carrier gas. Gas chromatography mass spectrometer (GC-MS) analyses were performed on a Shimadzu QP2020 NX instrument. Because of the high toxicity of carbon monoxide, all of the reactions should be performed in an autoclave. The laboratory should well-equipped with a CO detector and alarm system.

2. Synthesis of the starting materials

(1)

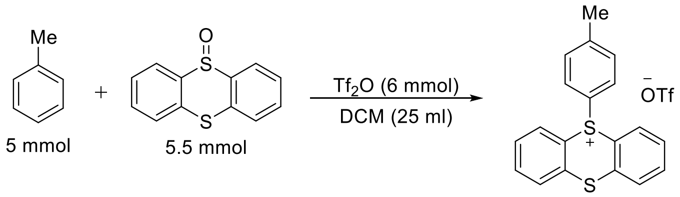

The known 5-(*p*-tolyl)-5*H*-thianthren-5-ium trifluoromethanesulfonate was synthesized according to the literature[1a]. A 100 mL schlenk tube was charged with thianthrene S-oxide (1.276 g, 5.5 mmol, 1.1 equiv.), DCM (25 mL) and anisole (540 mg, 5 mmol, 1.0 equiv.) under a nitrogen atmosphere. The reaction mixture was then cooled to -40 °C and stirred at this temperature. Tf_2O (6 mmol, 1.2 equiv.) was added dropwise. The reaction mixture was stirred at -40 °C for 30 min, and then allowed to stir at room temperature for 12 h, neutralized by a saturated aqueous $NaHCO_3$ solution, and extracted with DCM. The combined organic layers were dried over anhydrous Na_2SO_4 and concentrated to dryness under reduced pressure. The crude product was purified by crystallization from DCM / diethyl ether system to afford 5-(*p*-tolyl)-5*H*-thianthren-5-ium trifluoromethanesulfonate as a white solid.

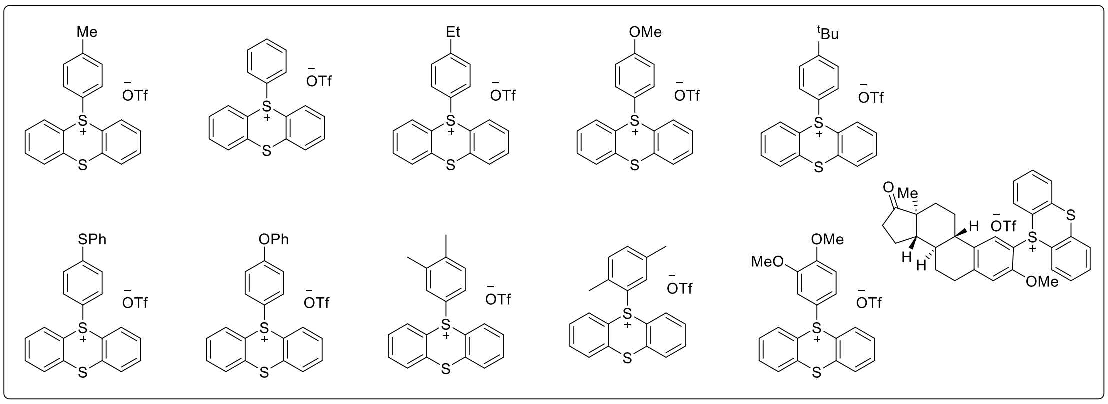

(2)

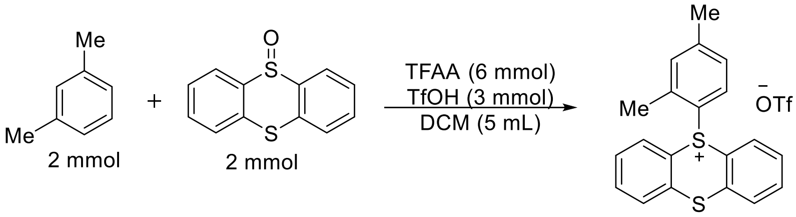

The known aryl sulfonium salts 5-(2,4-Dimethylphenyl)-5*H*-thianthren-5-ium trjifluoromethanesulfonate was synthesized according to the literature[1a]. A 25 mL schlenk tube was charged with thianthrene S-oxide (464 mg, 2 mmol, 1 equiv), DCM (5 mL) and *m*-xylene (212 mg, 2 mmol, 1.0 equiv) under a nitrogen atmosphere. The reaction mixture was then cooled to -40 °C and stirred at this temperature. trifluoroacetic anhydride (TFAA, 6 mmol, 3.0 equiv) and trifluoromethanesulfonic acid (TfOH, 3 mmol, 1.5 equiv) were added dropwise. The reaction mixture was stirred at 40 °C for 30 min, and then allowed to stir at room temperature for 12 h, neutralized by a saturated aqueous $NaHCO_3$ solution, and extracted with DCM. The combined organic layers were washed with aqueous NaOTf solution (3 × 20 mL, 5% (w/w)), dried over anhydrous Na_2SO_4, and concentrated to dryness under reduced pressure. The crude product was purified by crystallization from DCM / diethyl ether system to afford 5-(2,4-Dimethylphenyl)-5*H*-thianthren-5-ium

trjifluoromethanesulfonate as a white solid.

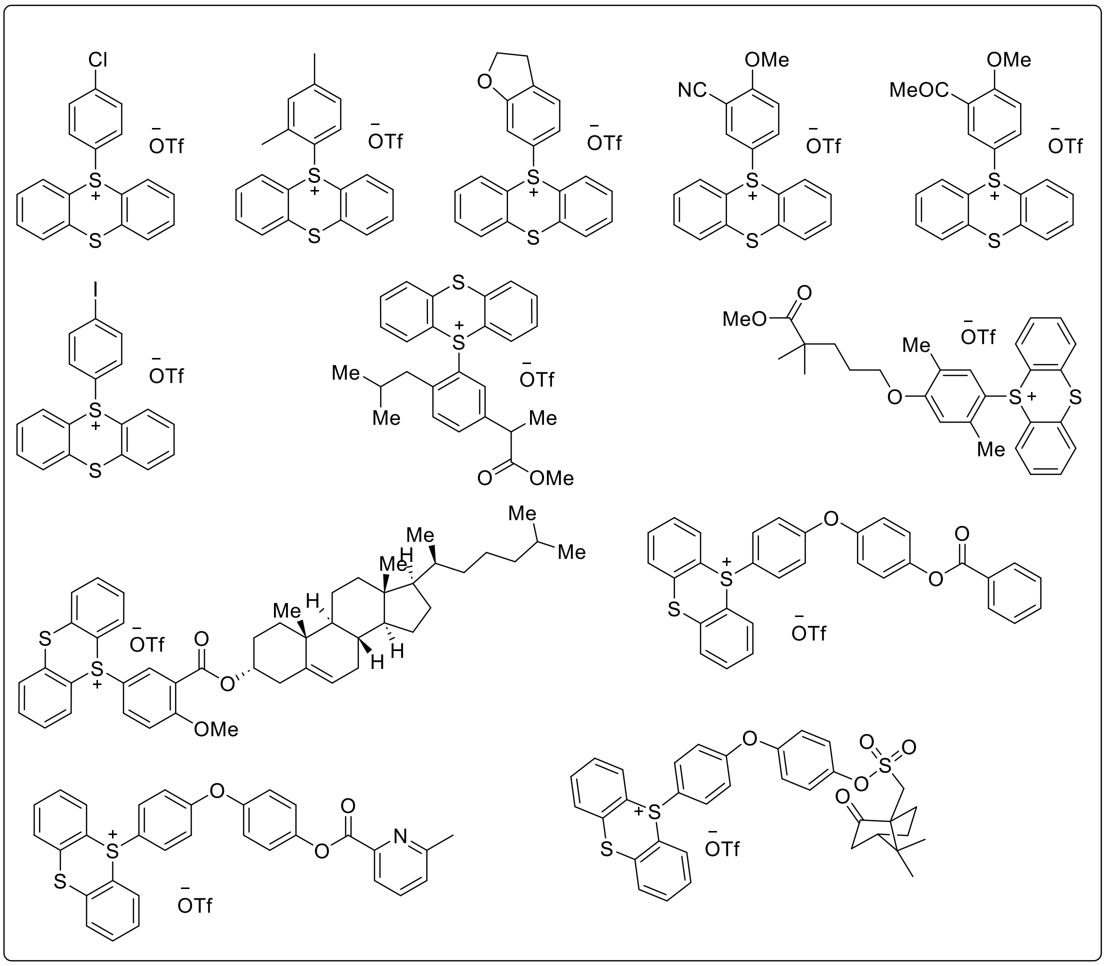

(3) Preparation of Benzyl Chlorides

General procedure:[1b] To a stirring solution of the corresponding benzyl alcohol (10 mmol), N,N-dimethylformamide (20 μL) and CH_2Cl_2 (20 mL) were added thionyl chloride (12 mmol) dropwise at 0 °C. After addition, the mixture was allowed to stir at room temperature for 1 h. The complete consumption of the benzyl alcohol was verified by TLC or GC. Then the mixture was poured into saturated $NaHCO_3$ (20 mL), and extracted with dichloromethane (20 mL × 3). The combined organic layer was washed with water (20 mL), brine (20 mL), then dried over MgSO4, filtered, and concentrated under vacuum. The crude product was purified by silica gel chromatography.

3. General Procedure

(1) **Table S1**: Optimization of yield as a function of catalyst

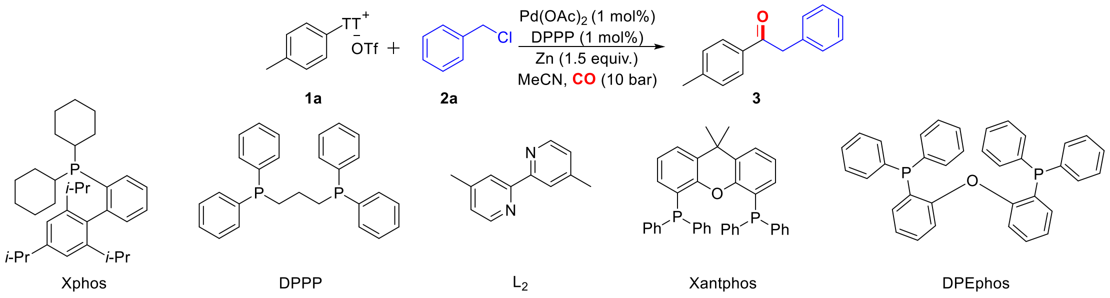

Entry	Catalyst	Yield[b]
1	No catalyst	n.o.
2	No Ligand	n.o.
3	CuI + L_2	n.o.
4	$Ni(acac)_2$ + L_2	n.o.
5	$Co(acac)_2$ + L_2	n.o.
6	$Pd(OAc)_2$ + DPPP	99% (97%)
7	$PdCl_2$ + DPPP	95%
8	$Pd(TFA)_2$ + DPPP	94%
9	PdI_2 + DPPP	95%
10	$Pd(OAc)_2$ + Xantphos	31%
11	$Pd(OAc)_2$ + DPPF	96%
12	$Pd(OAc)_2$ + PPh_3 (2%)	30%
13	$Pd(OAc)_2$ + TFP	Trace
14	$Pd(OAc)_2$ + XPhos	Trace
15	$Pd(OAc)_2$ + DPEphos	32%
16	$Pd(OAc)_2$ + BINAP	41%

n.o. = not observed.

(2) General procedure for carbonylative of **aryl sulfonium salts**.

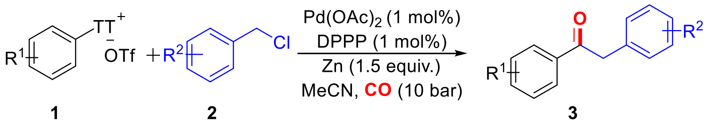

A 4 mL screw-cap vial was charged with $Pd(OAc)_2$ (1 mol%, 0.5 mg), DPPP (1 mol%, 0.8 mg), zinc powder (0.30 mmol, 19.6 mg), aryl sulfonium salts **1** (0.2 mmol), benzyl chlorides **2** (0.30 mmol) and an oven-dried stirring bar. The vial was closed with a Teflon septum and cap and connected to the atmosphere via a needle. Then MeCN (2 mL) was added with a syringe under N_2 atmosphere, the vial was moved to an alloy plate and put into a Parr 4560 series autoclave (300 mL) under N_2 atmosphere. At room temperature, the autoclave was flushed with CO three times and charged with 10 bar CO. The autoclave was placed on a heating plate equipped with a magnetic stirrer. The reaction mixture was heated to 80 °C for 20 h. After the reaction was completed, the crude mixture was filtered and concentrated under vacuum. The crude product was purified by column chromatography (PE/EA =1/0 to 20/1) on silica gel to afford the corresponding products.

(3) General procedure for carbonylative of **benzyl chlorides**.

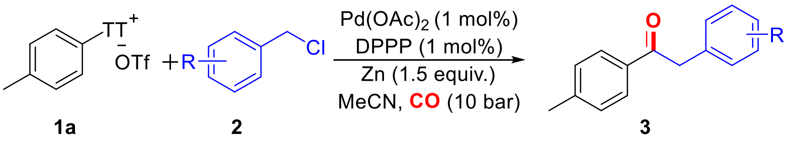

A 4 mL screw-cap vial was charged with $Pd(OAc)_2$ (1 mol%, 0.5 mg), DPPP (1 mol%, 0.8 mg), zinc powder (0.30 mmol, 19.6 mg), aryl sulfonium salts **1a** (0.2 mmol, 91.2 mg), benzyl chlorides **2** (0.30 mmol) and an oven-dried stirring bar. The vial was closed with a Teflon septum and cap and connected to the atmosphere via a needle. Then MeCN (2 mL) was added with a syringe under N_2 atmosphere, the vial was moved to an alloy plate and put into a Parr 4560 series autoclave (300 mL) under N_2 atmosphere. At room temperature, the autoclave was flushed with CO three times and charged with 10 bar CO. The autoclave was placed on a heating plate equipped with a magnetic stirrer. The reaction mixture was heated to 80 ºC for 20 h. After the reaction was completed, the crude mixture was filtered and concentrated under vacuum. The crude product was purified by column chromatography (PE/EA =1/0 to 20/1) on silica gel to afford the corresponding products.

4. Testing of alkyl (pseudo)halides

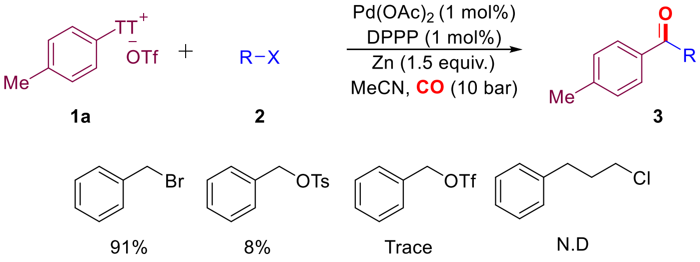

A 4 mL screw-cap vial was charged with $Pd(OAc)_2$ (1 mol%, 0.5 mg), DPPP (1 mol%, 0.8 mg), zinc powder (0.30 mmol, 19.6 mg), aryl sulfonium salts **1a** (0.2 mmol, 91.2 mg), alkyl halides **2** (0.30 mmol) and an oven-dried stirring bar. The vial was closed with a Teflon septum and cap and connected to the atmosphere via a needle. Then MeCN (2 mL) was added with a syringe under N_2 atmosphere, the vial was moved to an alloy plate and put into a Parr 4560 series autoclave (300 mL) under N_2 atmosphere. At room temperature, the autoclave was flushed with CO three times and charged with 10 bar CO. The autoclave was placed on a heating plate equipped with a magnetic stirrer. The reaction mixture was heated to 80 ºC for 20 h. After the reaction was completed, the crude mixture was filtered and concentrated under vacuum. The crude product was purified by column chromatography (PE/EA =1/0 to 20/1) on silica gel to afford the corresponding products.

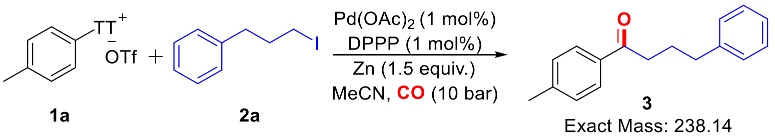

A 4 mL screw-cap vial was charged with $Pd(OAc)_2$ (1 mol%, 0.5 mg), DPPP (1 mol%, 0.8 mg), zinc powder (0.30 mmol, 19.6 mg), aryl sulfonium salts **1a** (0.2 mmol, 91.2 mg), 1-iodo-3-phenylpropane **3** (0.30 mmol) and an oven-dried stirring bar. The vial was closed with a Teflon septum and cap and connected to the atmosphere via a needle. Then MeCN (2 mL) was added with a syringe under N_2 atmosphere, the vial was moved to an alloy plate and put into a Parr 4560 series autoclave (300 mL) under N_2 atmosphere. At room temperature, the autoclave was flushed with CO three times and charged

with 10 bar CO. The autoclave was placed on a heating plate equipped with a magnetic stirrer. The reaction mixture was heated to 80 °C for 20 h. After the reaction was completed, the reaction solution was detected by GC-MS. The detection results are shown in the figure below, and the molecular weight of the target molecule was detected at a retention time of 12.585 minutes.

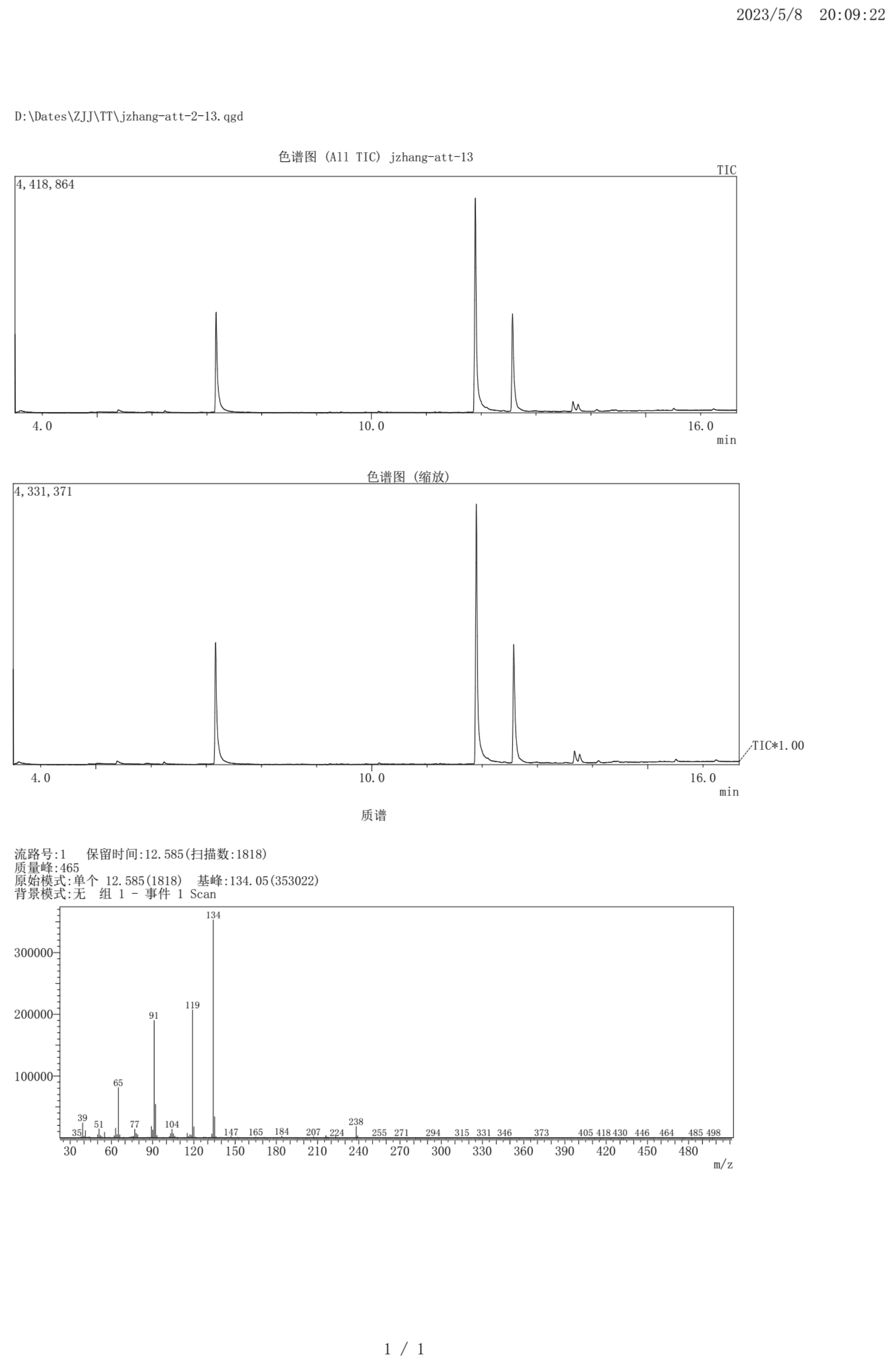

5. Characterization of Products

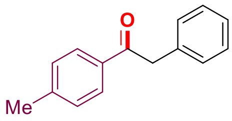

2-phenyl-1-(*p*-tolyl)ethan-1-one (**1-1**)[2]

40.8 mg, White solid, yield: 97%. Eluent: pentane/ethyl acetate = 1/0 to 20/1.

^{1}H NMR (400 MHz, $CDCl_3$) δ 7.91 (d, *J* = 8.3 Hz, 2H), 7.34 – 7.21 (m, 7H), 4.24 (s, 2H), 2.39 (s, 3H).

^{13}C NMR (101 MHz, $CDCl_3$) δ 197.3, 144.0, 134.8, 134.2, 129.5, 129.4, 128.8, 128.7, 126.8, 45.4, 21.7.

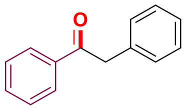

1,2-diphenylethan-1-one (**1-2**)[2]

38.8 mg, white solid, yield: 99 %. Eluent: pentane/ethyl acetate = 1/1 to 20/1.

^{1}H NMR (700 MHz, $CDCl_3$) δ 8.01 (d, *J* = 7.0 Hz, 2H), 7.55 (t, *J* = 7.4 Hz, 1H), 7.45 (t, *J* = 7.8 Hz, 2H), 7.32 (t, *J* = 7.6 Hz, 2H), 7.28 – 7.24 (m, 3H), 4.28 (s, 2H).

^{13}C NMR (176 MHz, $CDCl_3$) δ 197.6, 136.6, 134.6, 133.2, 129.5, 128.7, 128.7, 128.6, 126.9, 45.5.

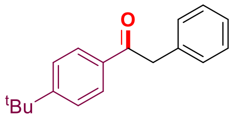

1-(4-(*tert*-butyl)phenyl)-2-phenylethan-1-one (**1-3**)[2]

38.3 mg, white solid, yield: 76 %. Eluent: pentane/ethyl acetate = 1/0 to 20/1.

^{1}H NMR (700 MHz, $CDCl_3$) δ 7.95 (d, *J* = 8.1 Hz, 2H), 7.46 (d, *J* = 8.1 Hz, 2H), 7.31 (t, *J* = 7.5 Hz, 2H), 7.27 (d, *J* = 7.6 Hz, 2H), 7.24 (d, *J* = 7.6 Hz, 1H), 4.25 (s, 2H), 1.32 (s, 9H).

^{13}C NMR (176 MHz, $CDCl_3$) δ 197.26, 156.93, 134.83, 134.06, 129.49, 128.68, 128.66, 126.84, 125.64, 45.48, 35.15, 31.11.

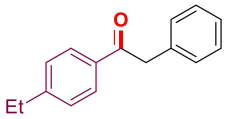

1-(4-ethylphenyl)-2-phenylethan-1-one (**1-4**)[4]

43.9 mg, White solid, yield: 98%. Eluent: pentane/ethyl acetate = 1/0 to 20/1.

^{1}H NMR (700 MHz, $CDCl_3$) δ 7.94 (d, *J* = 8.3 Hz, 2H), 7.31 (t, *J* = 7.7 Hz, 2H), 7.29 – 7.21 (m, 5H), 4.25 (s, 2H), 2.69 (q, *J* = 7.7 Hz, 2H), 1.24 (t, *J* = 7.7 Hz, 3H).

^{13}C NMR (176 MHz, $CDCl_3$) δ 197.3, 150.2, 134.8, 134.4, 129.5, 128.9, 128.7, 128.2, 126.8, 45.5, 29.0, 15.2.

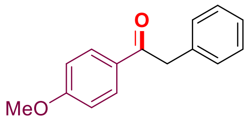

1-(4-methoxyphenyl)-2-phenylethan-1-one (**1-5**)[2]

34.8 mg, White solid, yield: 77 %. Eluent: pentane/ethyl acetate = 1/0 to 20/1.

^{1}H NMR (400 MHz, CDCl$_3$) δ 7.99 (d, J = 8.9 Hz, 2H), 7.34 – 7.21 (m, 5H), 6.92 (d, J = 8.9 Hz, 2H), 4.23 (s, 2H), 3.85 (s, 3H).

^{13}C NMR (101 MHz, CDCl$_3$) δ 196.2, 163.5, 135.0, 131.0, 129.7, 129.4, 128.7, 126.8, 113.8, 55.5, 45.3.

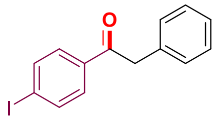

1-(4-iodophenyl)-2-phenylethan-1-one (**1-6**)[3]

25.1 mg, white solid, yield: 39 %. Eluent: pentane/ethyl acetate = 1/0 to 20/1.

^{1}H NMR (400 MHz, CDCl$_3$) δ 7.80 (d, *J* = 8.5 Hz, 2H), 7.69 (d, *J* = 8.5 Hz, 2H), 7.31 (t, *J* = 7.1 Hz, 2H), 7.26 – 7.20 (m, 3H), 4.22 (s, 2H).

^{13}C NMR (101 MHz, CDCl$_3$) δ 196.9, 138.0, 135.8, 134.1, 130.0, 129.4, 128.8, 127.1, 101.2, 45.5.

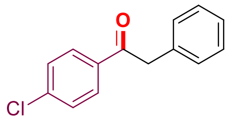

1-(4-chlorophenyl)-2-phenylethan-1-one (**1-7**)[2]

38.2 mg, white solid, yield: 83 %. Eluent: pentane/ethyl acetate = 1/0 to 20/1.

^{1}H NMR (700 MHz, CDCl$_3$) δ 7.94 (d, *J* = 8.2 Hz, 2H), 7.42 (d, *J* = 8.2 Hz, 2H), 7.33 (t, *J* = 7.5 Hz, 2H), 7.25 (d, *J* = 9.9 Hz, 3H), 4.25 (s, 2H).

^{13}C NMR (176 MHz, CDCl$_3$) δ 196.4, 139.6, 134.9, 134.2, 130.1, 129.4, 129.0, 128.8, 127.1, 45.6.

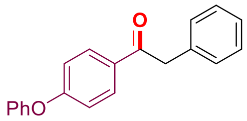

1-(4-phenoxyphenyl)-2-phenylethan-1-one (**1-8**)[5]

49.0 mg, white solid, yield: 85 %. Eluent: pentane/ethyl acetate = 1/0 to 20/1.

^{1}H NMR (700 MHz, CDCl$_3$) δ 7.98 (d, *J* = 8.9 Hz, 2H), 7.40 – 7.35 (m, 2H), 7.31 (t, *J* = 7.6 Hz, 2H), 7.27 – 7.21 (m, 3H), 7.19 (t, *J* = 7.4 Hz, 1H), 7.05 (d, *J* = 7.5 Hz, 2H), 6.97 (d, *J* = 8.9 Hz, 2H), 4.22 (s, 2H).

^{13}C NMR (176 MHz, CDCl$_3$) δ 196.2, 162.1, 155.4, 134.8, 131.2, 131.0, 130.1, 129.4, 128.7, 126.9, 124.7, 120.3, 117.3, 45.4.

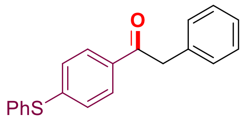

2-phenyl-1-(4-(phenylthio)phenyl)ethan-1-one (**1-9**)

49.3 mg, white solid, yield: 81 %. Eluent: pentane/ethyl acetate = 1/0 to 20/1.

^{1}H NMR (700 MHz, CDCl$_3$) δ 7.86 (d, *J* = 8.2 Hz, 2H), 7.52 – 7.45 (m, 2H), 7.39 (q, *J* = 3.6, 2.2 Hz, 3H), 7.30 (t, *J* = 7.6 Hz, 2H), 7.24 (d, *J* = 7.3 Hz, 3H), 7.18 (d, *J* = 8.2 Hz, 2H), 4.21 (s, 2H).

^{13}C NMR (176 MHz, CDCl$_3$) δ 196.6, 145.2, 134.6, 134.1, 133.8, 131.9, 129.7, 129.4, 129.3, 128.9, 128.7, 127.3, 126.9, 45.4.

HRMS (ESI-TOF) m/z: $[M+H]^+$ Calcd. for $C_{20}H_{17}OS$ 305.0995; found: 305.0995.

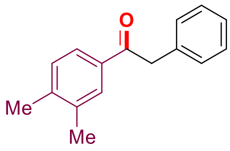

1-(3,4-dimethylphenyl)-2-phenylethan-1-one (**1-10**)[4]

41.2 mg, white solid, yield: 92 %. Eluent: pentane/ethyl acetate = 1/0 to 20/1.

^{1}H NMR (700 MHz, CDCl$_3$) δ 7.79 (s, 1H), 7.74 (d, *J* = 7.1 Hz, 1H), 7.30 (t, *J* = 7.6 Hz, 2H), 7.26 (d, *J* = 7.6 Hz, 2H), 7.23 (t, *J* = 7.2 Hz, 1H), 7.19 (d, *J* = 7.8 Hz, 1H), 4.24 (s, 2H), 2.29 (s, 6H).

^{13}C NMR (176 MHz, CDCl$_3$) δ 197.6, 142.8, 137.0, 134.9, 134.6, 129.9, 129.8, 129.5, 128.7, 126.8, 126.5, 45.4, 20.1, 19.8.

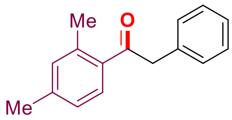

1-(2,4-dimethylphenyl)-2-phenylethan-1-one (**1-11**)[6]

33.2 mg, colorless oil, yield: 74 %. Eluent: pentane/ethyl acetate = 1/0 to 20/1.

^{1}H NMR (700 MHz, CDCl$_3$) δ 7.67 (d, *J* = 7.8 Hz, 1H), 7.30 (t, *J* = 7.7 Hz, 2H), 7.22 (d, *J* = 8.3 Hz, 3H), 7.08 – 7.02 (m, 2H), 4.19 (s, 2H), 2.44 (s, 3H), 2.33 (s, 3H).

^{13}C NMR (176 MHz, CDCl$_3$) δ 200.7, 142.1, 139.3, 134.9, 134.5, 133.0, 129.5, 129.4, 128.6, 126.8, 126.3, 48.1, 21.6, 21.4.

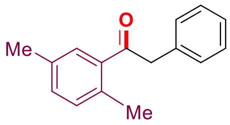

1-(2,5-dimethylphenyl)-2-phenylethan-1-one (**1-12**)[7]

39.0 mg, white solid, yield: 87 %. Eluent: pentane/ethyl acetate = 1/0 to 20/1.

^{1}H NMR (700 MHz, CDCl$_3$) δ 7.51 (s, 1H), 7.31 (t, *J* = 7.6 Hz, 2H), 7.26 – 7.21 (m, 3H), 7.16 (d, *J* = 7.0 Hz, 1H), 7.10 (d, *J* = 7.7 Hz, 1H), 4.19 (s, 2H), 2.38 (s, 3H), 2.35 (s, 3H).

^{13}C NMR (176 MHz, CDCl$_3$) δ 201.6, 137.7, 135.3, 135.1, 134.6, 132.1, 131.9, 129.6, 129.2, 128.6, 126.9, 48.4, 21.0, 20.8.

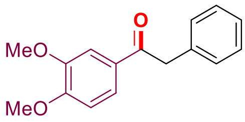

1-(3,4-dimethoxyphenyl)-2-phenylethan-1-one (**1-13**)[8]

40.9 mg, yellow oil, yield: 80 %. Eluent: pentane/ethyl acetate = 1/0 to 20/1.

^{1}H NMR (700 MHz, CDCl$_3$) δ 7.66 (d, *J* = 7.9 Hz, 1H), 7.56 (d, *J* = 2.1 Hz, 1H), 7.32 (t, *J* = 7.5 Hz, 2H), 7.27 (d, *J* = 7.5 Hz, 2H), 7.24 (t, *J* = 7.2 Hz, 1H), 6.87 (d, *J* = 8.4 Hz, 1H), 4.24 (s, 2H), 3.93 (s, 3H), 3.91 (s, 3H).

^{13}C NMR (176 MHz, CDCl$_3$) δ 196.3, 153.3, 149.1, 135.1, 129.8, 129.3, 128.7, 126.8, 123.5, 110.7, 110.0, 56.1, 56.0, 45.2.

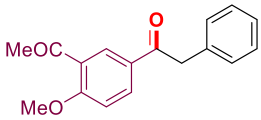

1-(3-acetyl-4-methoxyphenyl)-2-phenylethan-1-one (**1-14**)

22.0 mg, white solid, yield: 41 %. Eluent: pentane/ethyl acetate = 1/0 to 20/1.

^{1}H NMR (700 MHz, CDCl$_3$) δ 8.43 (d, *J* = 2.4 Hz, 1H), 8.17 – 8.13 (m, 1H), 7.31 (t, *J* = 7.6 Hz, 2H), 7.27 (d, *J* = 7.4 Hz, 2H), 7.24 (t, *J* = 7.2 Hz, 1H), 7.03 (d, *J* = 8.8 Hz, 1H), 4.26 (s, 2H), 3.98 (s, 3H), 2.62 (s, 3H).

^{13}C NMR (176 MHz, CDCl$_3$) δ 198.7, 195.9, 162.4, 134.5, 134.3, 131.7, 129.5, 129.4, 128.7, 127.8, 126.9, 111.8, 56.0, 45.3, 31.8.

HRMS (ESI-TOF) m/z: [M+Na]$^+$ Calcd. for $C_{17}H_{16}O_3Na$ 291.0992; found: 291.0985.

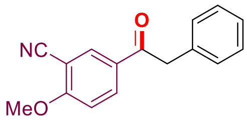

2-methoxy-5-(2-phenylacetyl)-benzonitrile (**1-15**)

36.2 mg, white solid, yield: 72 %. Eluent: pentane/ethyl acetate = 1/0 to 20/1.

^{1}H NMR (700 MHz, CDCl$_3$) δ 8.22 (d, *J* = 2.2 Hz, 1H), 8.21 – 8.18 (m, 1H), 7.33 (t, *J* = 7.5 Hz, 2H), 7.27 (d, *J* = 7.5 Hz, 1H), 7.24 (d, *J* = 7.7 Hz, 2H), 7.02 (d, *J* = 8.8 Hz, 1H), 4.22 (s, 2H), 3.99 (s, 3H).

^{13}C NMR (176 MHz, CDCl$_3$) δ 194.5, 164.3, 135.2, 134.9, 133.9, 129.6, 129.3, 128.9, 127.2, 115.5, 111.3, 102.3, 56.6, 45.3.

HRMS (ESI-TOF) m/z: [M+H]$^+$ Calcd. for $C_{16}H_{14}NO_2$ 252.1019; found: 252.1013.

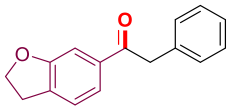

1-(2,3-dihydrobenzofuran-6-yl)-2-phenylethan-1-one (**1-16**)

40.9 mg, yellow solid, yield: 86 %. Eluent: pentane/ethyl acetate = 1/0 to 20/1.

^{1}H NMR (400 MHz, CDCl$_3$) δ 7.89 (s, 1H), 7.86 (d, J = 8.4 Hz, 1H), 7.35 – 7.20 (m, 5H), 6.79 (d, J = 8.4 Hz, 1H), 4.64 (t, J = 8.8 Hz, 2H), 4.21 (s, 2H), 3.23 (t, J = 8.7 Hz, 2H).

^{13}C NMR (101 MHz, CDCl$_3$) δ 196.1, 164.5, 135.1, 130.7, 130.0, 129.4, 128.6, 127.8, 126.8, 125.9, 109.0, 72.2, 45.3, 29.0.

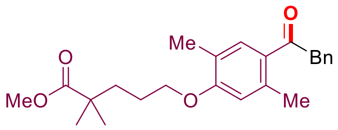

methyl 5-(2,5-dimethyl-4-(2-phenylacetyl)phenoxy)-2,2-dimethylpentanoate (**1-17**)

57.3 mg, white solid, yield: 75 %. Eluent: pentane/ethyl acetate = 1/0 to 20/1.

^{1}H NMR (400 MHz, CDCl$_3$) δ 8.22 (d, *J* = 2.2 Hz, 1H), 8.21 – 8.18 (m, 1H), 7.33 (t, *J* = 7.5 Hz, 2H), 7.27 (d, *J* = 7.5 Hz, 1H), 7.24 (d, *J* = 7.7 Hz, 2H), 7.02 (d, *J* = 8.8 Hz, 1H), 4.22 (s, 2H), 3.99 (s, 3H).

^{13}C NMR (101 MHz, CDCl$_3$) δ 194.5, 164.3, 135.2, 134.9, 133.9, 129.6, 129.3, 128.9, 127.2, 115.5, 111.3, 102.3, 56.6, 45.3.

HRMS (ESI-TOF) m/z: [M+H]$^+$ Calcd. for $C_{24}H_{31}O_4$ 383.2217; found: 383.2243.

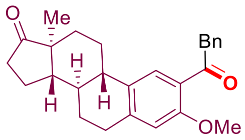

(8*R*,9*S*,13*S*,14*S*)-3-methoxy-13-methyl-2-(2-phenylacetyl)-6,7,8,9,11,12,13,14,15,16-decahydro-17*H*-cyclopenta[*a*]phenanthren-17-one (**1-18**)

37.8 mg, white solid, yield: 47 %. Eluent: pentane/ethyl acetate = 1/0 to 20/1.

^{1}H NMR (400 MHz, CDCl$_3$) δ 7.65 (s, 1H), 7.31 – 7.26 (m, 2H), 7.22 (d, *J* = 7.3 Hz, 3H), 6.67 (s, 1H), 4.29 (d, *J* = 4.7 Hz, 2H), 3.88 (s, 3H), 2.97 – 2.88 (m, 2H), 2.56 – 2.39 (m, 2H), 2.24 – 2.01 (m, 4H), 1.98 – 1.90 (m, 1H), 1.64 – 1.42 (m, 6H), 0.89 (s, 3H).

^{13}C NMR (101 MHz, CDCl$_3$) δ 220.6, 199.5, 156.7, 143.1, 135.5, 132.3, 129.7, 128.3, 128.1, 126.5, 125.6, 111.8, 55.5, 50.4, 50.1, 48.0, 43.8, 38.2, 35.8, 31.5, 29.9, 26.3, 25.8, 21.6, 13.8.

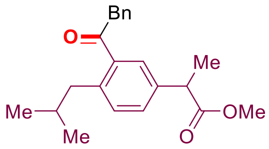

methyl 2-(4-isobutyl-3-(2-phenylacetyl)phenyl)propanoate (**1-19**)

34.5 mg, colorless oil, yield: 51 %. Eluent: pentane/ethyl acetate = 1/0 to 20/1.

^{1}H NMR (400 MHz, CDCl$_3$) δ 7.55 (d, J = 2.0 Hz, 1H), 7.34 – 7.27 (m, 3H), 7.26 – 7.20 (m, 3H), 7.15 (d, J = 7.9 Hz, 1H), 4.18 (s, 2H), 3.73 (q, J = 7.2 Hz, 1H), 3.67 (s, 3H), 2.61 (d, J = 7.2 Hz, 2H), 1.77 – 1.67 (m, 1H), 1.50 (d, J = 7.2 Hz, 3H), 0.80 (d, J = 6.6 Hz, 6H).

^{13}C NMR (101 MHz, CDCl$_3$) δ 202.1, 174.7, 140.4, 138.7, 137.8, 134.3, 132.1, 129.8, 129.7, 128.6, 127.4, 126.9, 52.2, 49.2, 44.9, 42.1, 30.0, 22.4, 18.5.

HRMS (ESI-TOF) m/z: [M+H]$^+$ Calcd. for $C_{22}H_{27}O_3$ 339.1955; found: 339.1963.

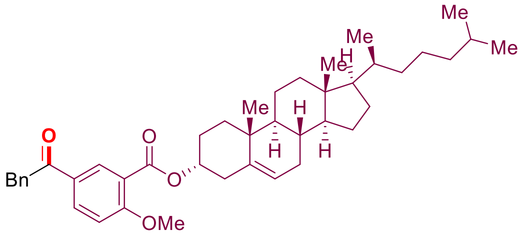

(3*R*,8*S*,9*S*,10*R*,13*R*,14*S*,17*R*)-10,13-dimethyl-17-((*S*)-6-methylheptan-2-yl)-2,3,4,7,8,9,10,11,12,13,14,15,16,17-tetradecahydro-1*H*-cyclopenta[*a*]phenanthren-3-yl 2-methoxy-5-(2-phenylacetyl)benzoate (**1-20**)

89.4 mg, white solid, yield: 70 %. Eluent: pentane/ethyl acetate = 1/0 to 20/1.

^{1}H NMR (400 MHz, CDCl$_3$) δ 8.44 (d, *J* = 2.4 Hz, 1H), 8.16 – 8.09 (m, 1H), 7.35 – 7.29 (m, 2H), 7.28 – 7.21 (m, 3H), 6.99 (d, *J* = 8.9 Hz, 1H), 5.46 – 5.38 (m, 1H), 4.92 – 4.80 (m, 1H), 4.25 (s, 2H), 3.94 (s, 3H), 2.51 – 2.43 (m, 2H), 2.07 – 1.96 (m, 3H), 1.95 – 1.89 (m, 1H), 1.88 – 1.80 (m, 1H), 1.79 – 1.70 (m, 1H), 1.61 – 1.44 (m, 6H), 1.43 – 1.27 (m, 4H), 1.26 – 1.20 (m, 2H), 1.18 – 1.08 (m, 5H), 1.07 (s, 3H), 1.05 – 0.98 (m, 3H), 0.92 (d, *J* = 6.5 Hz, 3H), 0.88 – 0.85 (m, 6H), 0.69 (s, 3H).

^{13}C NMR (101 MHz, CDCl$_3$) δ 195.7, 164.6, 162.6, 139.6, 134.6, 134.0, 132.7, 129.4, 128.8, 128.7, 126.9, 122.8, 120.7, 111.8, 74.9, 56.7, 56.3, 56.2, 50.1, 45.5, 42.3, 39.8, 39.5, 38.2, 37.1, 36.7, 36.2, 35.8, 32.0, 31.9, 28.3, 28.0, 27.9, 24.3, 23.9, 22.9, 22.6, 21.1, 19.4, 18.7, 11.9.

HRMS (ESI-TOF) m/z: [M+H]$^+$ Calcd. for $C_{43}H_{59}O_4$ 639.4408; found: 639.4411.

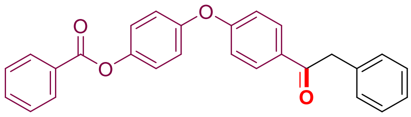

4-(4-(2-phenylacetyl)phenoxy)phenyl benzoate (**1-21**)

63.7 mg, White solid, yield: 78 %. Eluent: pentane/ethyl acetate = 1/0 to 10/1.

^{1}H NMR (400 MHz, Chloroform-*d*) δ 8.21 (d, *J* = 7.4 Hz, 2H), 8.01 (d, *J* = 8.9 Hz, 2H), 7.65 (t, *J* = 7.4 Hz, 1H), 7.52 (t, *J* = 7.7 Hz, 2H), 7.35 – 7.29 (m, 2H), 7.28 – 7.22 (m, 5H), 7.11 (d, *J* = 8.9 Hz, 2H), 7.02 (d, *J* = 8.9 Hz, 2H), 4.24 (s, 2H).

^{13}C NMR (101 MHz, $CDCl_3$) δ 196.2, 165.2, 162.0, 152.9, 147.5, 134.7, 133.8, 131.4, 131.0, 130.2, 129.4, 129.3, 128.7, 128.7, 126.9, 123.3, 121.2, 117.3, 45.5.

HRMS (ESI-TOF) m/z: $[M+H]^+$ Calcd. for $C_{27}H_{21}O_4$ 409.1434; found: 409.1435.

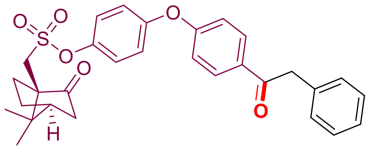

4-(4-(2-phenylacetyl)phenoxy)phenyl ((1*R*,4*S*)-7,7-dimethyl-2-oxobicyclo[2.2.1]heptan-1-yl)methanesulfonate (**1-22**)

86.0 mg, colorless oil, yield: 83 %. Eluent: pentane/ethyl acetate = 10/0 to 5/1.

^{1}H NMR (400 MHz, $CDCl_3$) δ 8.01 (d, *J* = 8.9 Hz, 2H), 7.36 – 7.29 (m, 4H), 7.29 – 7.21 (m, 3H), 7.07 (d, *J* = 9.0 Hz, 2H), 7.00 (d, *J* = 8.8 Hz, 2H), 4.24 (s, 2H), 3.84 (d, *J* = 15.0 Hz, 1H), 3.22 (d, *J* = 15.0 Hz, 1H), 2.63 – 2.49 (m, 1H), 2.48 – 2.36 (m, 1H), 2.18 – 2.03 (m, 2H), 1.98 (d, *J* = 18.5 Hz, 1H), 1.77 – 1.70 (m, 1H), 1.52 – 1.42 (m, 1H), 1.16 (s, 3H), 0.92 (s, 3H).

^{13}C NMR (101 MHz, $CDCl_3$) δ 214.1, 196.2, 161.4, 154.3, 145.3, 134.7, 131.7, 131.1, 129.4, 128.7, 126.9, 123.9, 121.2, 117.7, 58.2, 48.0, 47.7, 45.5, 42.9, 42.5, 26.9, 25.2 20.0, 19.7.

HRMS (ESI-TOF) m/z: $[M+H]^+$ Calcd. for $C_{30}H_{31}O_6S$ 519.1836; found: 519.1840.

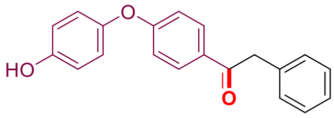

1-(4-(4-hydroxyphenoxy)phenyl)-2-phenylethan-1-one (**1-23**)

43.2 mg, White solid, yield: 71 %. Eluent: pentane/ethyl acetate = 10/1 to 5/1.

^{1}H NMR (400 MHz, $CDCl_3$) δ 7.97 (d, *J* = 8.8 Hz, 2H), 7.34 – 7.28 (m, 2H), 7.28 – 7.22 (m, 3H), 6.96 – 6.89 (m, 4H), 6.83 (d, J = 8.9 Hz, 2H), 5.81 (s, 1H), 4.24 (s, 2H).

^{13}C NMR (101 MHz, $CDCl_3$) δ 197.0, 163.2, 153.1, 148.2, 134.7, 131.1, 130.5, 129.4, 128.7, 126.9, 121.9, 116.7, 116.4, 45.4.

HRMS (ESI-TOF) m/z: $[M+H]^+$ Calcd. for $C_{20}H_{16}NaO_3$ 327.0992; found: 327.0982.

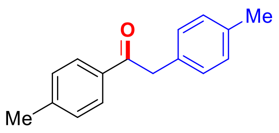

1,2-di-*p*-tolylethan-1-one (**2-1**)[9]

44.4 mg, white solid, yield: 99 %. Eluent: pentane/ethyl acetate = 1/0 to 20/1.

^{1}H NMR (400 MHz, $CDCl_3$) δ 7.91 (d, *J* = 8.2 Hz, 2H), 7.24 (d, *J* = 8.1 Hz, 2H), 7.17 – 7.10 (m, 4H), 4.21 (s, 2H), 2.39 (s, 3H), 2.31 (s, 3H).

^{13}C NMR (101 MHz, $CDCl_3$) δ 197.5, 143.9, 136.4, 134.1, 131.7, 129.4, 129.3, 129.3, 128.8, 45.1, 21.7, 21.1.

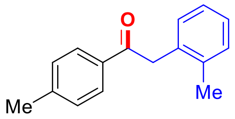

2-(*o*-tolyl)-1-(*p*-tolyl) ethan-1-one (**2-2**)[9]

41.2 mg, white solid, yield: 92 %. Eluent: pentane/ethyl acetate = 1/0 to 20/1.

^{1}H NMR (400 MHz, $CDCl_3$) δ 7.94 (d, *J* = 8.2 Hz, 2H), 7.28 (d, *J* = 8.0 Hz, 2H), 7.22 – 7.12 (m, 4H), 4.29 (s, 2H), 2.43 (s, 3H), 2.27 (s, 3H).

^{13}C NMR (101 MHz, $CDCl_3$) δ 197.2, 144.0, 136.9, 134.4, 133.7, 130.4, 130.3, 129.4, 128.5, 127.2, 126.1, 43.4, 21.7, 19.8.

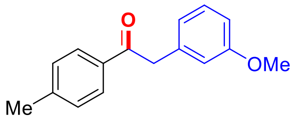

2-(3-methoxyphenyl)-1-(*p*-tolyl) ethan-1-one (**2-3**)[10]

35.1 mg, colorless oil., yield: 73 %. Eluent: pentane/ethyl acetate = 1/0 to 20/1.

^{1}H NMR (400 MHz, $CDCl_3$) δ 7.96 (d, *J* = 8.3 Hz, 2H), 7.32 – 7.26 (m, 3H), 6.90 (d, *J* = 7.8 Hz, 1H), 6.87 (t, *J* = 2.0 Hz, 1H), 6.85 – 6.81 (m, 1H), 4.27 (s, 2H), 3.83 (s, 3H), 2.45 (s, 3H).

^{13}C NMR (101 MHz, $CDCl_3$) δ 197.2, 159.8, 144.0, 136.3, 134.1, 129.6, 129.4, 128.8, 121.8, 115.1, 112.3, 55.2, 45.5, 21.7.

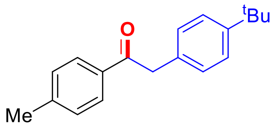

2-(4-(*tert*-butyl)phenyl)-1-(*p*-tolyl)ethan-1-one (**2-4**)

34.1 mg, colorless oil, yield: 64 %. Eluent: pentane/ethyl acetate = 1/0 to 20/1.

^{1}H NMR (400 MHz, $CDCl_3$) δ 7.94 (d, *J* = 8.3 Hz, 2H), 7.37 – 7.33 (m, 2H), 7.26 (d, *J* = 7.9 Hz, 2H), 7.21 (d, *J* = 8.2 Hz, 2H), 4.24 (s, 2H), 2.41 (s, 3H), 1.31 (s, 9H).

^{13}C NMR (101MHz, $CDCl_3$) δ 197.5, 149.6, 144.0, 134.2, 131.7, 129.3, 129.1, 128.8, 125.6, 44.9, 34.5, 31.4, 21.7.

HRMS (ESI-TOF) m/z: $[M+Na]^+$ Calcd. for $C_{19}H_{22}ONa$ 289.1563; found: 289.1567.

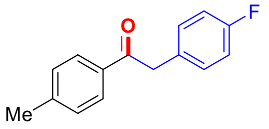

2-(4-fluorophenyl)-1-(*p*-tolyl)ethan-1-one (**2-5**)[9]

24.2 mg, white solid, yield: 53 %. Eluent: pentane/ethyl acetate = 1/0 to 20/1.

^{1}H NMR (400 MHz, CDCl$_3$) δ 7.90 (d, *J* = 8.3 Hz, 2H), 7.26 (d, *J* = 8.0 Hz, 2H), 7.24 – 7.19 (m, 2H), 7.00 (t, *J* = 8.7 Hz, 2H), 4.23 (s, 2H), 2.41 (s, 3H).

^{13}C NMR (101 MHz, CDCl$_3$) δ 197.1, 161.9 (d, *J* = 245.0 Hz), 144.2, 134.0, 131.0 (d, *J* = 8.0 Hz), 130.4 (d, *J* = 3.3 Hz), 129.4, 128.7, 115.5 (d, *J* = 21.5 Hz), 44.4, 21.7.

^{19}F NMR (376 MHz, CDCl$_3$) δ -116.2.

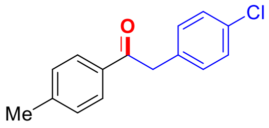

2-(4-chlorophenyl)-1-(*p*-tolyl)ethan-1-one (**2-6**)[9]

46.9 mg, white solid, yield: 96 %. Eluent: pentane/ethyl acetate = 1/0 to 20/1.

^{1}H NMR (400 MHz, CDCl$_3$) δ 7.89 (d, *J* = 8.2 Hz, 2H), 7.31 – 7.23 (m, 4H), 7.18 (d, *J* = 8.4 Hz, 2H), 4.22 (s, 2H), 2.40 (s, 3H).

^{13}C NMR (101 MHz, CDCl$_3$) δ 196.8, 144.3, 133.9, 133.2, 132.8, 130.9, 129.4, 128.8, 128.7, 44.6, 21.7.

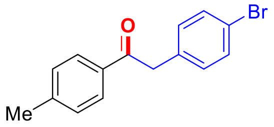

2-(4-bromophenyl)-1-(*p*-tolyl)ethan-1-one (**2-7**)[11]

53.0 mg, white solid, yield: 92 %. Eluent: pentane/ethyl acetate = 1/0 to 20/1.

^{1}H NMR (400 MHz, CDCl$_3$) δ 7.90 (d, *J* = 8.3 Hz, 2H), 7.44 (d, *J* = 8.4 Hz, 2H), 7.26 (d, *J* = 8.1 Hz, 2H), 7.14 (d, *J* = 8.4 Hz, 2H), 4.21 (s, 2H), 2.41 (s, 3H).

^{13}C NMR (101 MHz, CDCl$_3$) δ 196.7, 144.3, 133.9, 133.7, 131.7, 131.3, 129.4, 128.7, 120.9, 44.7, 21.7.

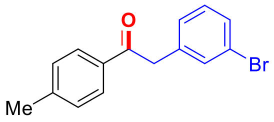

2-(3-bromophenyl)-1-(*p*-tolyl)ethan-1-one (**2-8**)[12]

57.0 mg, white solid, yield: 99 %. Eluent: pentane/ethyl acetate = 1/0 to 20/1.

^{1}H NMR (400 MHz, CDCl$_3$) δ 7.89 (d, *J* = 8.3 Hz, 2H), 7.41 (s, 1H), 7.40 – 7.34 (m, 1H), 7.26 (d, *J* = 8.0 Hz, 2H), 7.18 (d, *J* = 5.1 Hz, 2H), 4.21 (s, 2H), 2.40 (s, 3H).

^{13}C NMR (101 MHz, CDCl$_3$) δ 196.5, 144.3, 137.0, 133.9, 132.6, 130.1, 130.0, 129.5, 128.7, 128.2, 122.6, 44.8, 21.7.

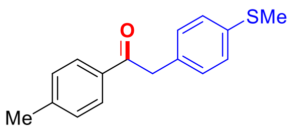

2-(4-(methylthio)phenyl)-1-(*p*-tolyl)ethan-1-one (**2-9**)

23.0 mg, white solid, yield: 45 %. Eluent: pentane/ethyl acetate = 1/0 to 20/1.

^{1}H NMR (400 MHz, CDCl$_3$) δ 7.90 (d, *J* = 8.2 Hz, 2H), 7.27 – 7.23 (m, 3H), 7.21 – 7.17 (m, 3H), 4.22 (s, 2H), 2.46 (s, 3H), 2.40 (s, 3H).

^{13}C NMR (101 MHz, CDCl$_3$) δ 197.2, 144.1, 136.8, 134.0, 131.6, 129.9, 129.4, 128.8, 127.0, 44.9, 21.7, 16.0.

HRMS (ESI-TOF) m/z: $[M+H]^+$ Calcd. for $C_{16}H_{17}OS$ 257.0995; found: 257.0997.

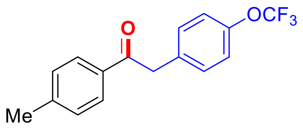

1-(*p*-tolyl)-2-(4-(trifluoromethoxy)phenyl)ethan-1-one (**2-10**)

35.9 mg, colorless oil, yield: 61 %. Eluent: pentane/ethyl acetate = 1/0 to 20/1.

^{1}H NMR (400 MHz, CDCl$_3$) δ 7.91 (d, *J* = 8.3 Hz, 2H), 7.29 – 7.25 (m, 4H), 7.17 (d, *J* = 8.8 Hz, 2H), 4.26 (s, 2H), 2.41 (s, 3H).

^{13}C NMR (101 MHz, CDCl$_3$) δ 196.7, 148.1, 144.4, 133.9, 133.4, 130.9, 129.5, 128.7, 121.1, 120.5 (q, *J* = 256.9 Hz), 44.4, 21.7.

^{19}F NMR (376 MHz, CDCl$_3$) δ -57.8.

HRMS (ESI-TOF) m/z: $[M+Na]^+$ Calcd. for $C_{16}H_{13}F_3O_2Na$ 317.0760; found: 317.0761.

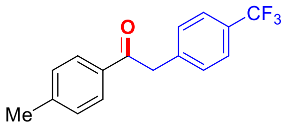

1-(*p*-tolyl)-2-(4-(trifluoromethyl)phenyl)ethan-1-one (**2-11**)

42.8 mg, white solid, yield: 77 %. Eluent: pentane/ethyl acetate = 1/0 to 20/1.

^{1}H NMR (400 MHz, CDCl$_3$) δ 7.92 (d, *J* = 8.3 Hz, 2H), 7.59 (d, *J* = 8.0 Hz, 2H), 7.38 (d, *J* = 8.0 Hz, 2H), 7.28 (d, *J* = 8.0 Hz, 2H), 4.33 (s, 2H), 2.42 (s, 3H).

^{13}C NMR (101 MHz, CDCl$_3$) δ 196.3, 144.5, 138.8, 133.8, 130.0, 129.5, 129.2 (q, *J* = 32.3 Hz), 128.7, 125.5 (q, *J* = 3.9 Hz), 124.2 (q, *J* = 273.8 Hz), 45.0, 21.7.

^{19}F NMR (376 MHz, CDCl$_3$) δ -62.5.

HRMS (ESI-TOF) m/z: $[M+H]^+$ Calcd. for $C_{16}H_{14}F_3O$ 279.0991; found: 279.0996.

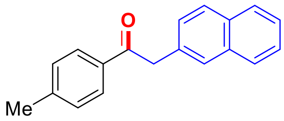

2-(naphthalen-2-yl)-1-(*p*-tolyl)ethan-1-one (**2-12**)[9]

43.2 mg, white solid, yield: 83 %. Eluent: pentane/ethyl acetate = 1/0 to 20/1.

^{1}H NMR (400 MHz, $CDCl_3$) δ 7.95 (d, *J* = 8.3 Hz, 2H), 7.80 (t, *J* = 9.2 Hz, 3H), 7.72 (s, 1H), 7.47 – 7.39 (m, 3H), 7.25 (d, *J* = 8.0 Hz, 2H), 4.43 (s, 2H), 2.40 (s, 3H).

^{13}C NMR (101 MHz, $CDCl_3$) δ 197.4, 144.1, 134.1, 133.6, 132.4, 132.4, 129.4, 128.8, 128.3, 128.1, 127.7, 127.7, 127.6, 126.1, 125.7, 45.7, 21.7.

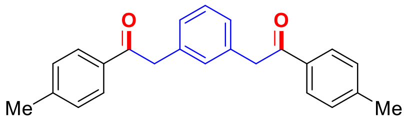

2,2'-(1,3-phenylene)bis(1-(*p*-tolyl)ethan-1-one) (**2-13**)[9]

34.9 mg, white solid, yield: 51 %. Eluent: pentane/ethyl acetate = 1/0 to 20/1.

^{1}H NMR (400 MHz, $CDCl_3$) δ 7.89 (d, *J* = 8.3 Hz, 4H), 7.26 – 7.21 (m, 5H), 7.19 – 7.10 (m, 3H), 4.23 (s, 4H), 2.40 (s, 6H).

^{13}C NMR (101 MHz, $CDCl_3$) δ 197.2, 144.0, 135.1, 134.1, 130.6, 129.3, 128.9, 128.8, 128.0, 45.3, 21.7.

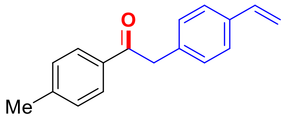

1-(*p*-tolyl)-2-(4-vinylphenyl)ethan-1-one (**2-14**)

34.9 mg, white solid, yield: 74 %. Eluent: pentane/ethyl acetate = 1/0 to 20/1.

^{1}H NMR (400 MHz, $CDCl_3$) δ 7.91 (d, *J* = 8.2 Hz, 2H), 7.36 (d, *J* = 8.1 Hz, 2H), 7.26 – 7.20 (m, 4H), 6.75 – 6.62 (m, 1H), 5.77 – 5.66 (m, 1H), 5.26 – 5.17 (m, 1H), 4.24 (s, 2H), 2.40 (s, 3H).

^{13}C NMR (101 MHz, $CDCl_3$) δ 197.2, 144.0, 136.5, 136.2, 134.4, 134.1, 129.6, 129.3, 128.8, 126.5, 113.7, 45.2, 21.7.

HRMS (ESI-TOF) m/z: $[M+H]^+$ Calcd. for $C_{17}H_{17}O$ 237.1274; found: 237.1274.

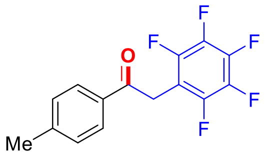

2-(perfluorophenyl)-1-(*p*-tolyl)ethan-1-one (**2-15**)[13]

54.6 mg, white solid, yield: 91 %. Eluent: pentane/ethyl acetate = 1/0 to 20/1.

^{1}H NMR (400 MHz, $CDCl_3$) δ 7.94 (d, *J* = 8.0 Hz, 2H), 7.32 (d, *J* = 8.0 Hz, 2H), 4.38 (s, 2H), 2.45 (s, 3H).

^{13}C NMR (101 MHz, $CDCl_3$) δ 192.5, 146.6, 145.0, 144.1, 141.7, 138.7, 136.2, 133.2, 129.6, 128.4, 108.7 (t, *J* = 20.7 Hz), 32.4, 21.7.

^{19}F NMR (376 MHz, $CDCl_3$) δ -142.2 – -142.5 (m), -155.7 (t, J = 20.8 Hz), -162.6 – -162.8 (m).

6. References

[1] a) Wang, M. N.; Zhang, X. M.; Ma, M. T.; Zhao, B. L., Palladium-Catalyzed Synthesis of Esters from Arenes through C−H Thianthrenation. *Org. Lett.* **2022**, *24*, 6031-6036; b) Sun, Y.-Y.; Yi, J.; Lu, X.; Zhang, Z.-Q.; Xiao, B.; Fu, Y., Cu-Catalyzed Suzuki–Miyaura reactions of primary and secondary benzyl halides with arylboronates. *Chem.Commun.* **2014**, *50*, 11060-11062.

[2] Wu, X.-F.; Schranck, J.; Neumann, H.; Beller, M., Palladium-Catalyzed Carbonylative Negishi-type Coupling of Aryl Iodides with Benzyl Chlorides. *Chem. Asian J.* **2012**, *7*, 40-44.

[3] Shu, B.; Wang, X.-T.; Shen, Z.-X.; Che, T.; Zhong, M.; Song, J.-L.; Kang, H.-J.; Xie, H.; Zhang, L. Y.; Zhang, S.-S., Iridium-catalyzed arylation of sulfoxonium ylides and arylboronic acids: a straightforward preparation of α-aryl ketones. *Org. Chem. Front.,* **2020**, *7*, 1802-1808.

[4] Yang, S.; Dai, C. Y.; Chen, Y. J.; Jiang, Y. Q. Q.; Shu, S.; Huang, Z. B.; Zhao, Y. S., Ruthenium(II)-Catalyzed Cross-Coupling of Benzoyl Formic Acids with Toluenes: Synthesis of 2-Phenylacetophenones. *Eur. J. Org. Chem.* **2021**, 2955-2961.

[5] Gu, Y. Y.; Zhang, Z.; Wang, Y.-E.; Dai, Z. T.; Yuan, Y. Q.; Xiong, D.; Li, J.; Walsh, P. J.; Mao, J. Y., Benzylic Aroylation of Toluenes Mediated by a $LiN(SiMe_3)_2/Cs^+$ System. *J. Org. Chem.* **2022**, *87*, 406-418.

[6] Mu, M. M.; Chen, L. G.; Liu, Y. L.; Fang, W. W.; Li, Y., An efficient Fe_2O_3/HY catalyst for Friedel–Crafts acylation of *m*-xylene with benzoyl chloride. *RSC Adv.*, **2014**, *4*, 36951-36958.

[7] Solomek, T.; Stacko, P.; Veetil, A. T.; Pospı´sil, T.; Klan, P., Photoenolization-Induced Oxirane Ring Opening in 2,5-Dimethylbenzoyl Oxiranes to Form Pharmaceutically Promising Indanone Derivatives. *J. Org. Chem*. **2010**, *75*, 7300-7309.

[8] Chen, R. J.; Qi, J. F.; Mao, Z. J.; Cui, S. L., Rh(III)-catalyzed C-H activation/cyclization of oximes with alkenes for regioselective synthesis of isoquinolines. *Org. Biomol. Chem*., **2016**, *14*, 6201-6204.

[9] Zhang, S.; Bedi, D.; Cheng, L.; Unruh, D. K.; Li, G. G.; Findlater, M., Cobalt(II)-Catalyzed Stereoselective Olefin Isomerization: Facile Access to Acyclic Trisubstituted Alkenes. *J. Am. Chem. Soc.* **2020**, *142*, 19, 8910-8917.

[10] Henry, M. C.; Sutherland, A., Synthesis of Benzo[*b*]furans by Intramolecular C–O Bond Formation Using Iron and Copper Catalysis. *Org. Lett.* **2020**, *22*, 2766-2770.

[11] Shen, Z.-L.; Xu, X.-P.; Ji, S. J., Brønsted Base-Catalyzed One-Pot Three-Component Biginelli-Type Reaction: An Efficient Synthesis of 4,5,6-Triaryl-3,4-dihydropyrimidin-2(1*H*)-one and Mechanistic Study. *J. Org. Chem.* **2010**, *75*, 1162-1167.

[12] Bhukta, S.; Chatterjee, R.; Dandela, R., Metal-free oxidative radical arylation of styrene with anilines to access 2-arylacetophenones and selective oxidation of amine. *J. Mol. Stru.* **2023**, *1279*, 134995.

[13] Doi, R.; Hayashi, K.; Sato, Y., Palladium-catalyzed Decarboxylative α-Polyfluoroarylation of Ketones. *Chem. Lett.* **2021**, *50*, 1181-1183.

7. Copy of ^{1}H and ^{13}C NMR Spectra of Products

^{1}H NMR (400 MHz, $CDCl_3$) 1-1

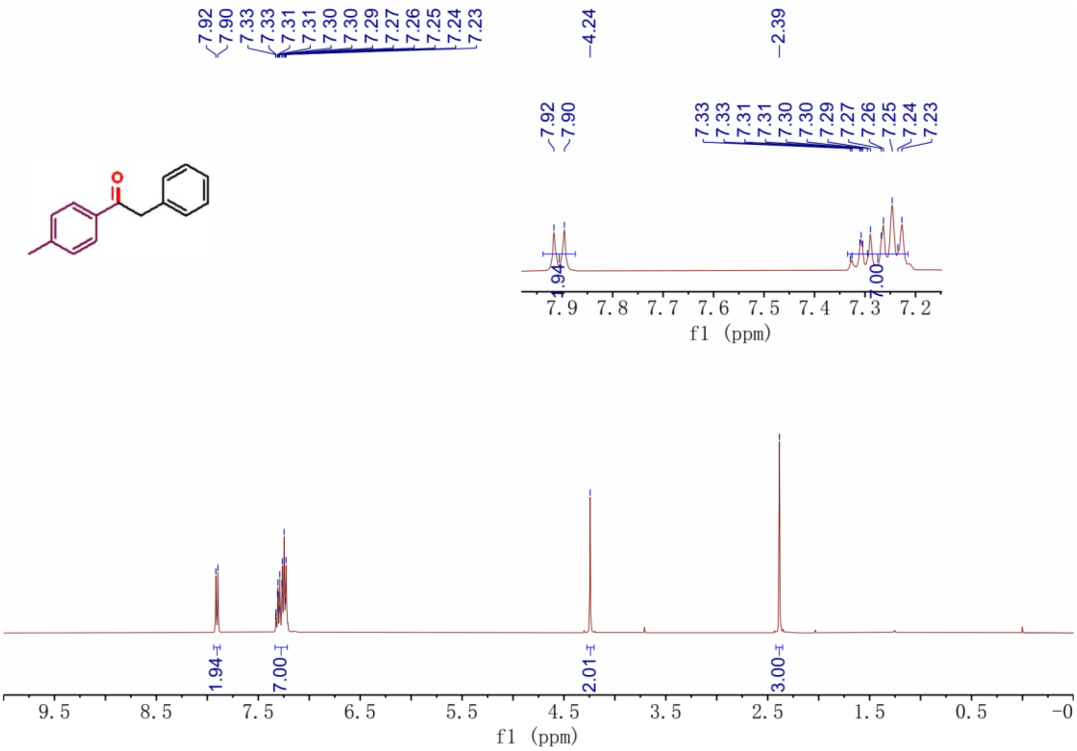

^{13}C NMR (101 MHz, $CDCl^3$) 1-1

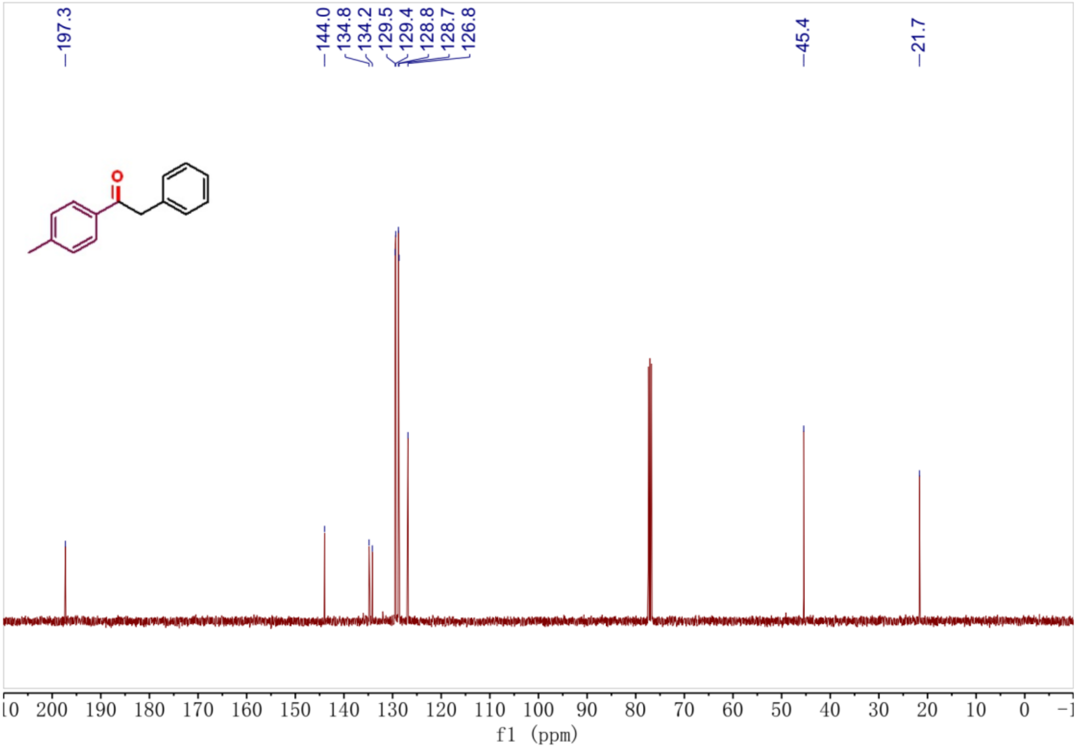

^{1}H NMR (700 MHz, $CDCl_3$) 1-2

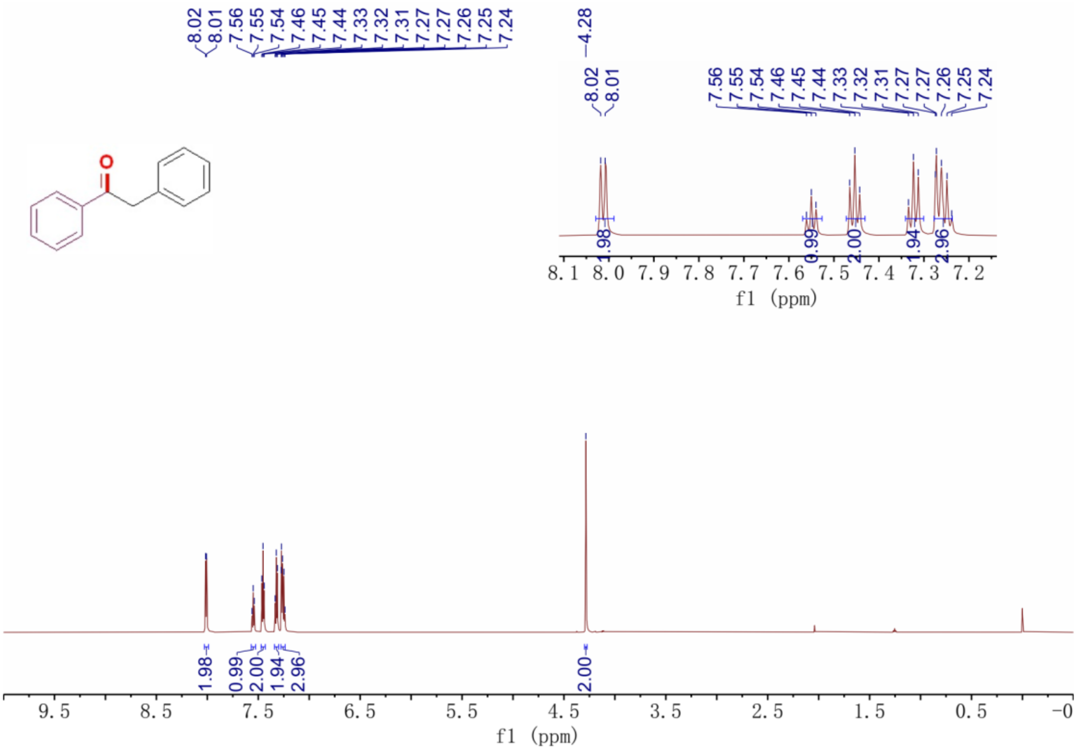

^{13}C NMR (176 MHz, $CDCl_3$) 1-2

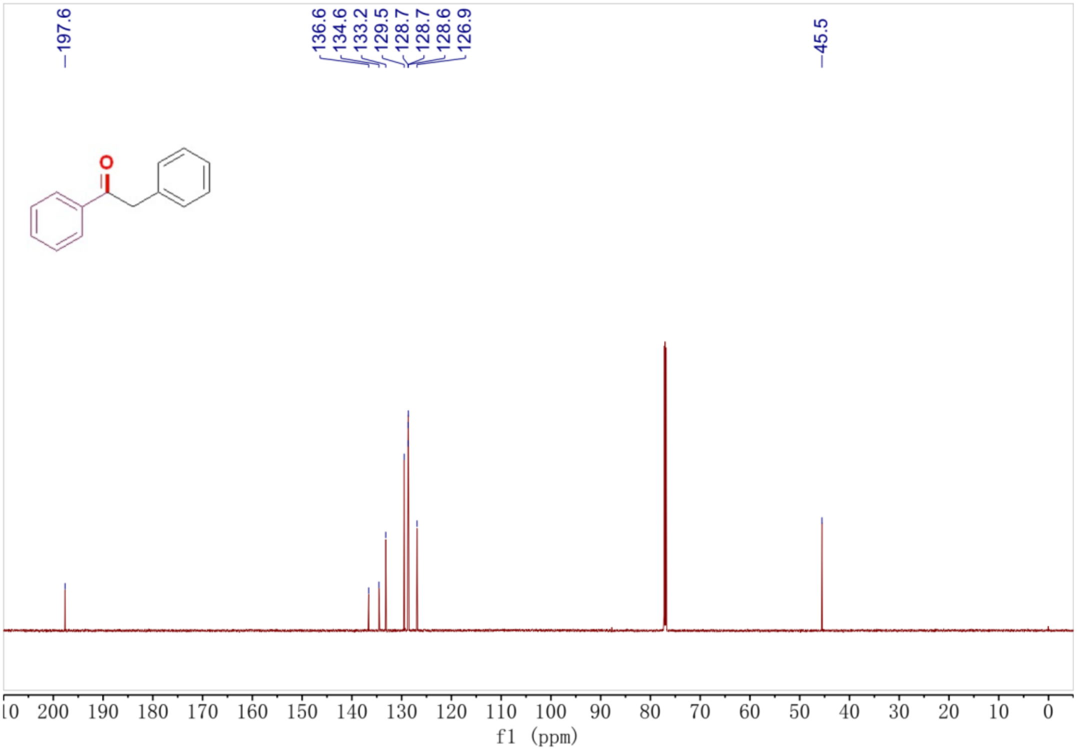

^{1}H NMR (700 MHz, $CDCl_3$) 1-3

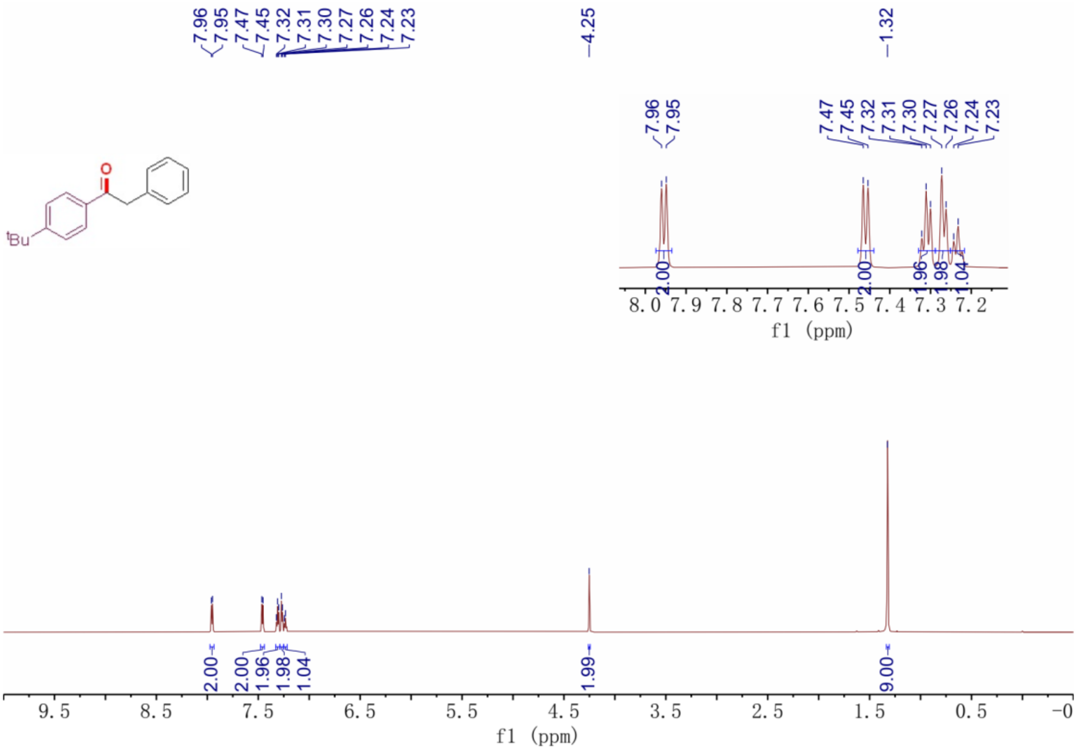

^{13}C NMR (176 MHz, $CDCl_3$) 1-3

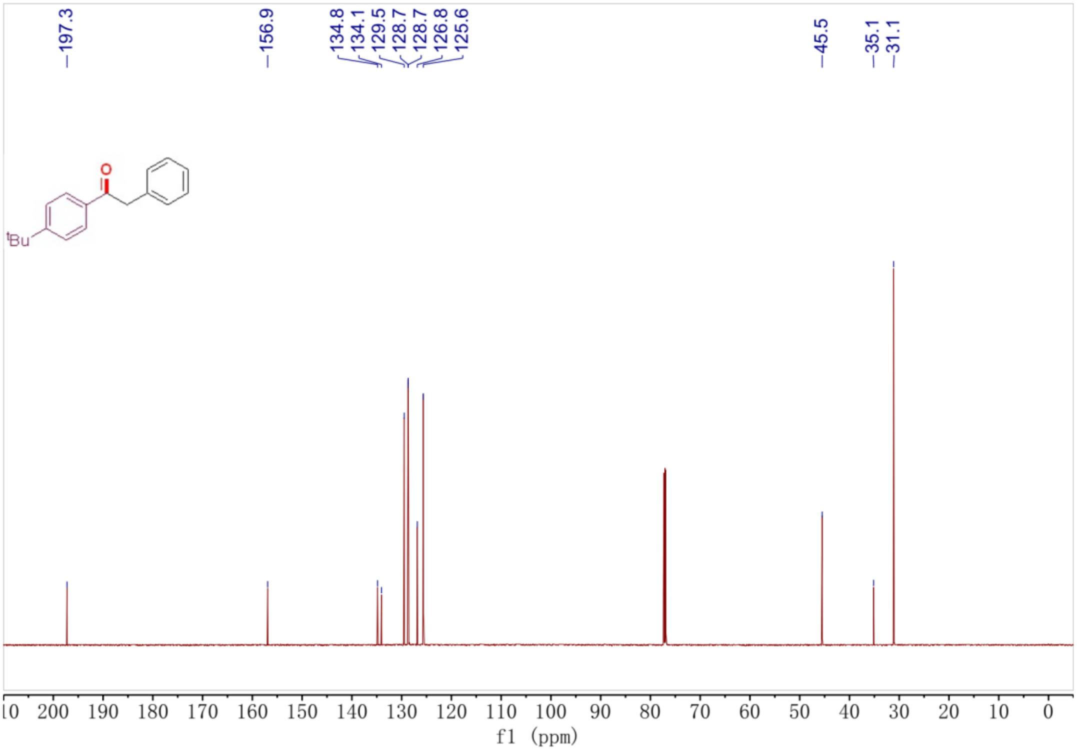

^{1}H NMR (700 MHz, $CDCl_3$) 1-4

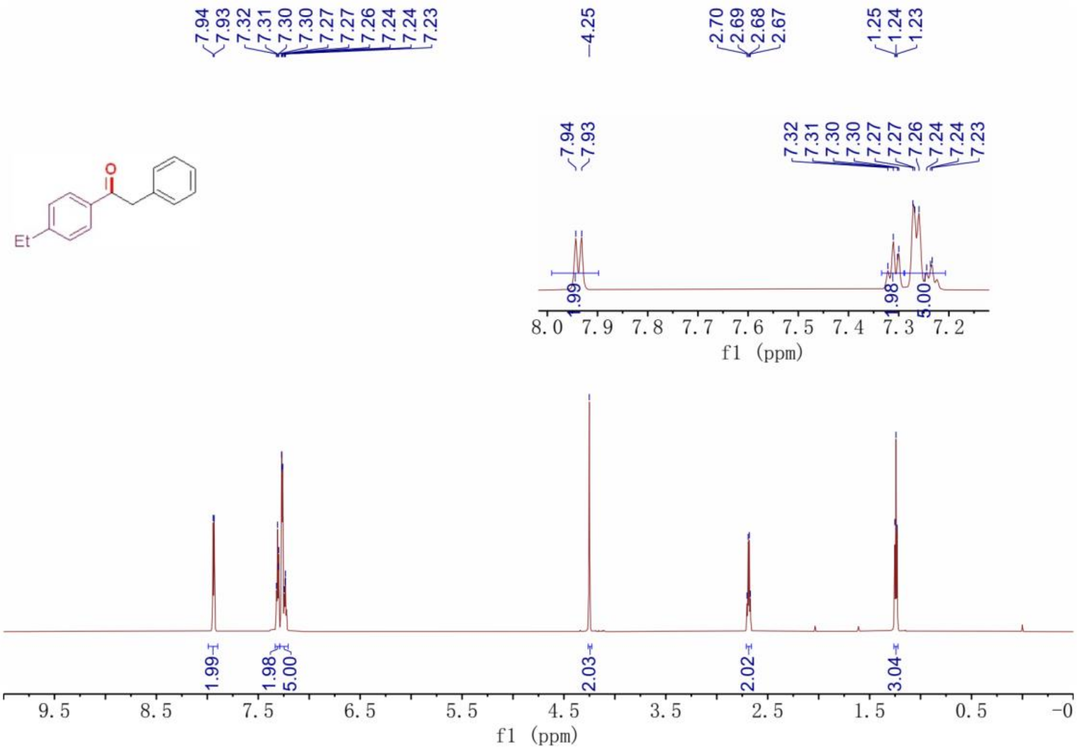

^{13}C NMR (176 MHz, $CDCl_3$) 1-4

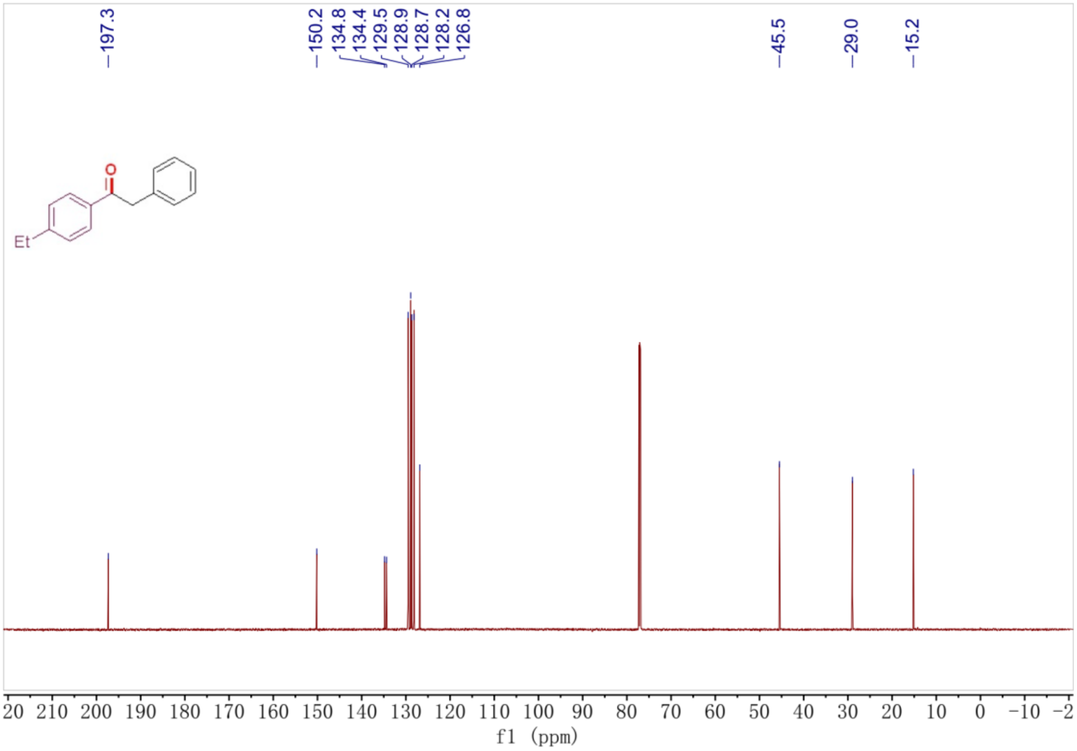

^{1}H NMR (400 MHz, $CDCl_3$) 1-5

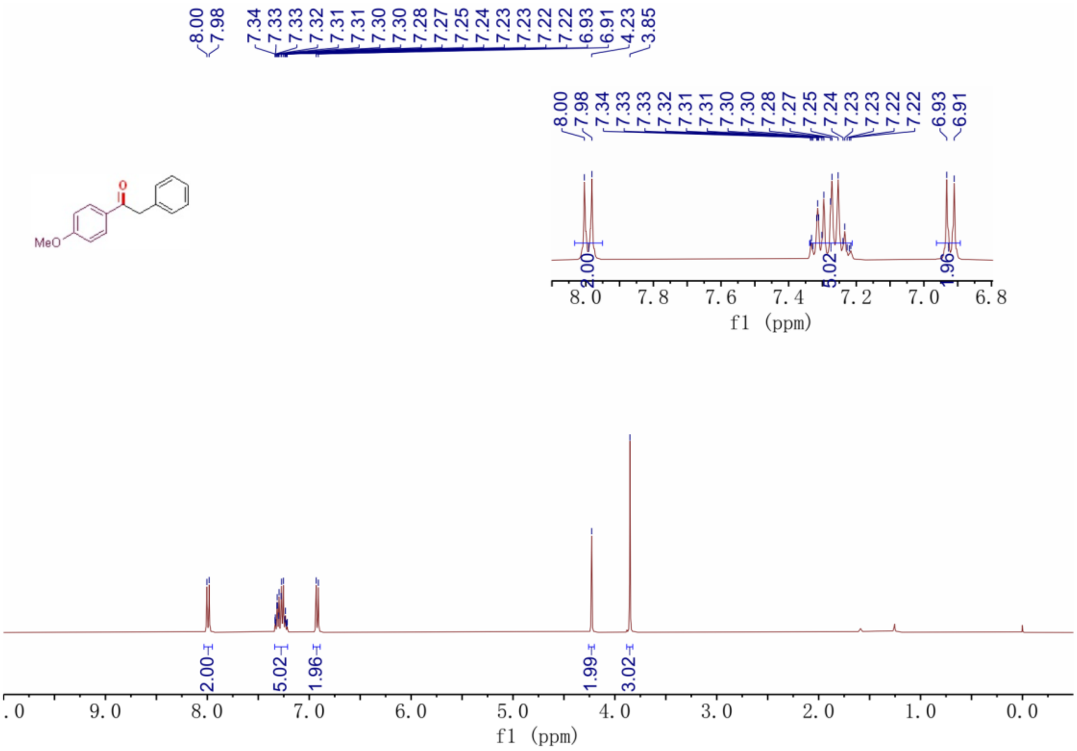

^{13}C NMR (101 MHz, $CDCl_3$) 1-5

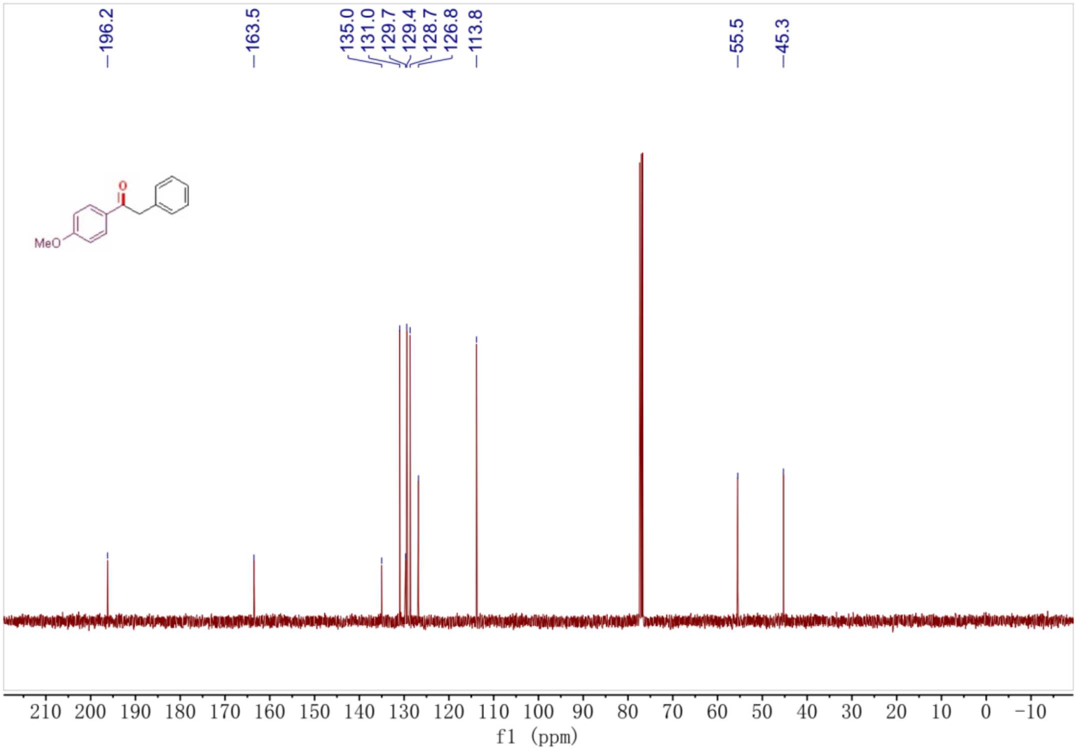

^{1}H NMR (400 MHz, $CDCl_3$) 1-6

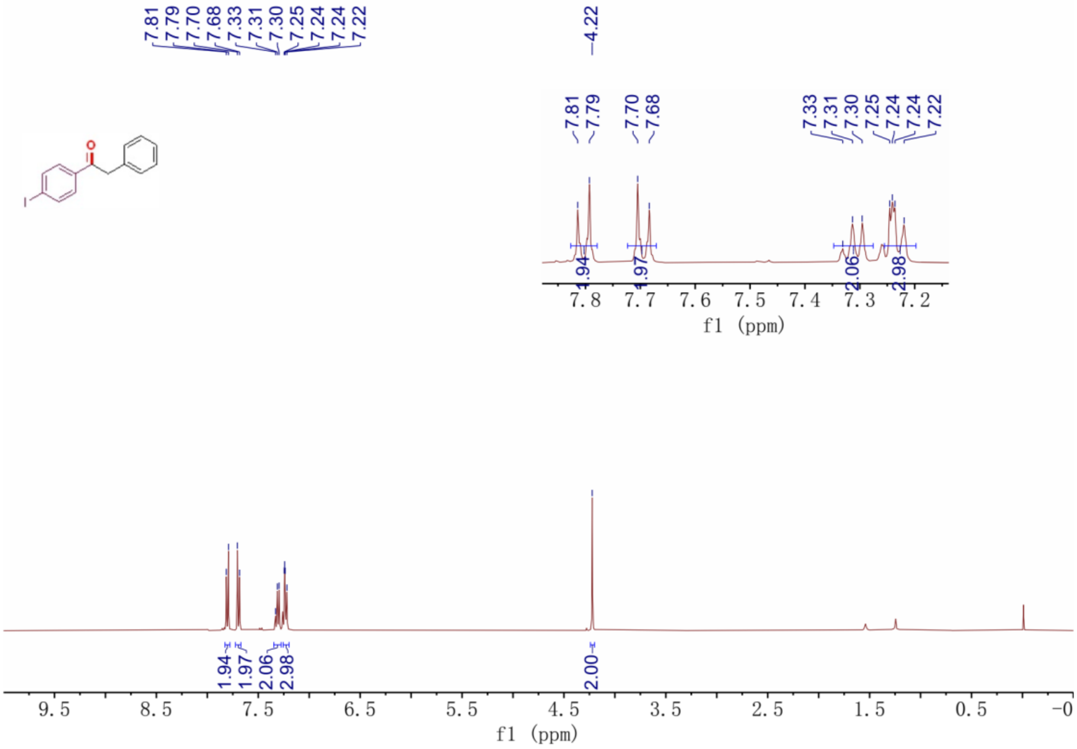

^{13}C NMR (101 MHz, $CDCl_3$) 1-6

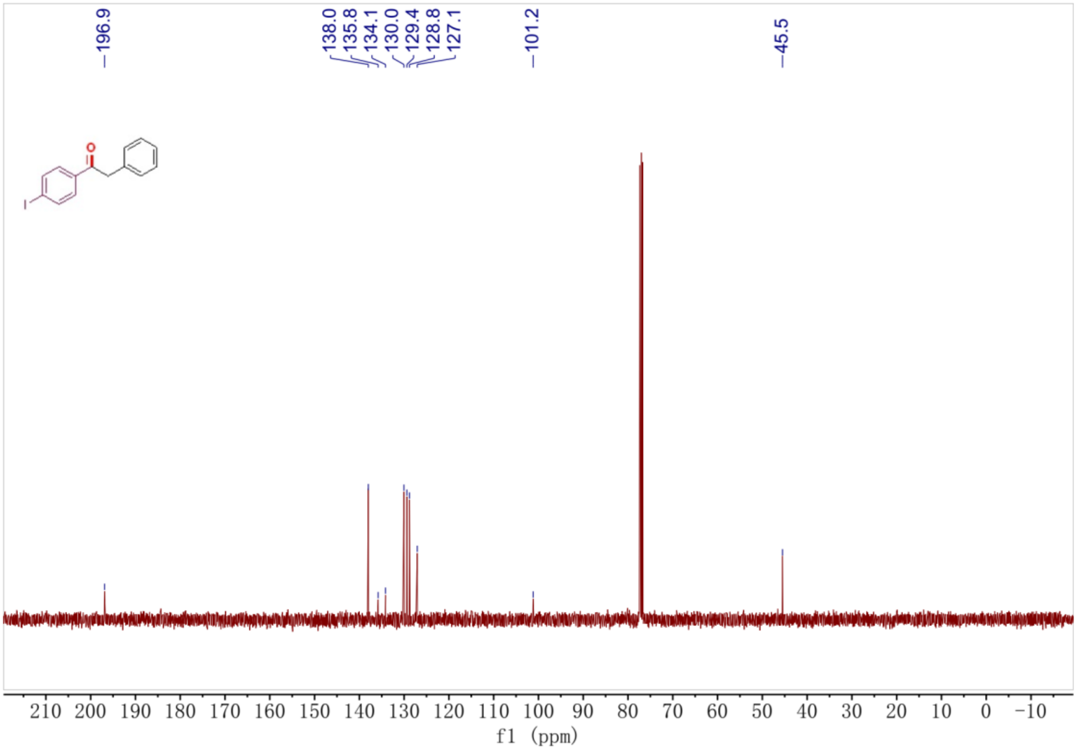

^{1}H NMR (700 MHz, $CDCl_3$) 1-7

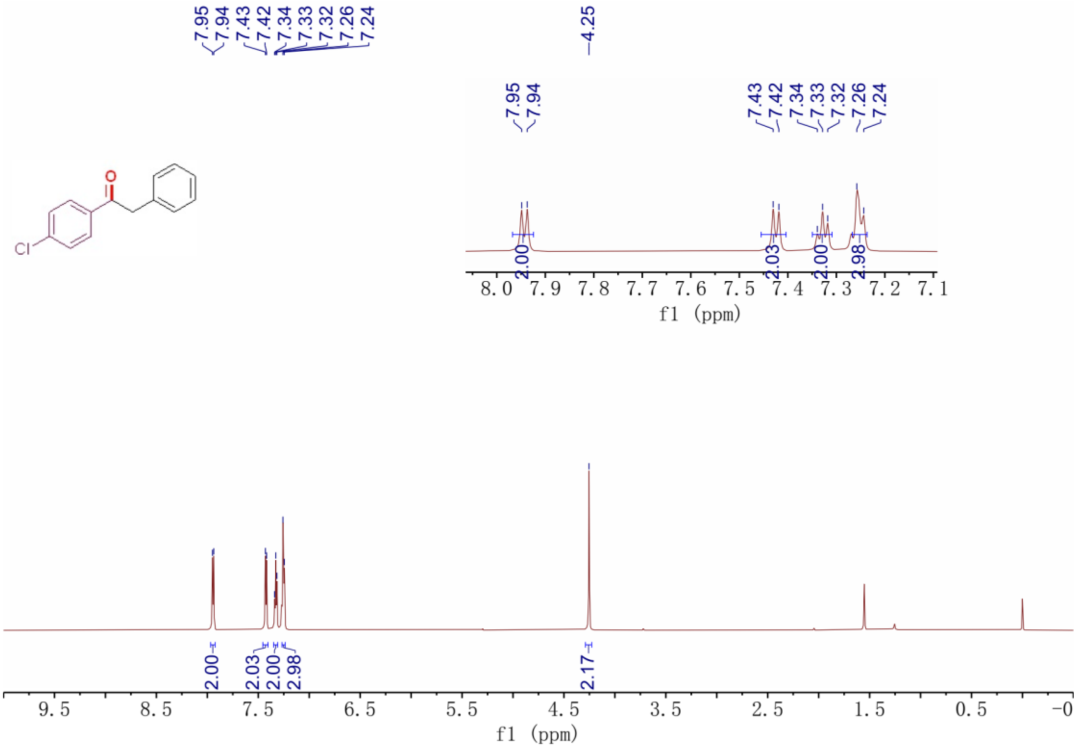

^{13}C NMR (176 MHz, $CDCl_3$) 1-7

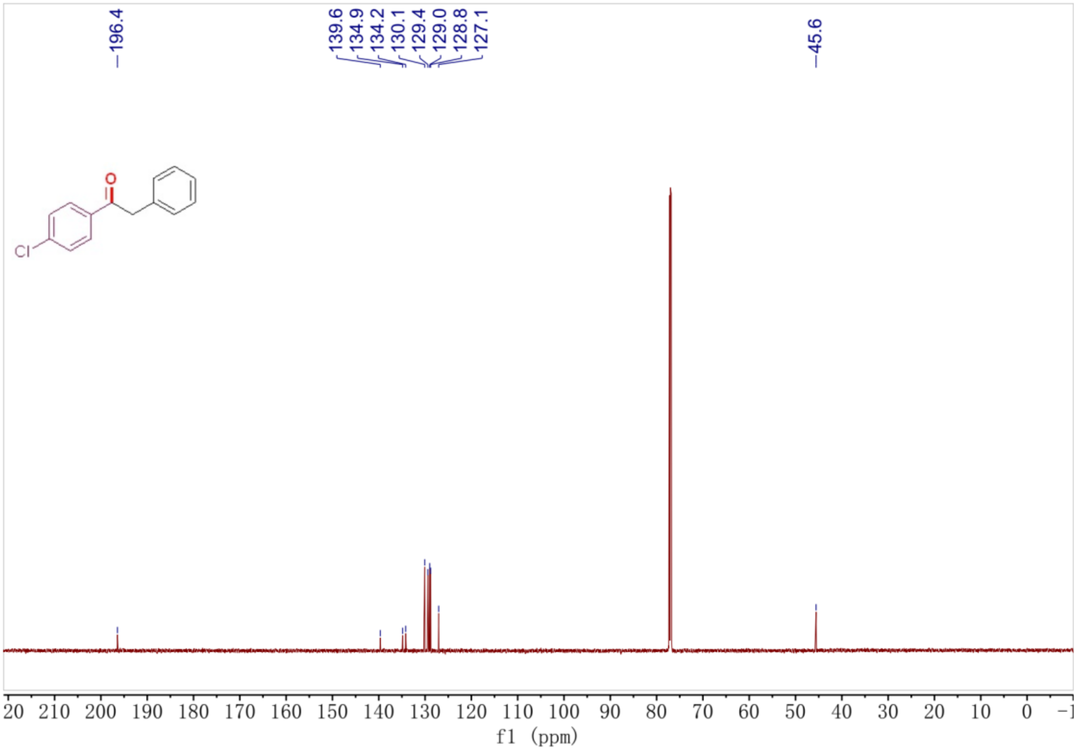

^{1}H NMR (700 MHz, $CDCl_3$) 1-8

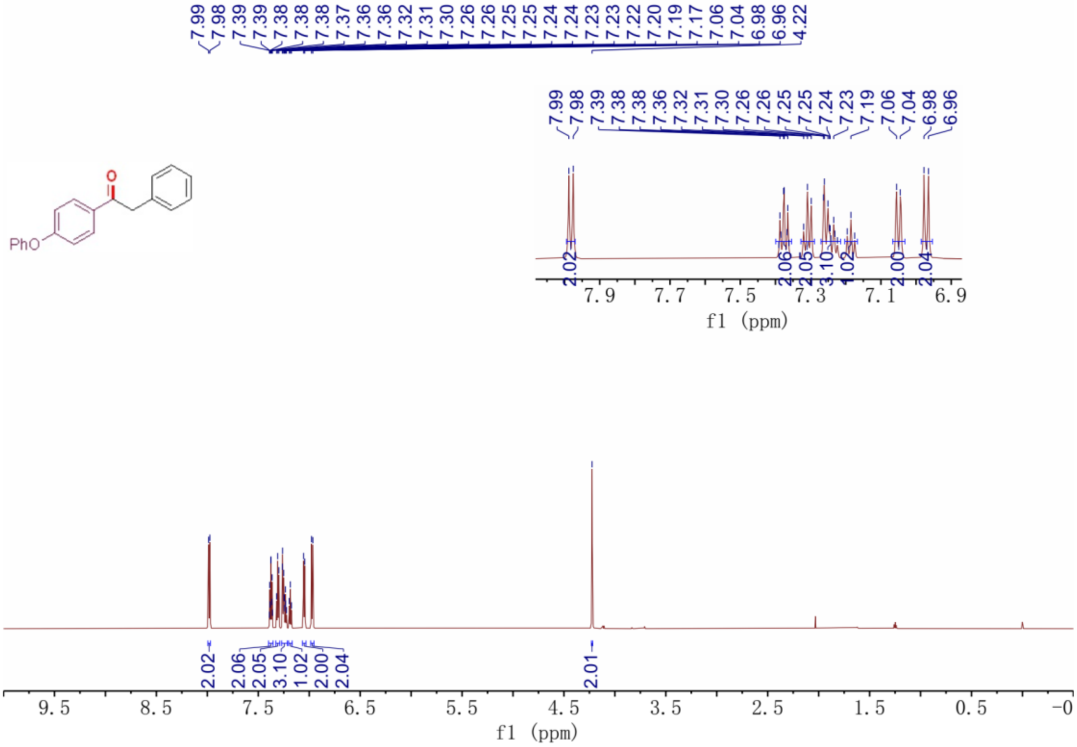

^{13}C NMR (176 MHz, $CDCl_3$) 1-8

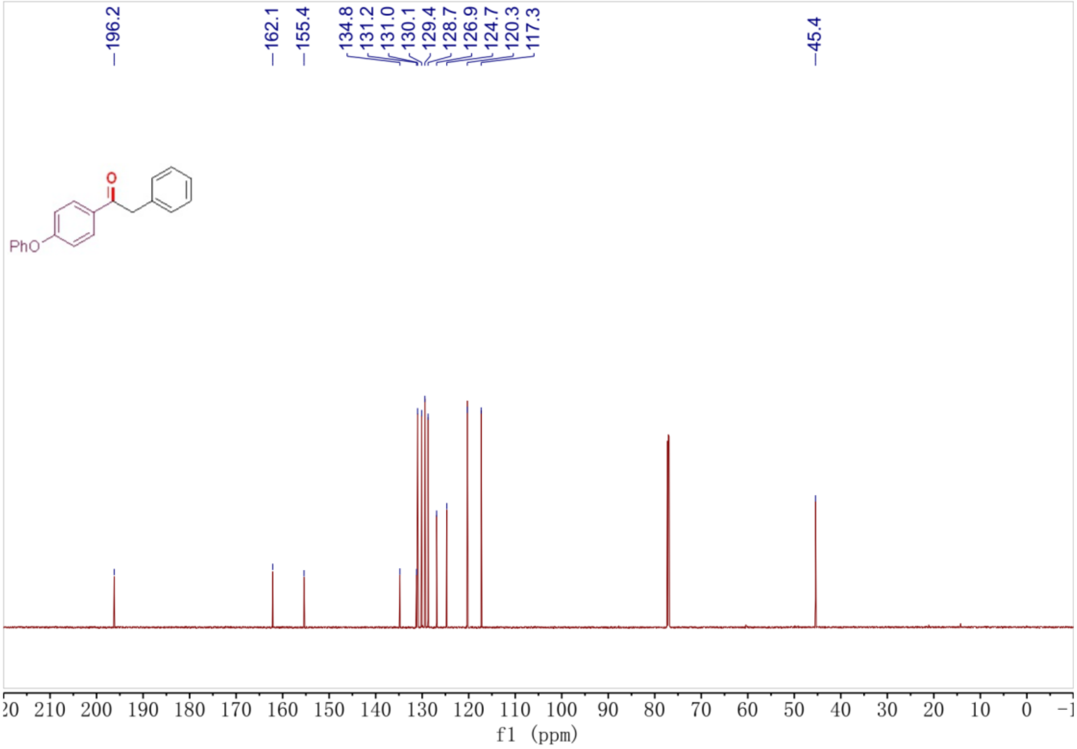

^{1}H NMR (700 MHz, $CDCl_3$) 1-9

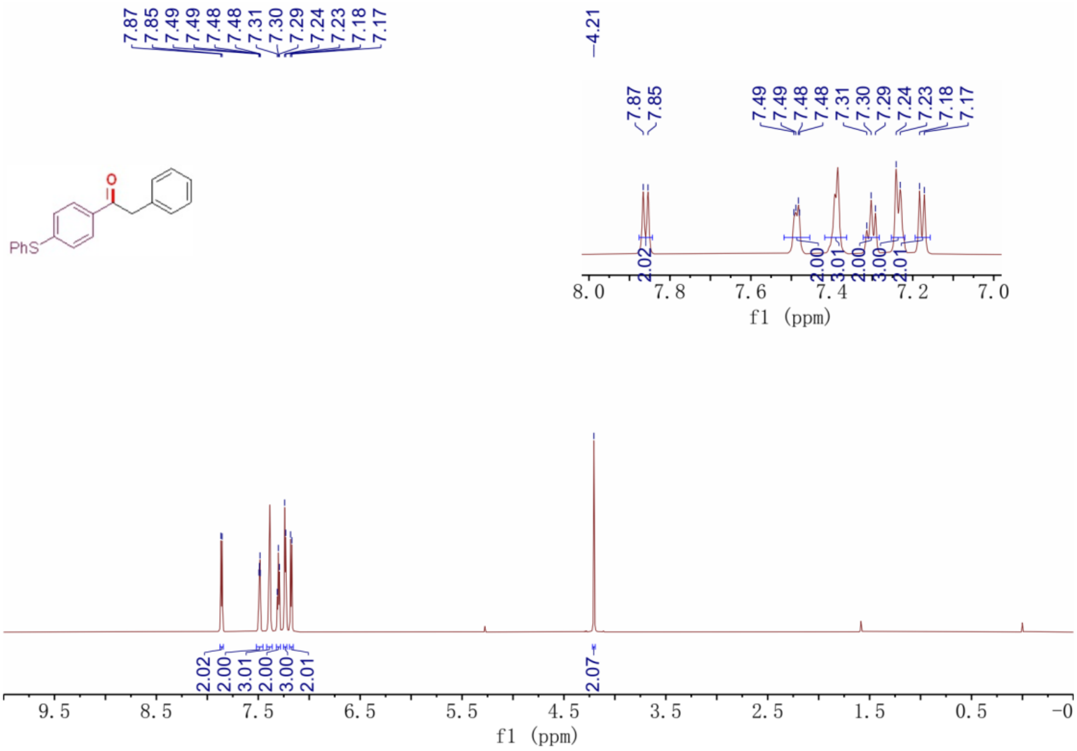

^{13}C NMR (176 MHz, $CDCl_3$) 1-9

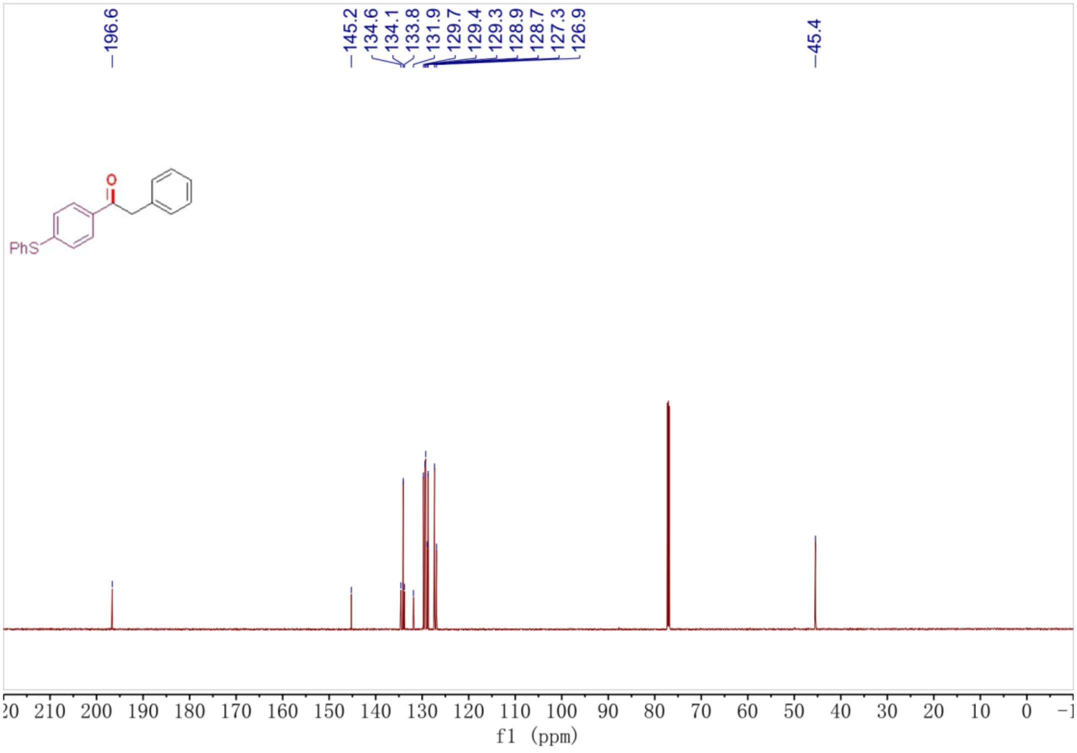

^{1}H NMR (700 MHz, CDCl$_3$) 1-10

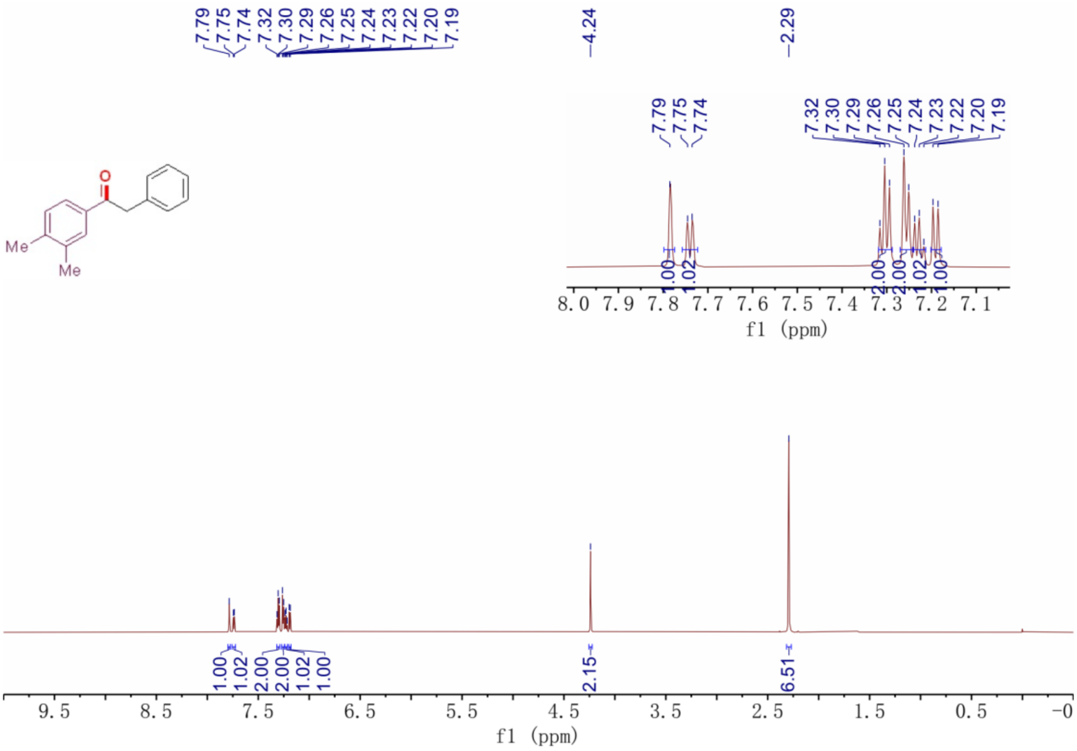

^{13}C NMR (176 MHz, CDCl$_3$) 1-10

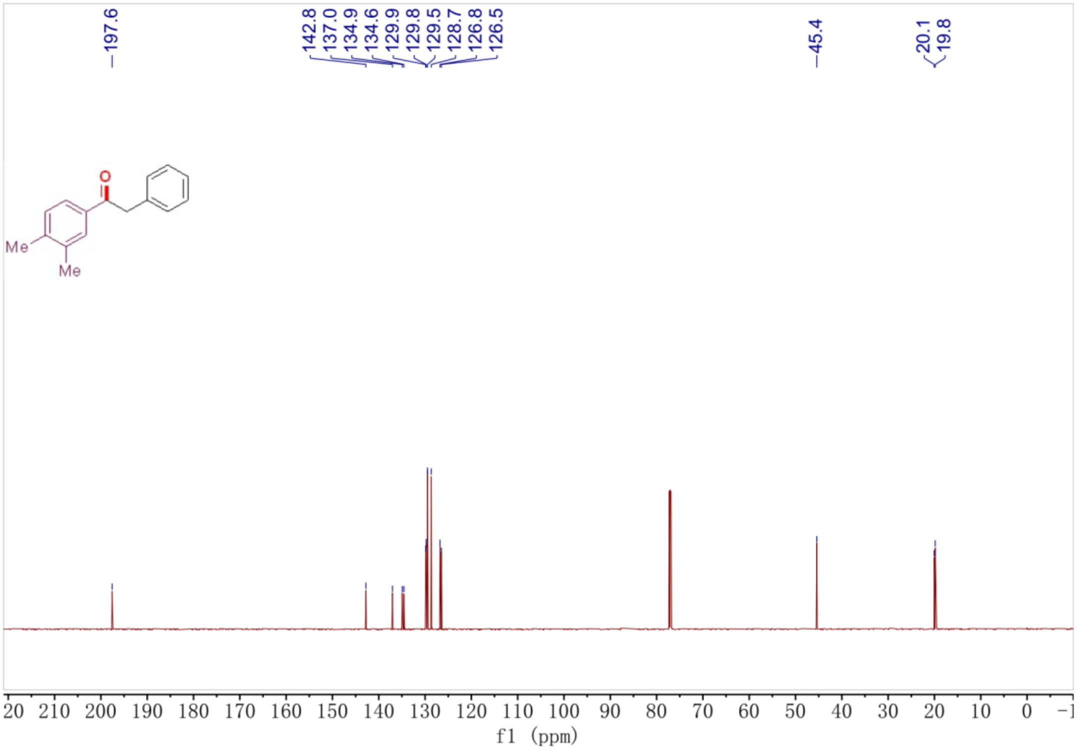

^{1}H NMR (700 MHz, $CDCl_3$) 1-11

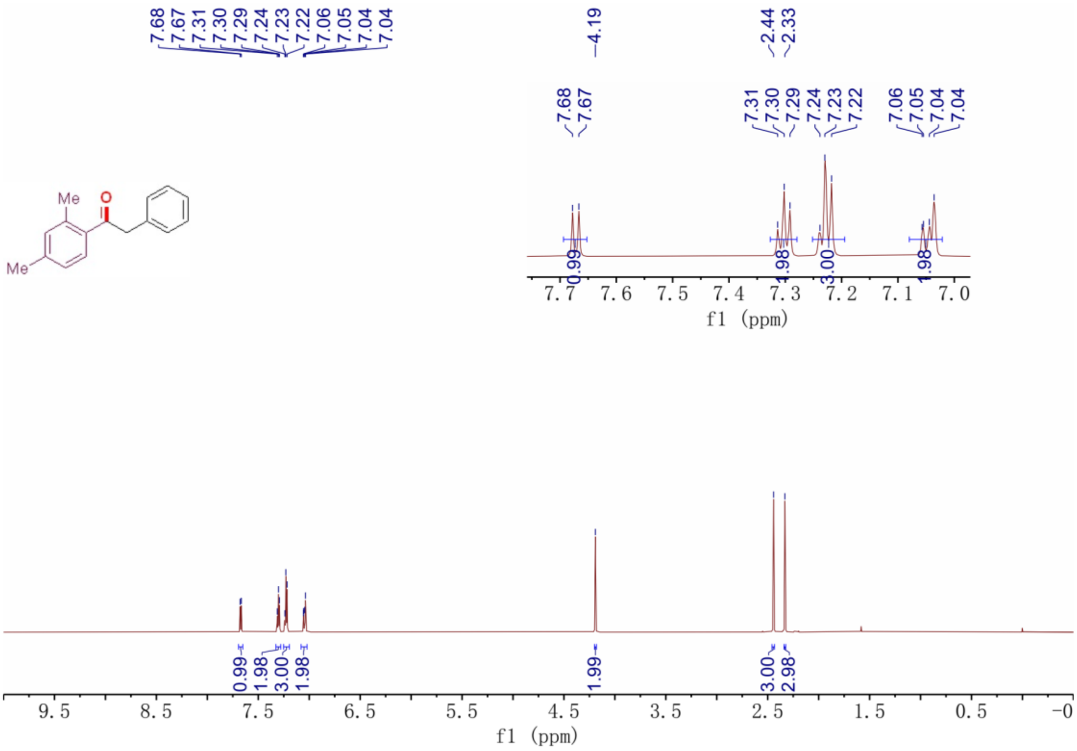

^{13}C NMR (176 MHz, $CDCl_3$) 1-11

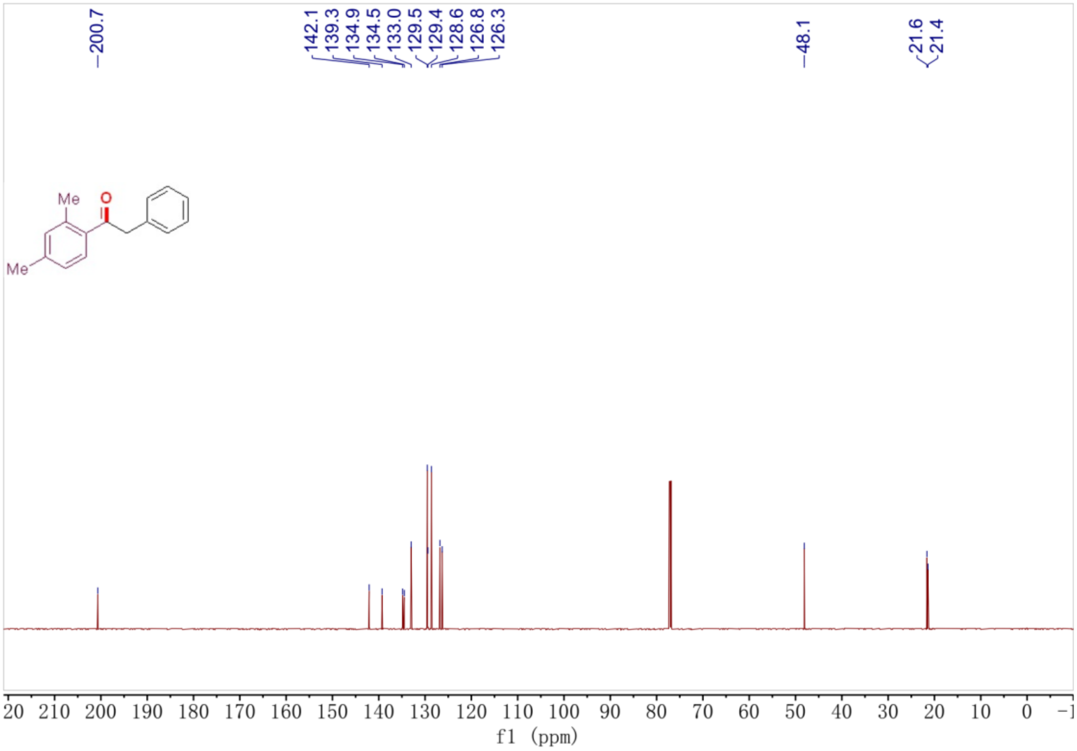

^{1}H NMR (700 MHz, $CDCl_3$) 1-12

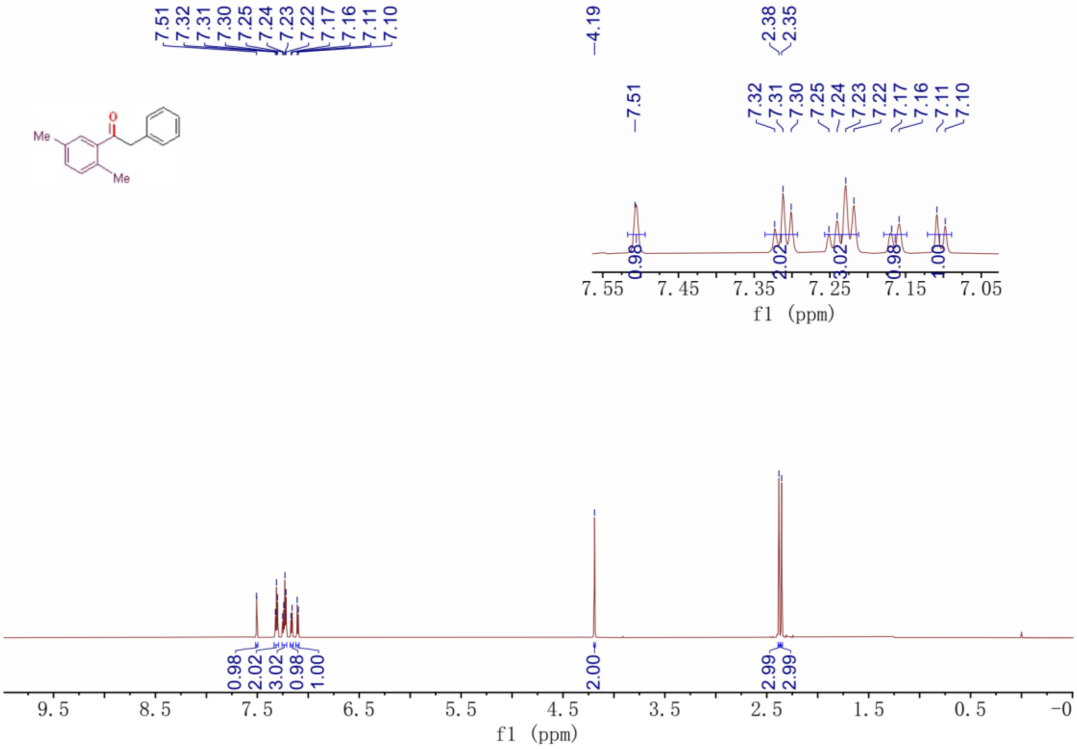

^{13}C NMR (176 MHz, $CDCl_3$) 1-12

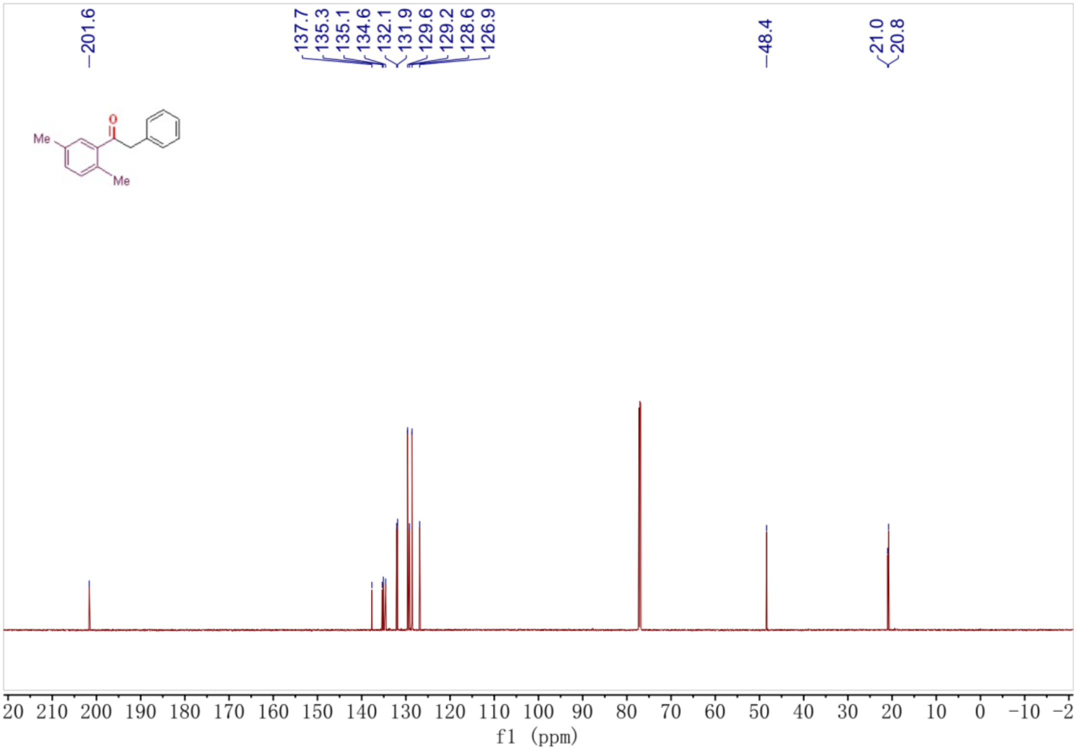

^{1}H NMR (700 MHz, $CDCl_3$) 1-13

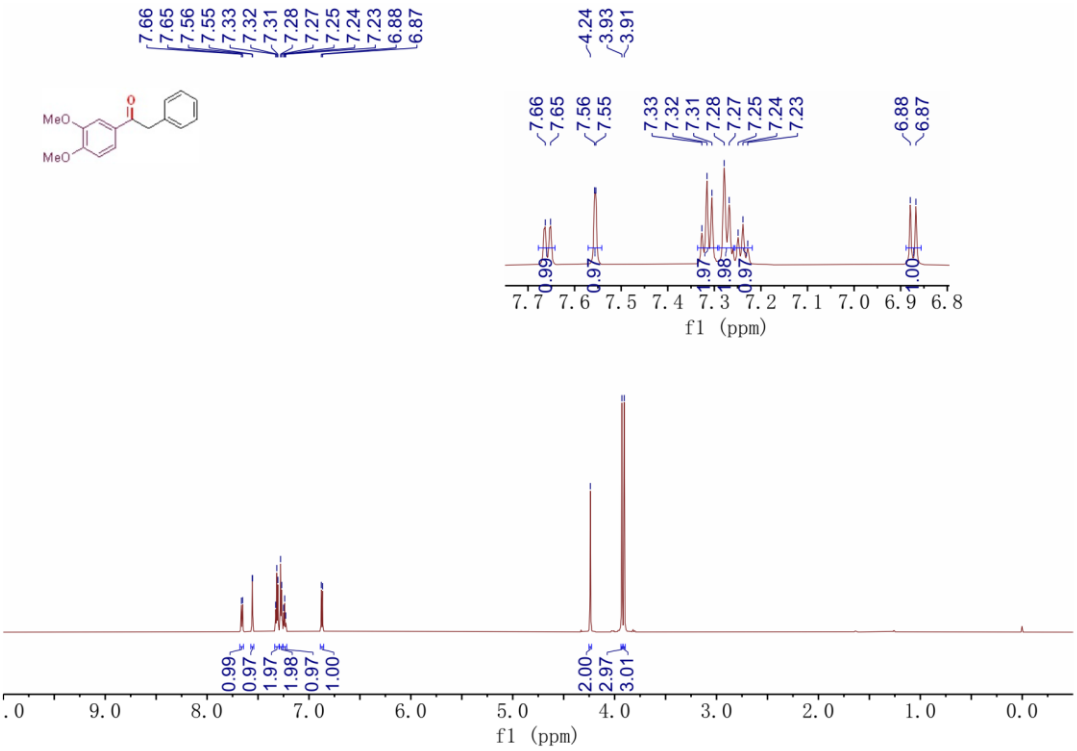

^{13}C NMR (176 MHz, $CDCl_3$) 1-13

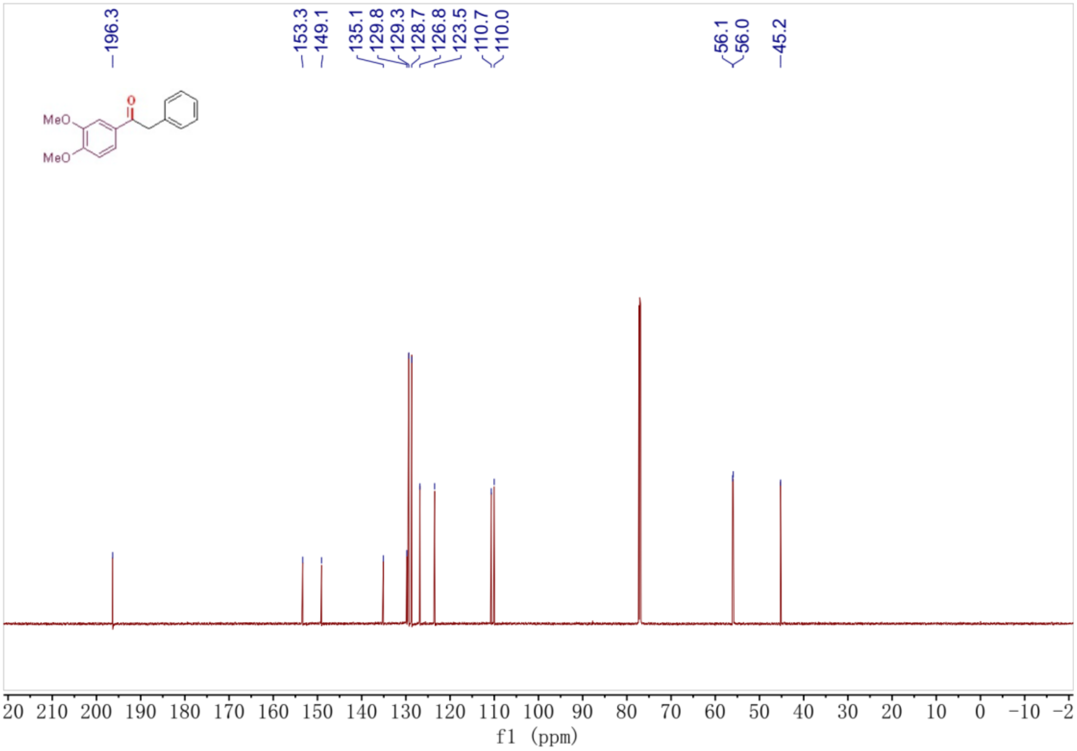

^{1}H NMR (700 MHz, $CDCl_3$) 1-14

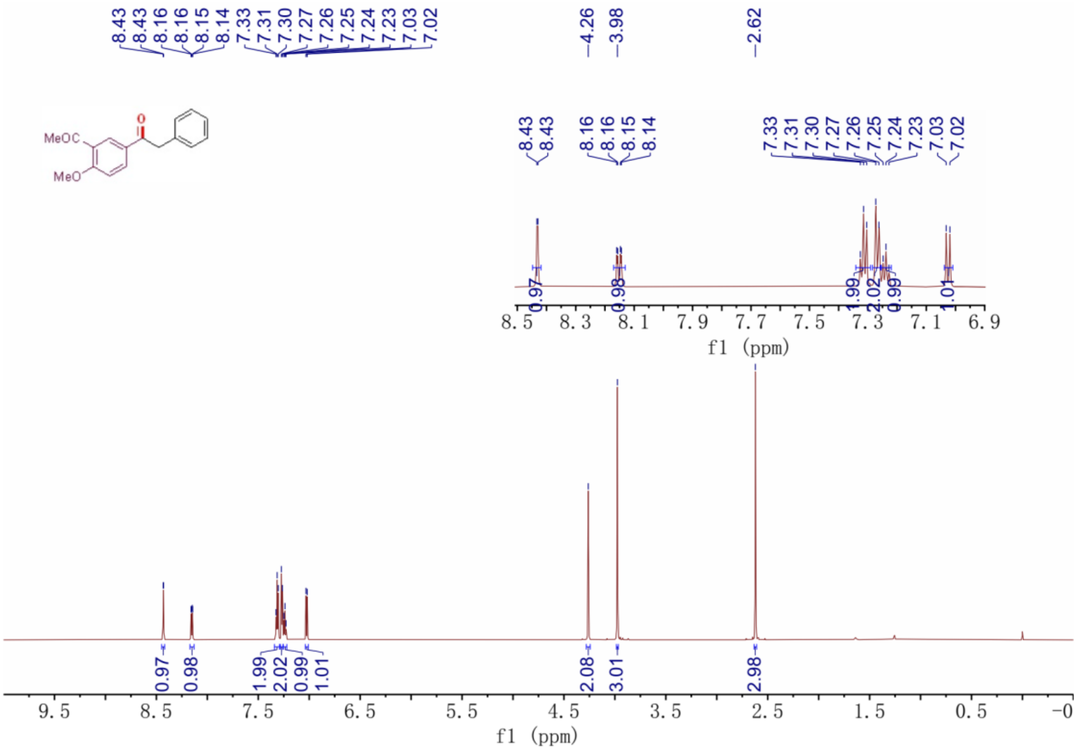

^{13}C NMR (176 MHz, $CDCl_3$) 1-14

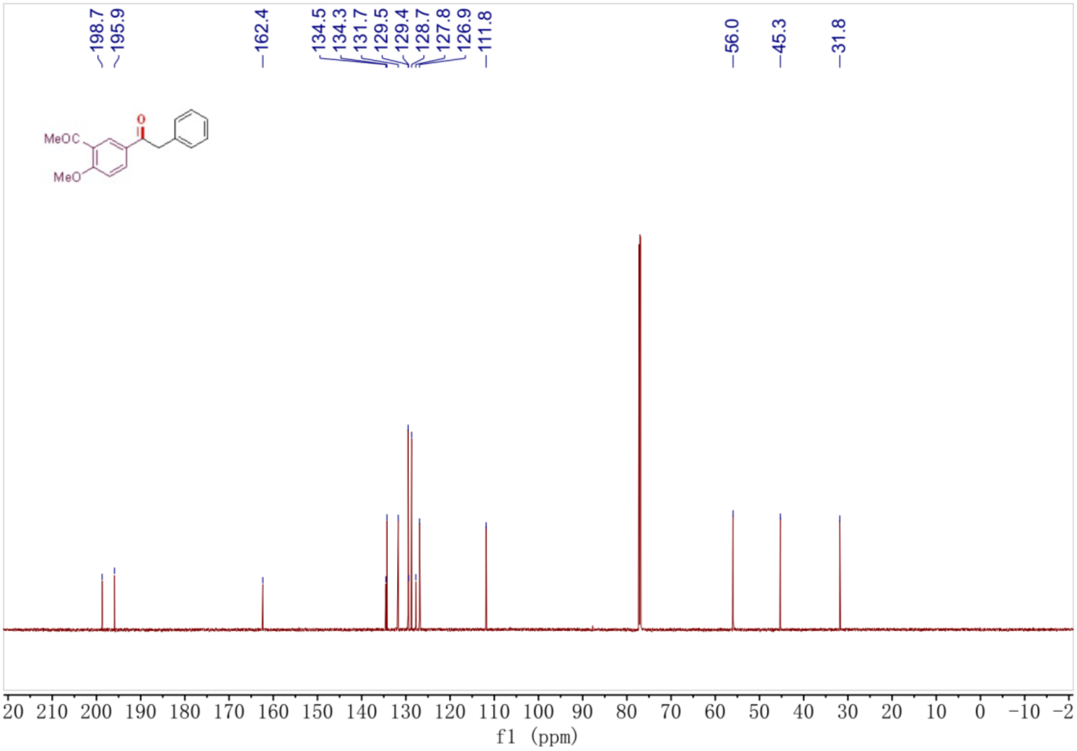

^{1}H NMR (700 MHz, $CDCl_3$) 1-15

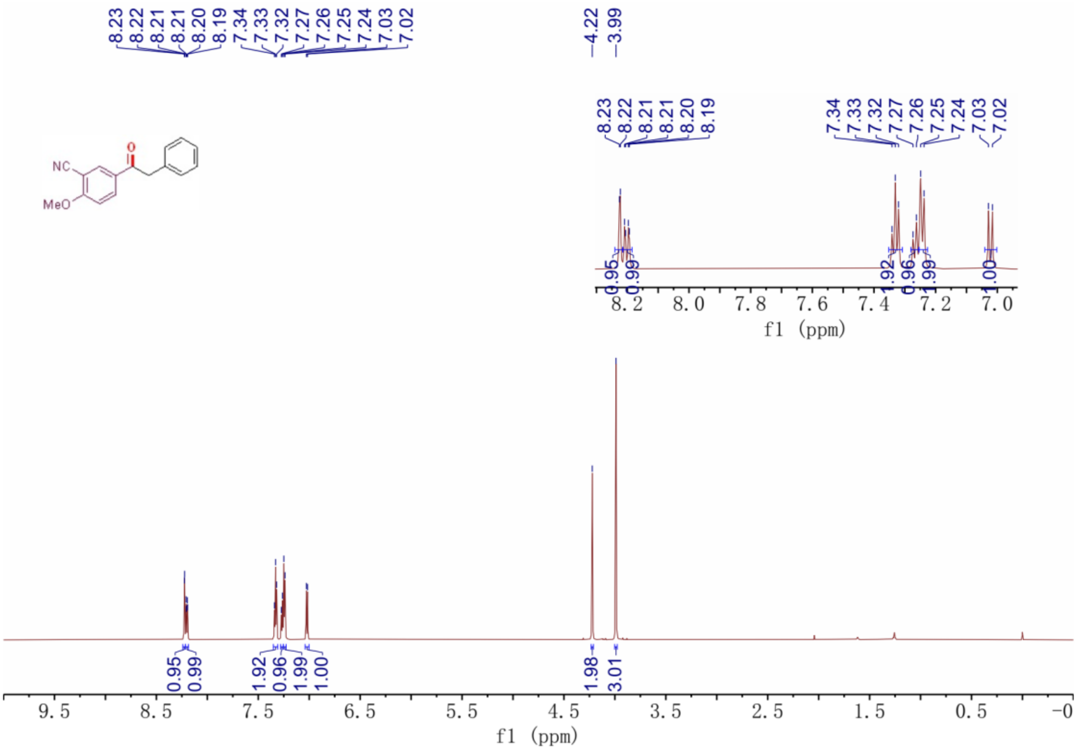

^{13}C NMR (176 MHz, $CDCl_3$) 1-15

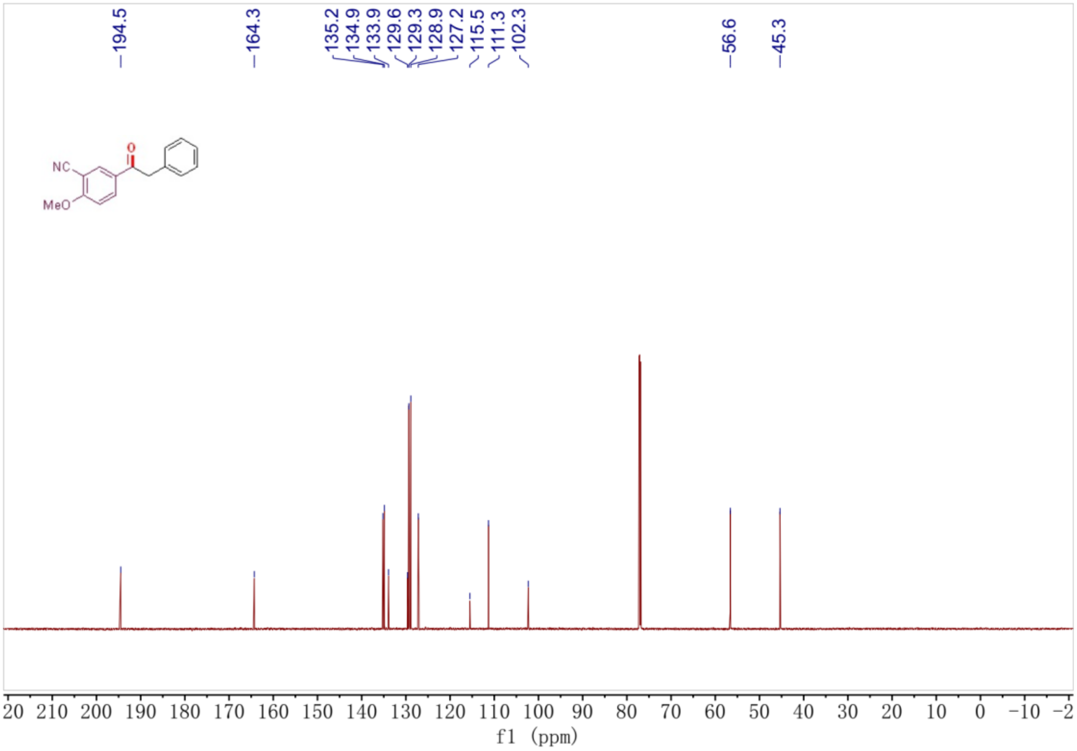

^{1}H NMR (400 MHz, $CDCl_3$) 1-16

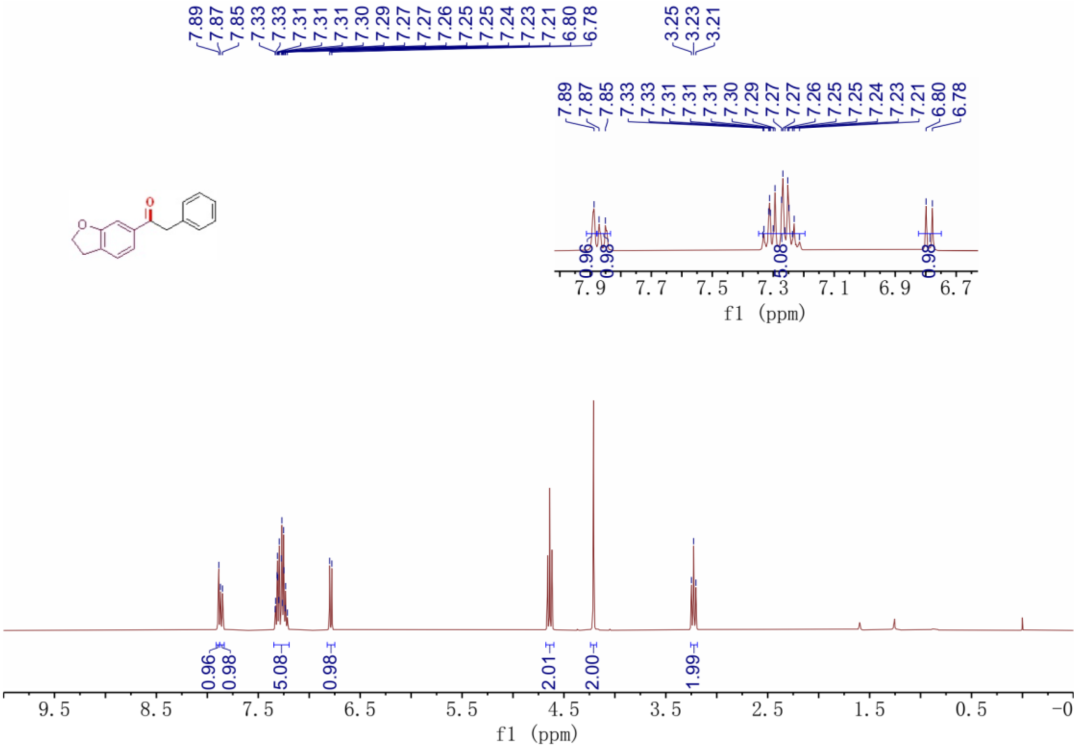

^{13}C NMR (101 MHz, $CDCl_3$) 1-16

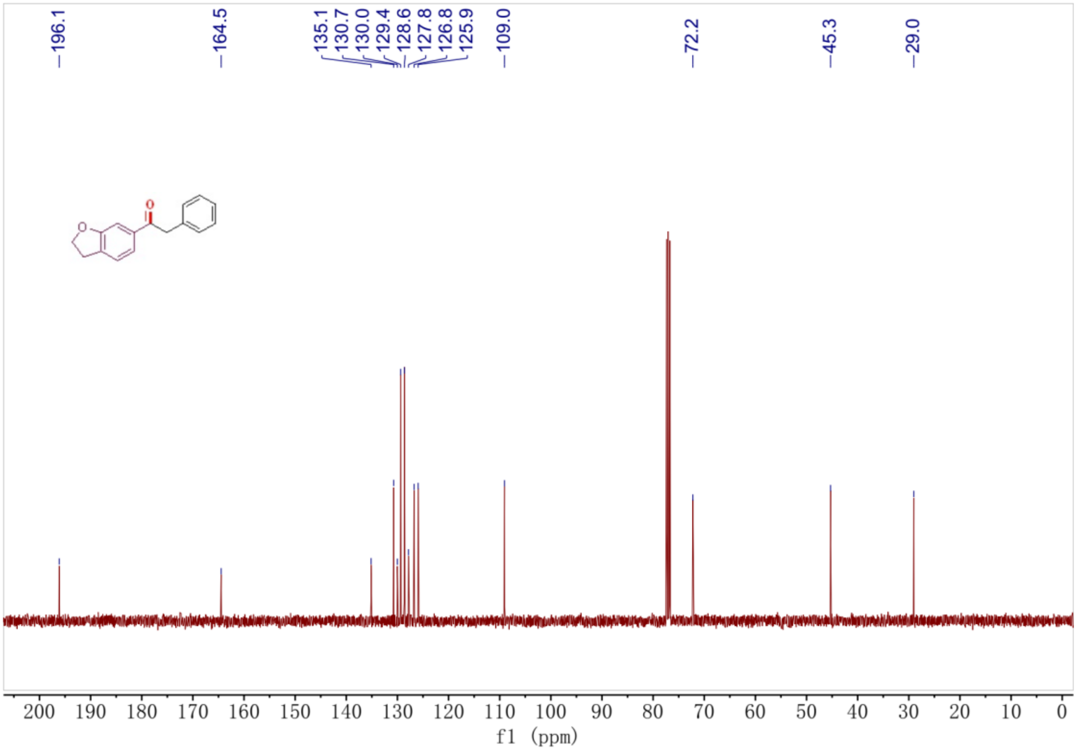

^{1}H NMR (400 MHz, $CDCl_3$) 1-17

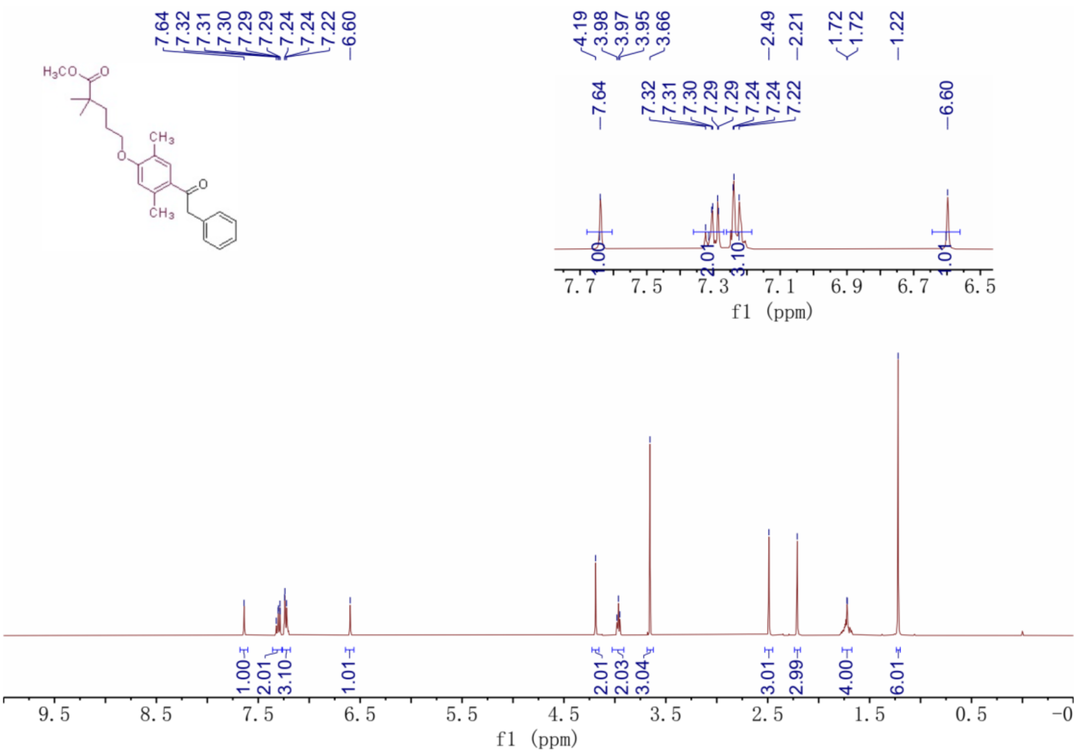

^{13}C NMR (101 MHz, $CDCl_3$) 1-17

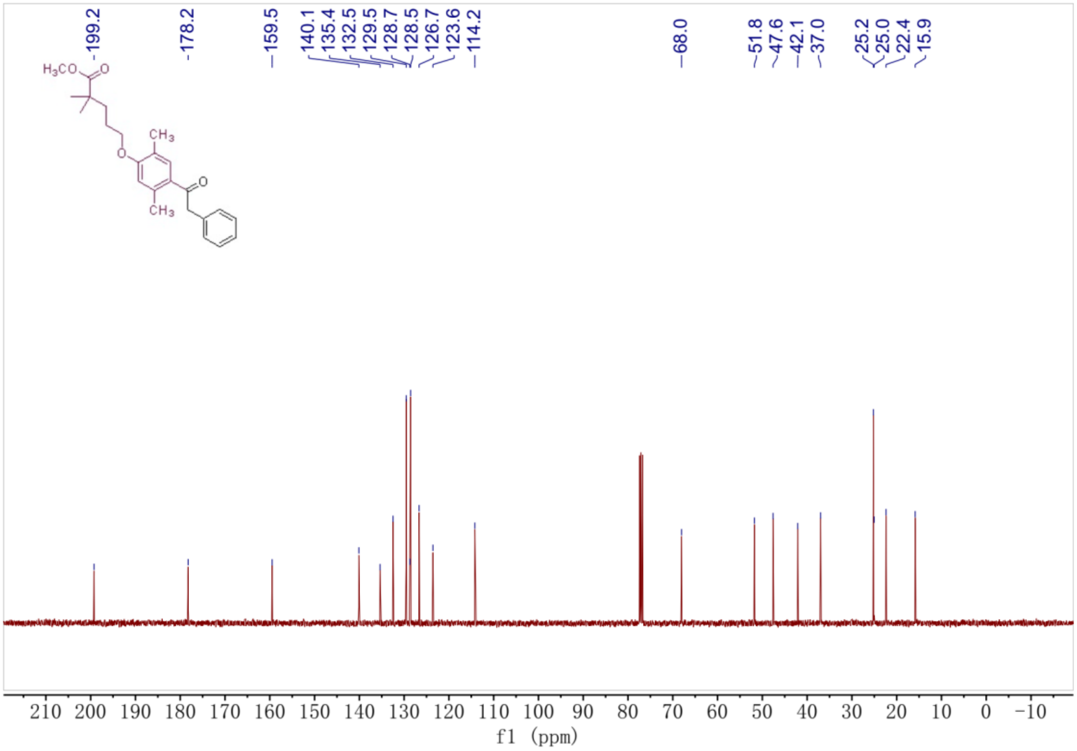

^{1}H NMR (400 MHz, $CDCl_3$) 1-18

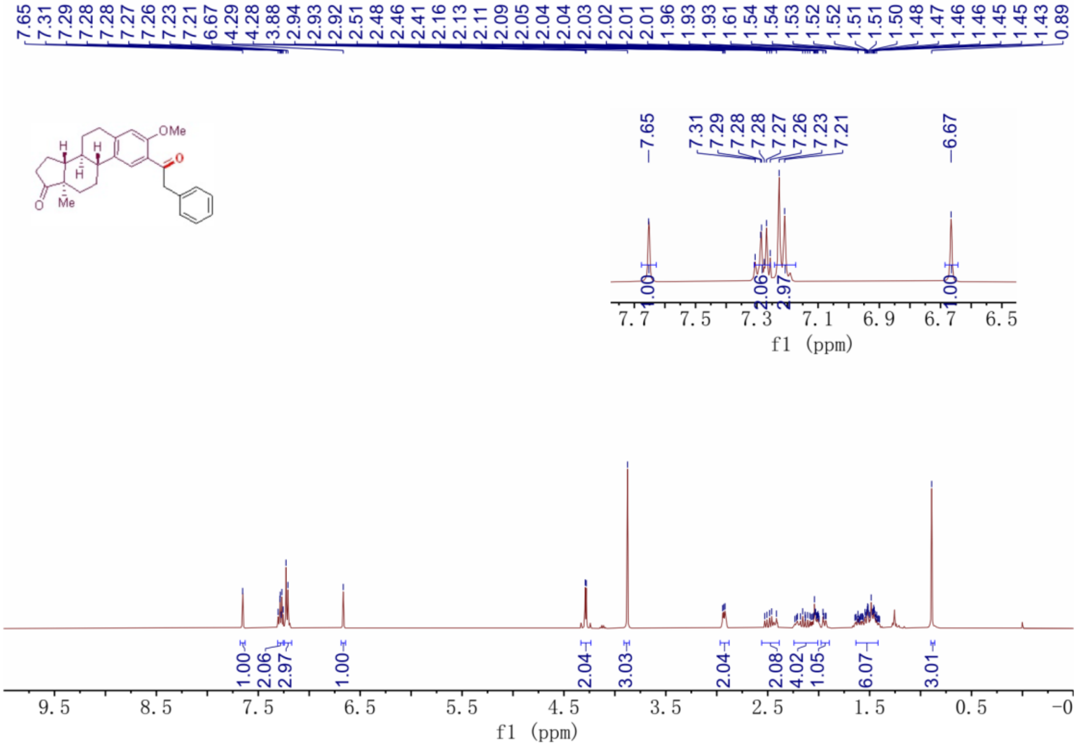

^{13}C NMR (101 MHz, $CDCl_3$) 1-18

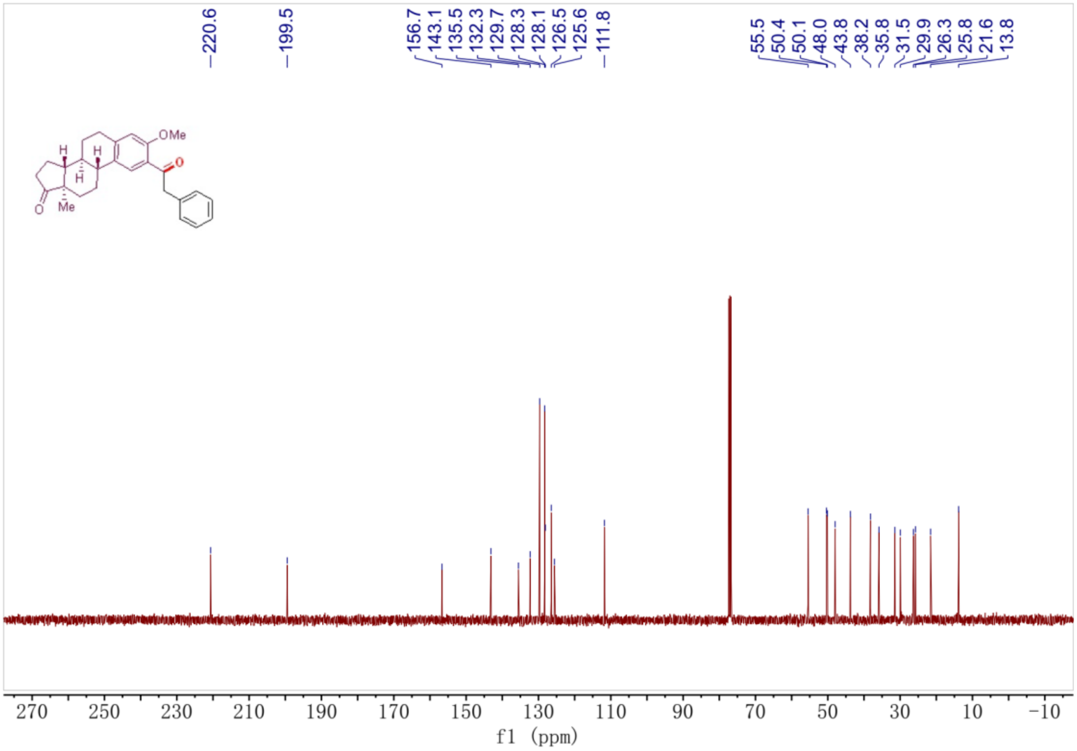

^{1}H NMR (400 MHz, $CDCl_3$) 1-19

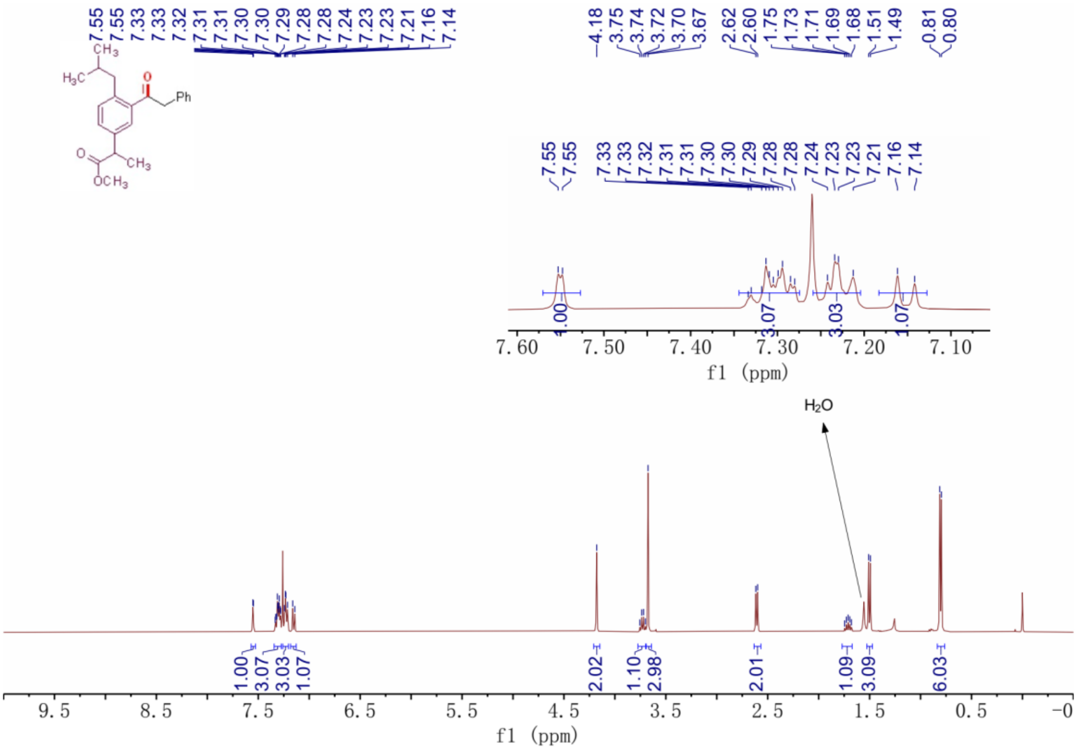

^{13}C NMR (101 MHz, $CDCl_3$) 1-19

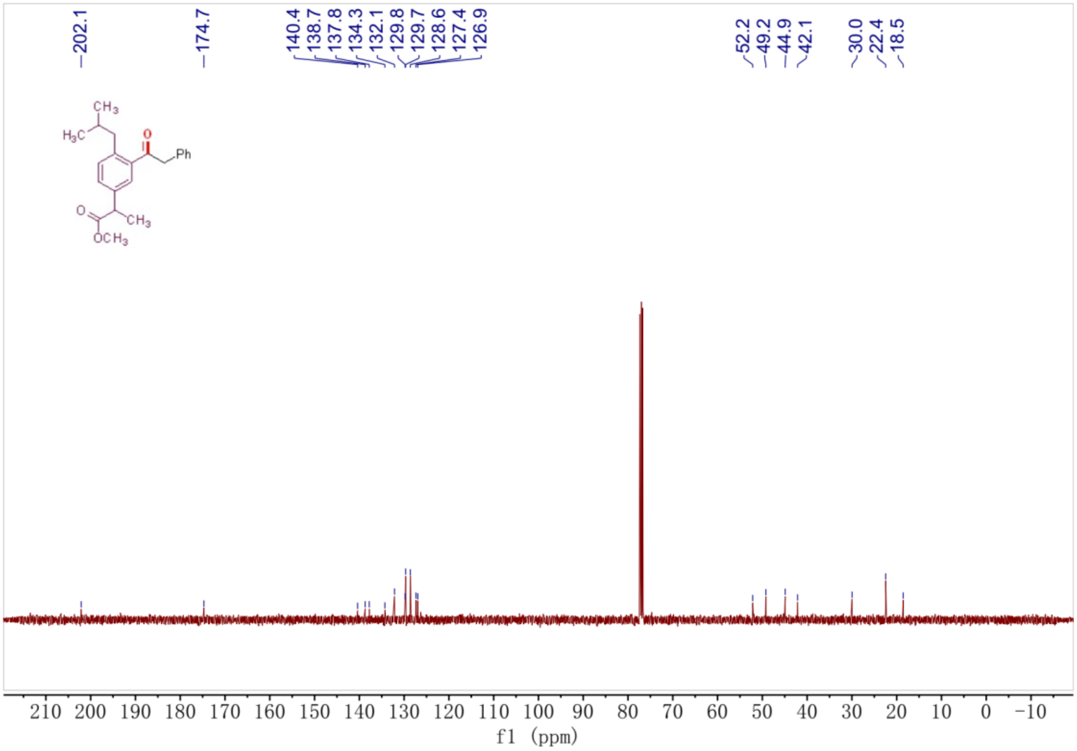

^{1}H NMR (400 MHz, $CDCl_3$) 1-20

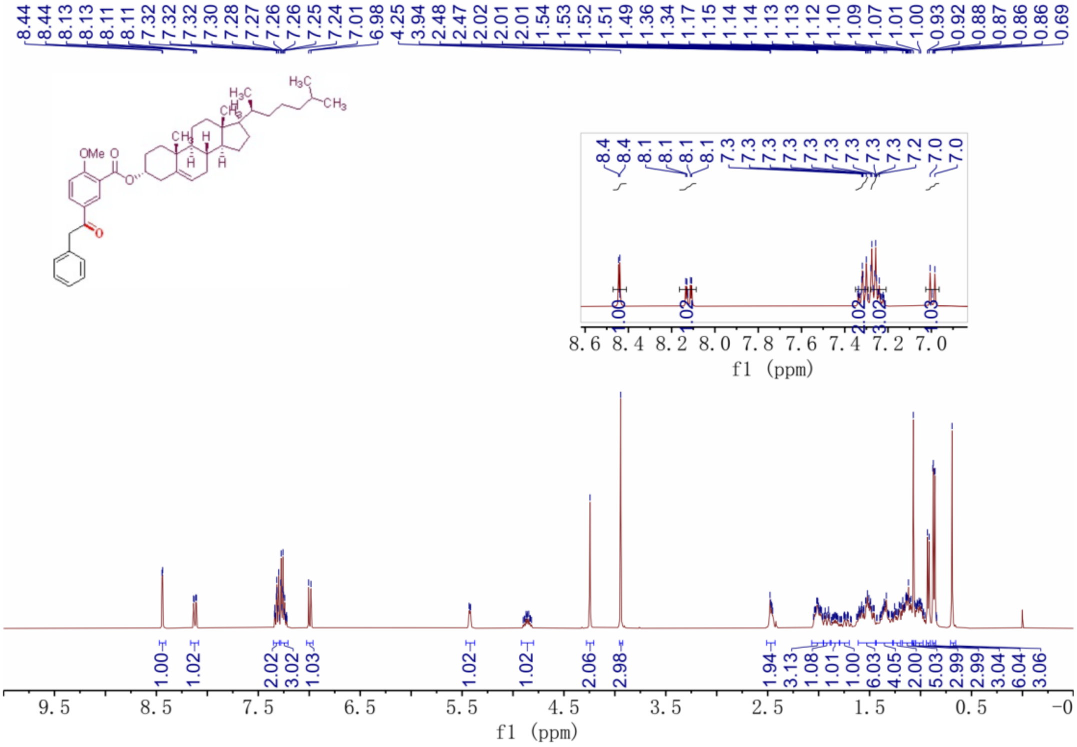

^{13}C NMR (101 MHz, $CDCl_3$) 1-20

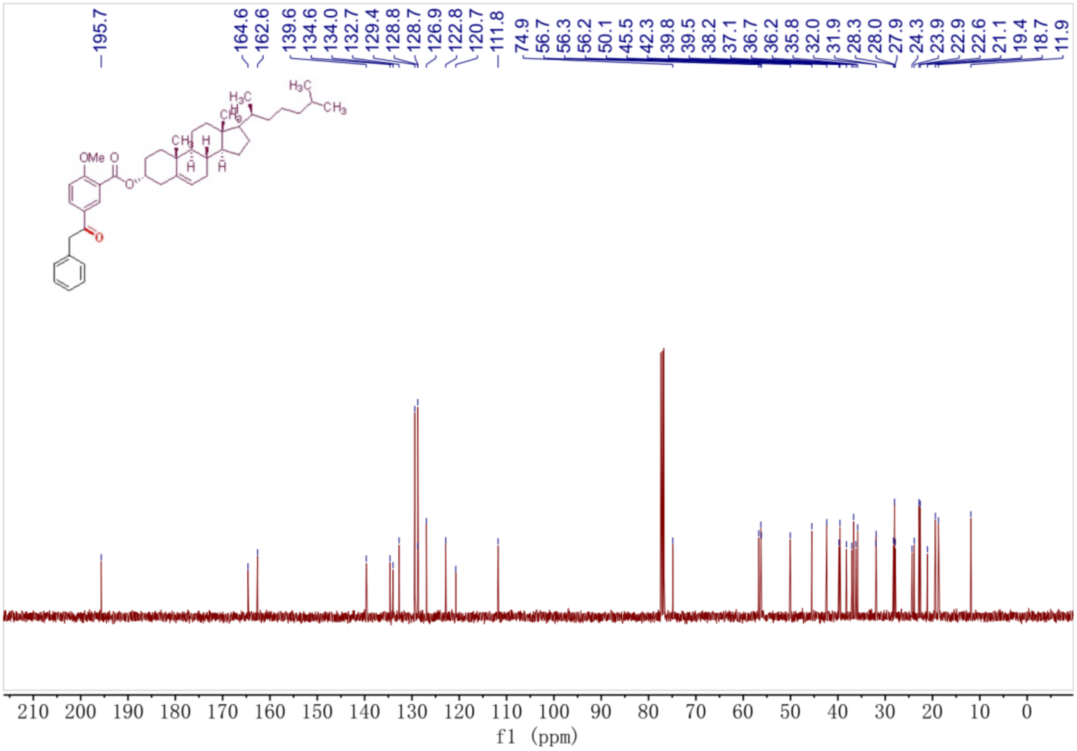

^{1}H NMR (400 MHz, $CDCl_3$) 1-21

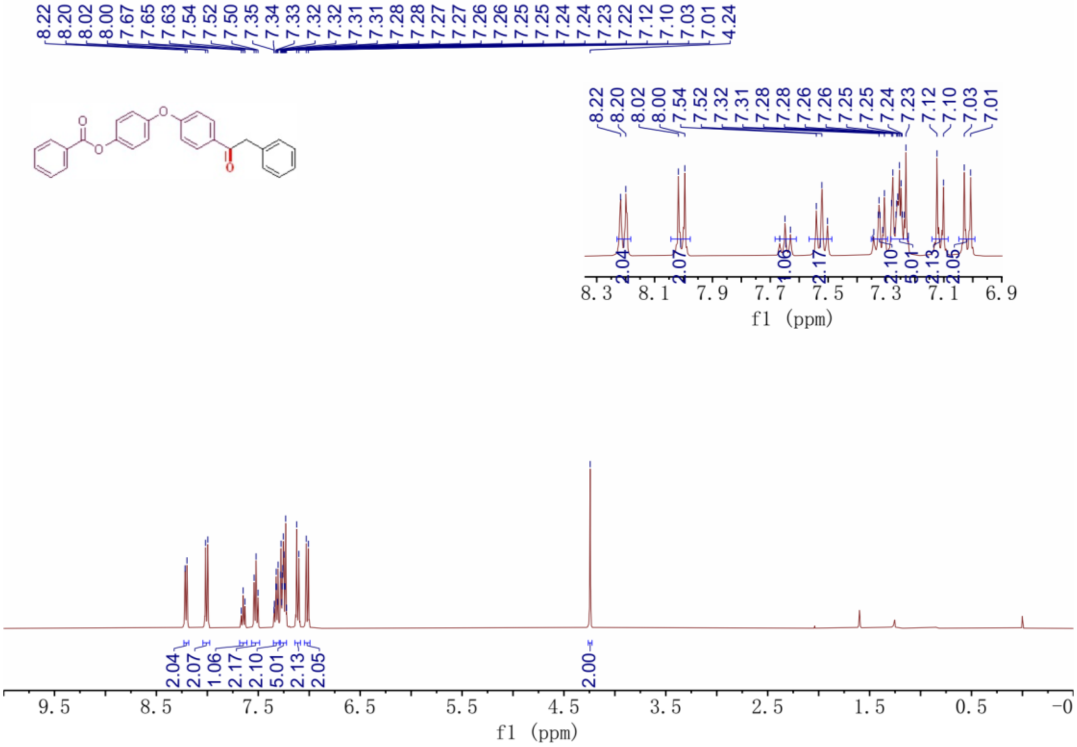

^{13}C NMR (101 MHz, $CDCl_3$) 1-21

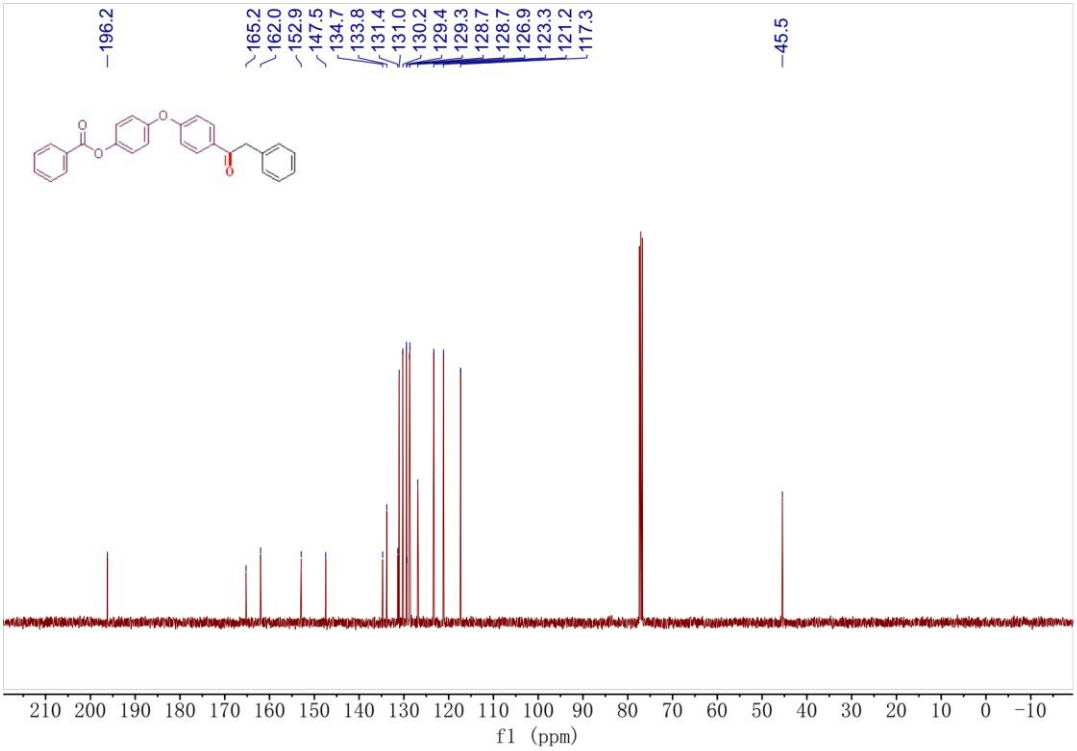

^{1}H NMR (400 MHz, $CDCl_3$) 1-22

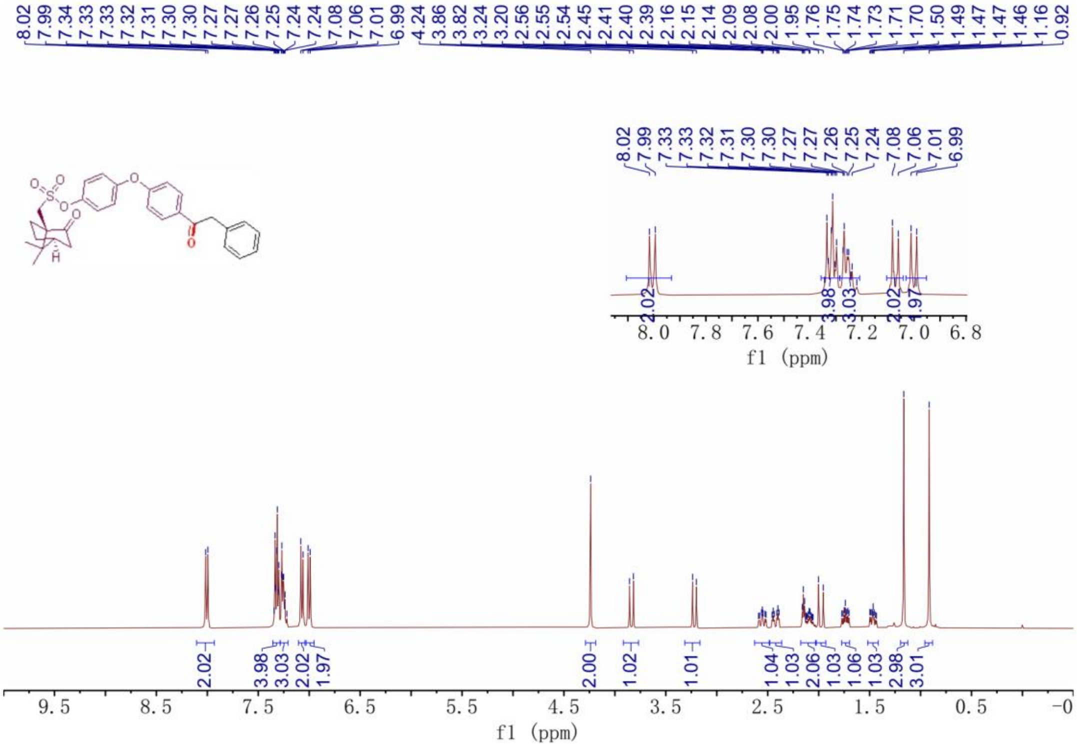

^{13}C NMR (101 MHz, $CDCl_3$) 1-22

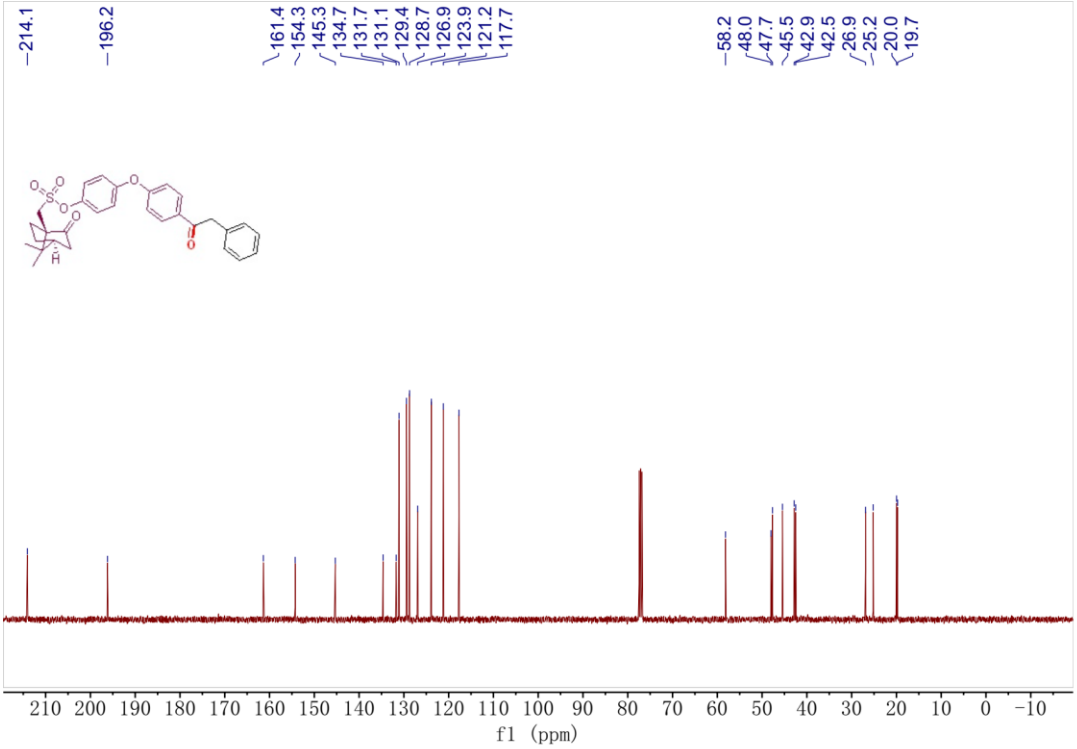

^{1}H NMR (400 MHz, $CDCl_3$) 1-23

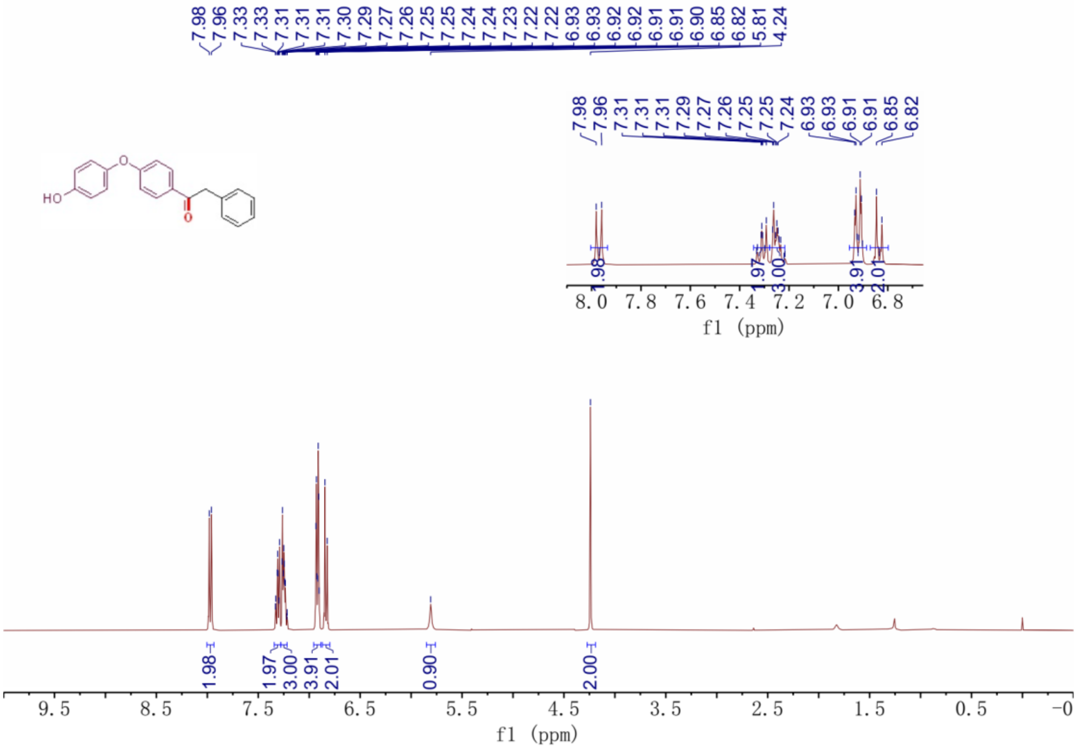

^{13}C NMR (101 MHz, $CDCl_3$) 1-23

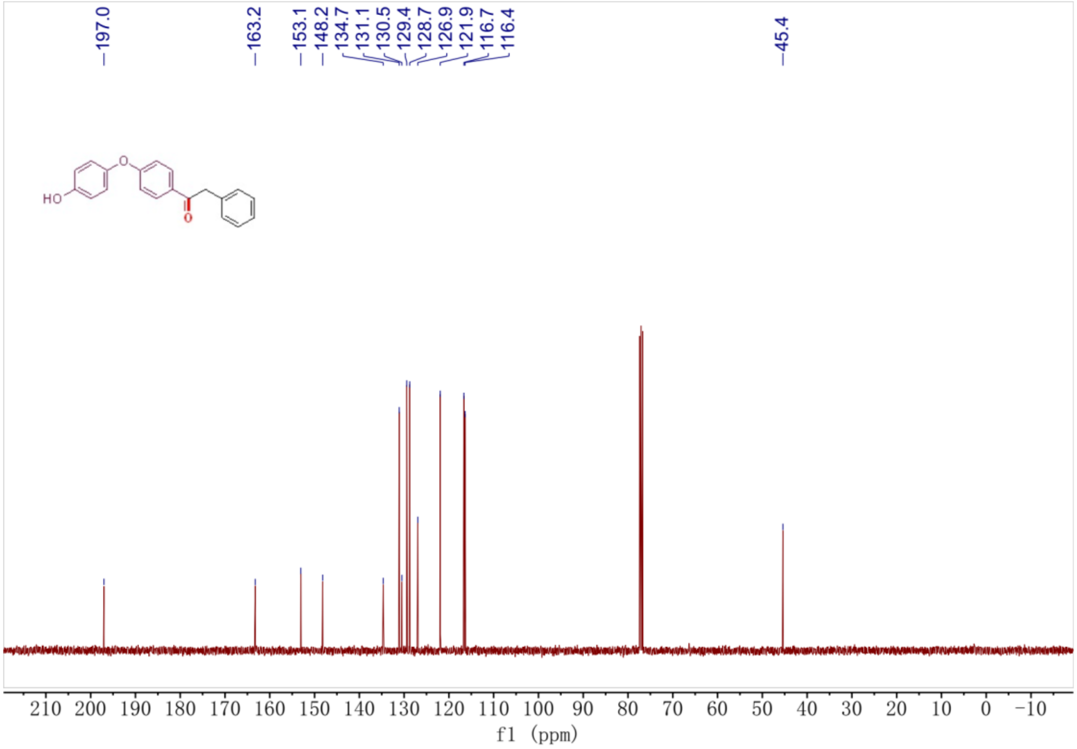

^{1}H NMR (400 MHz, $CDCl_3$) 2-1

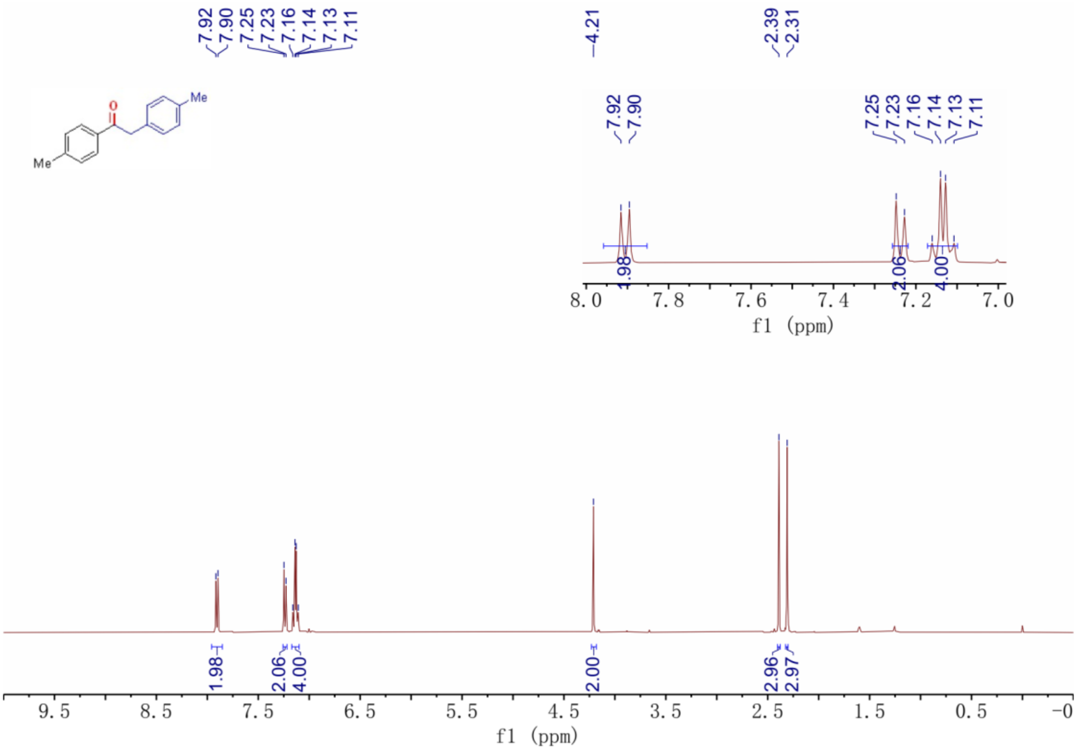

^{13}C NMR (101 MHz, $CDCl_3$) 2-1

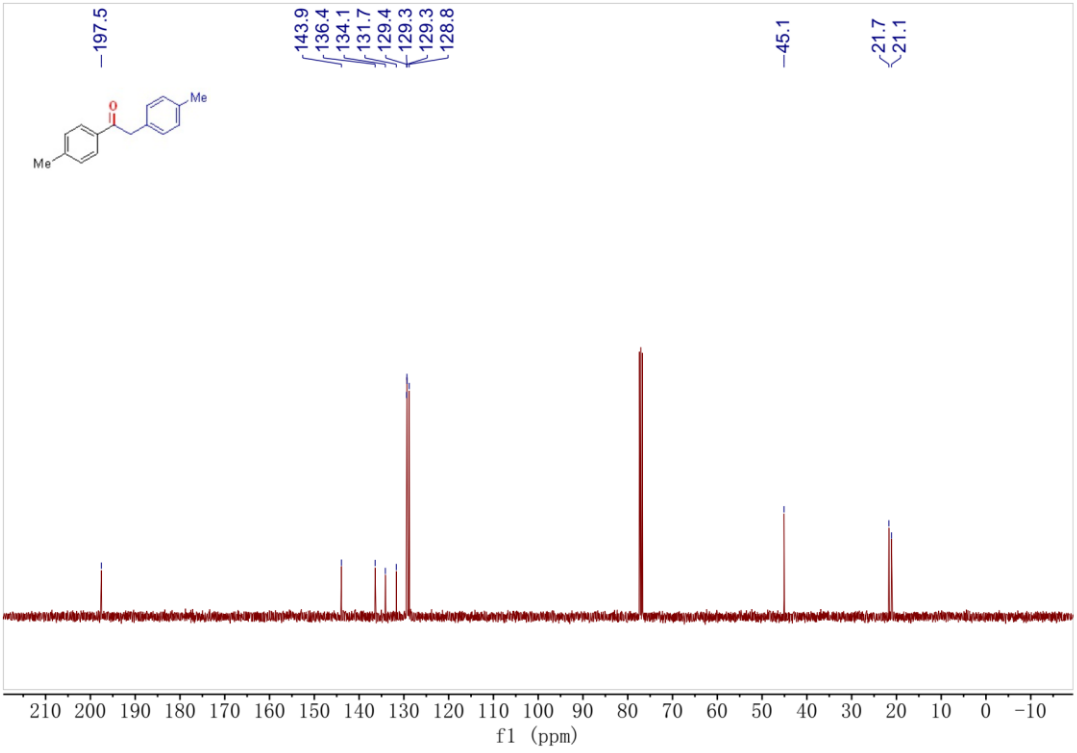

^{1}H NMR (400 MHz, $CDCl_3$) 2-2

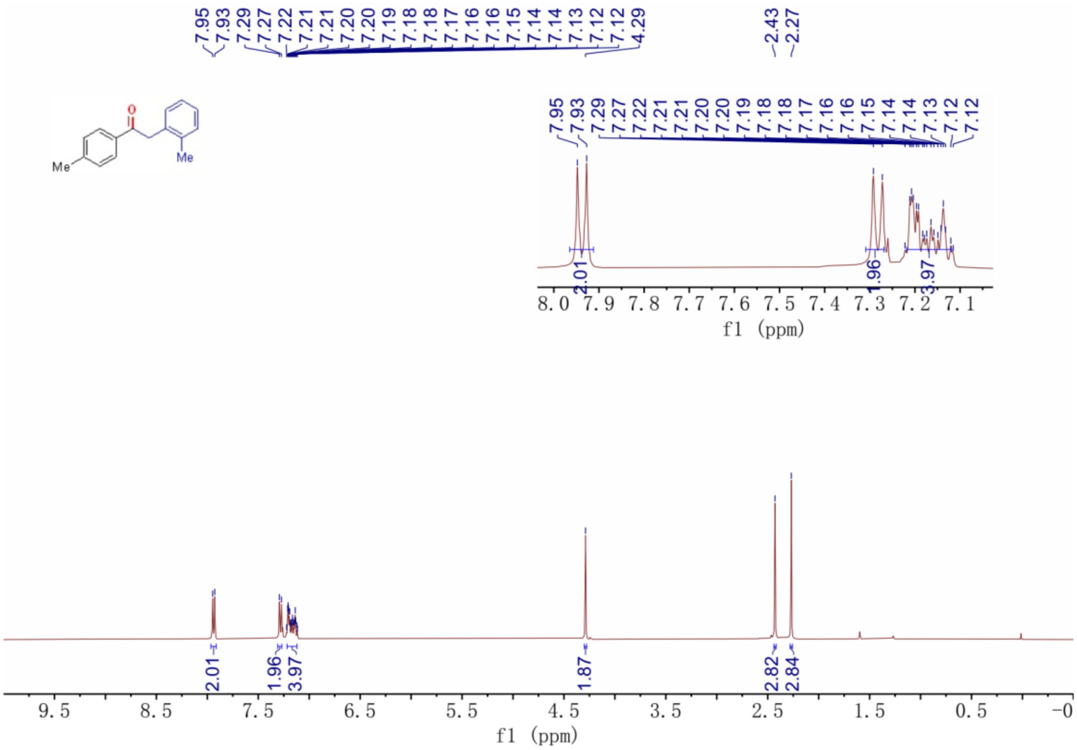

^{13}C NMR (101 MHz, $CDCl_3$) 2-2

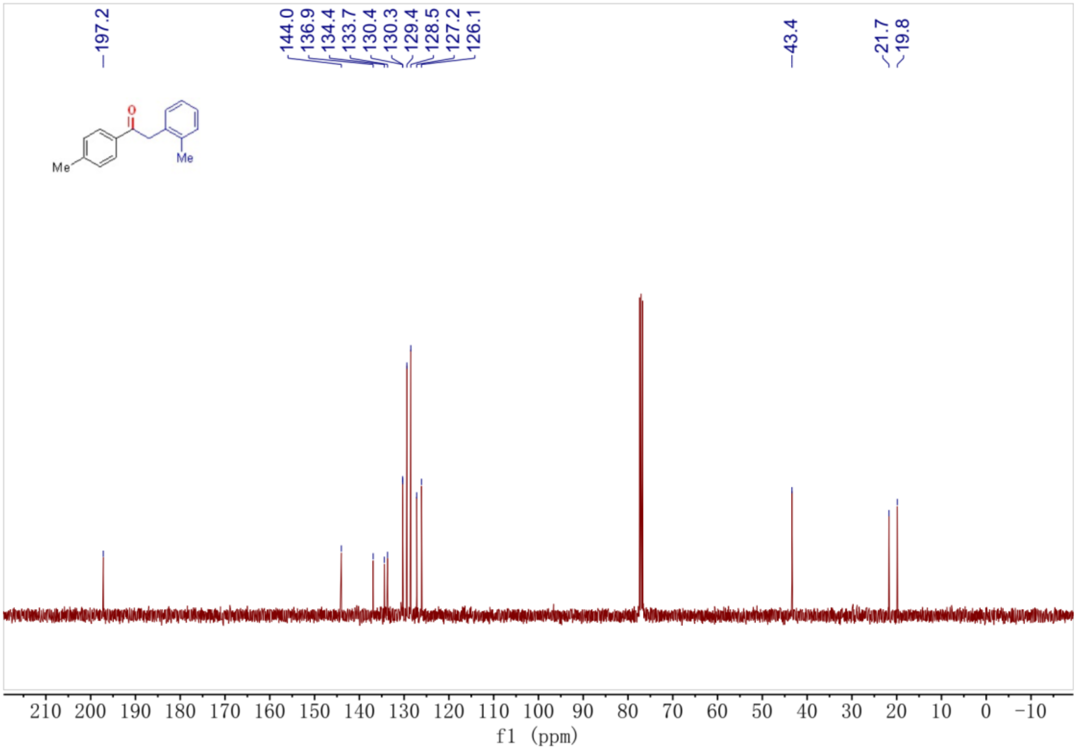

^{1}H NMR (400 MHz, $CDCl_3$) 2-3

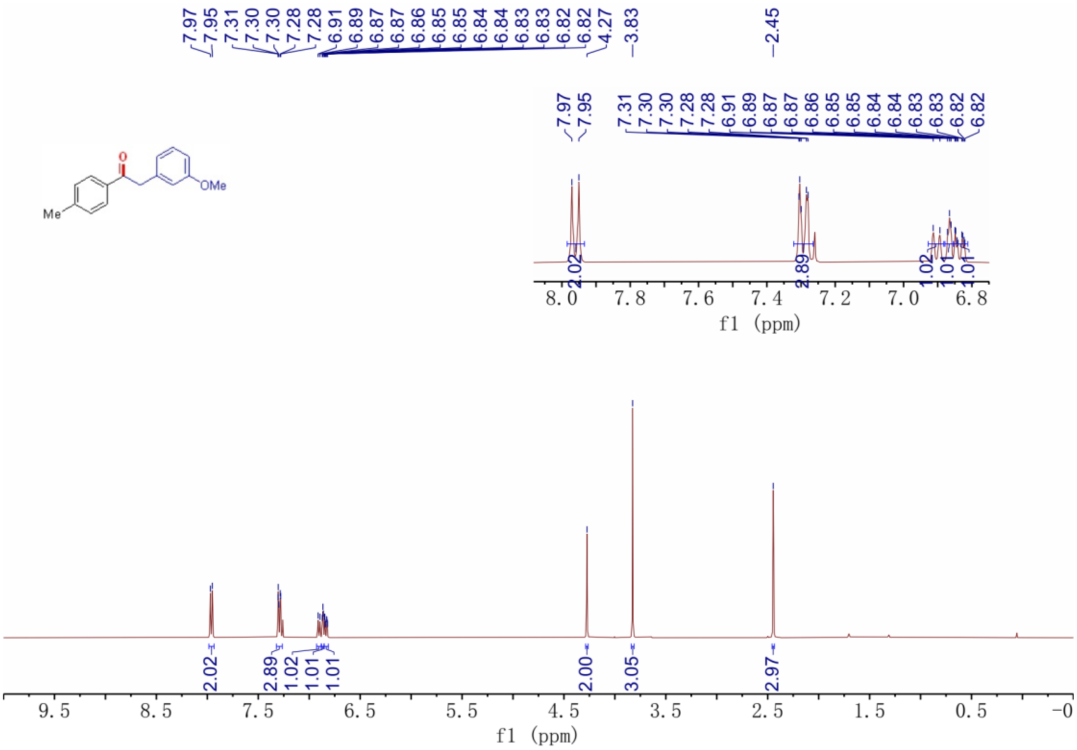

^{13}C NMR (101 MHz, $CDCl_3$) 2-3

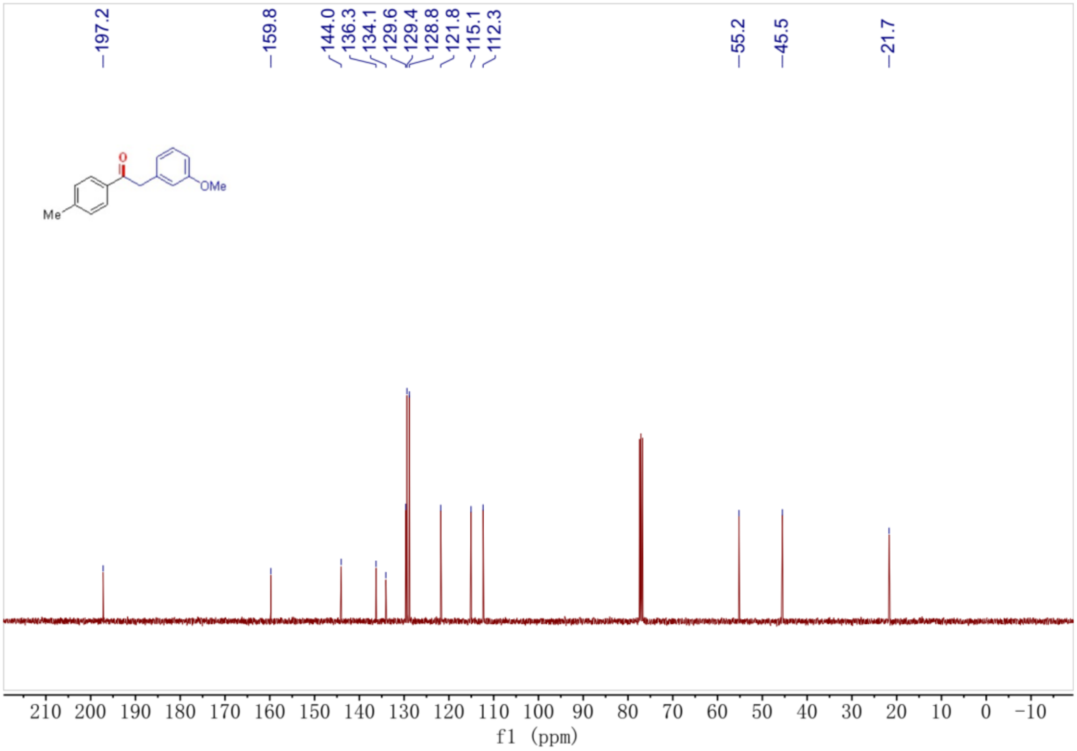

^{1}H NMR (400 MHz, CDCl$_3$) 2-4

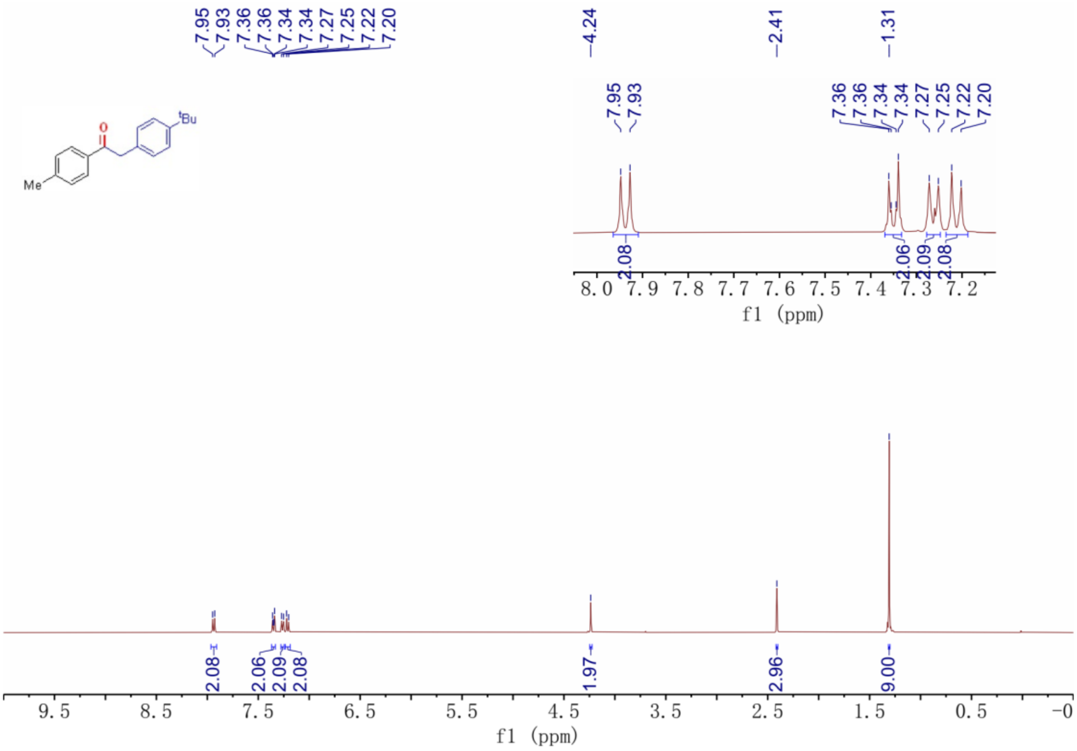

^{13}C NMR (101 MHz, CDCl$_3$) 2-4

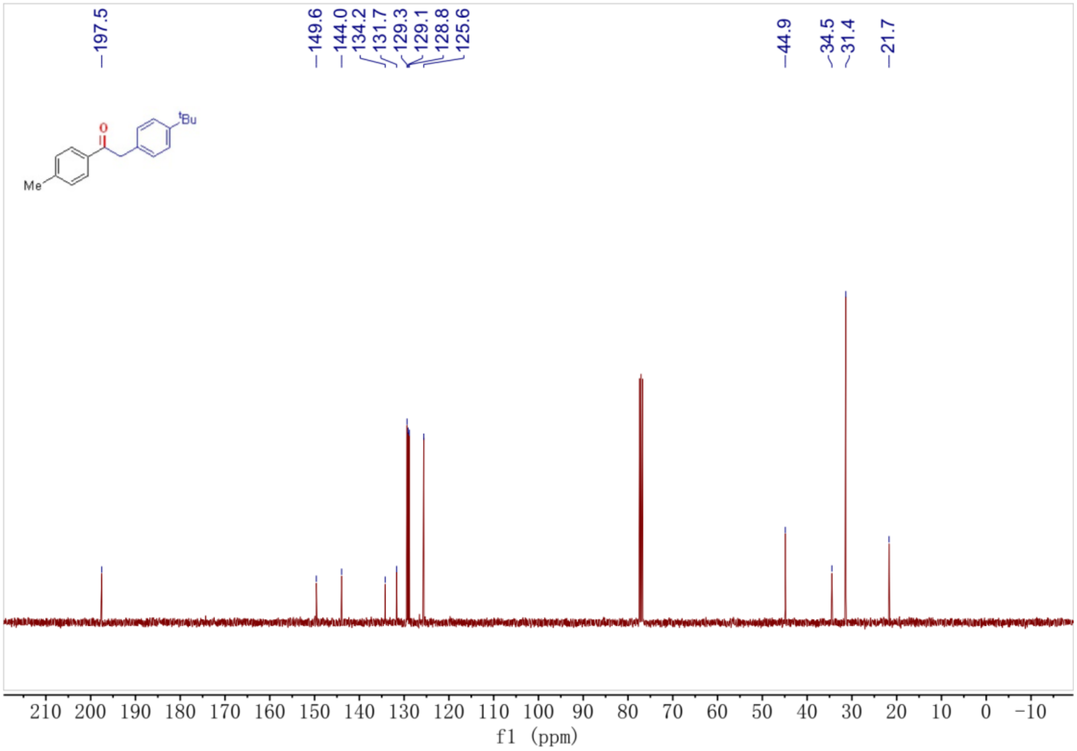

^{1}H NMR (400 MHz, $CDCl_3$) 2-5

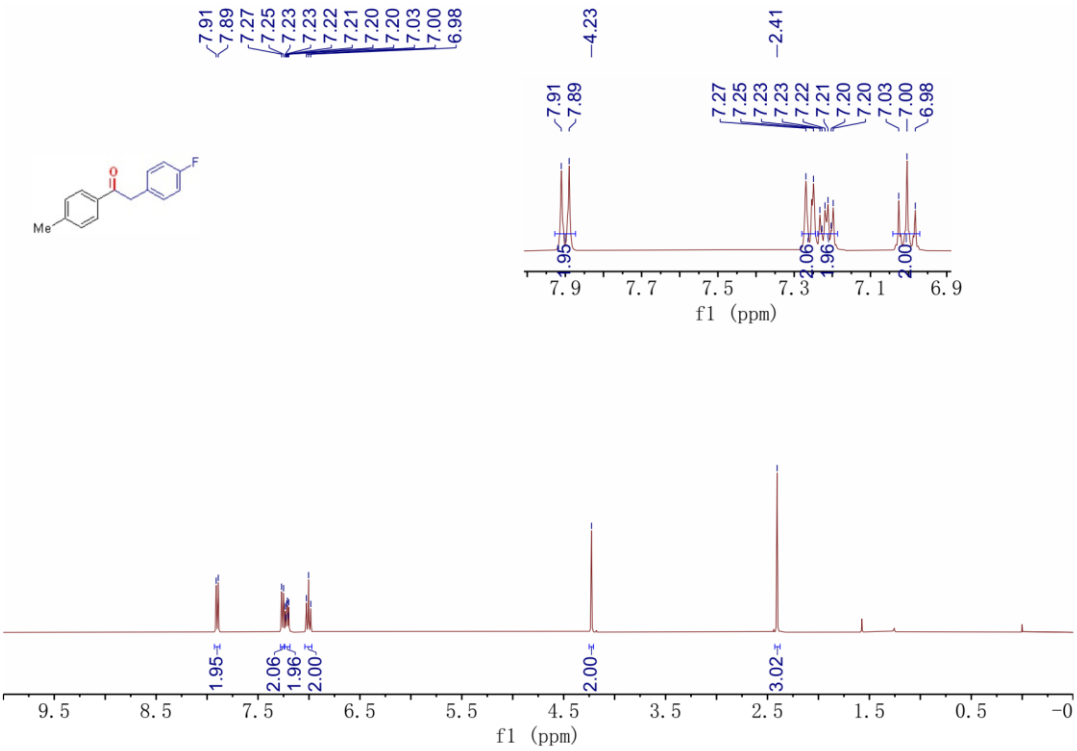

^{13}C NMR (101 MHz, $CDCl_3$) 2-5

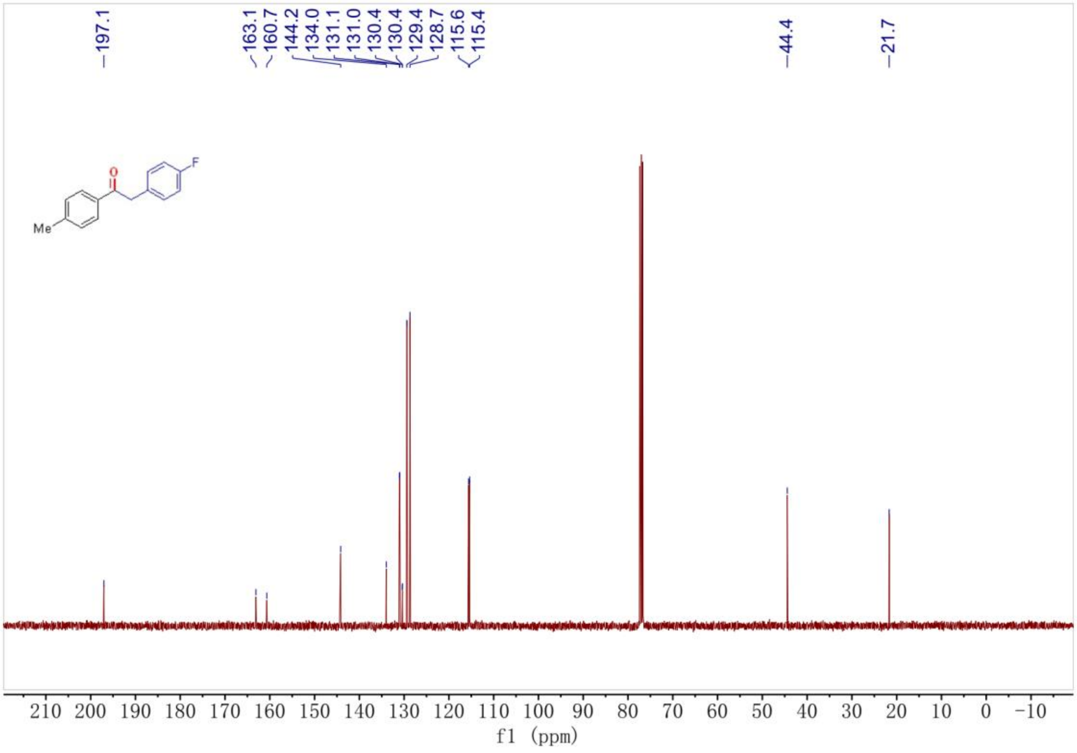

^{19}F NMR (376 MHz, CDCl$_3$) 2-5

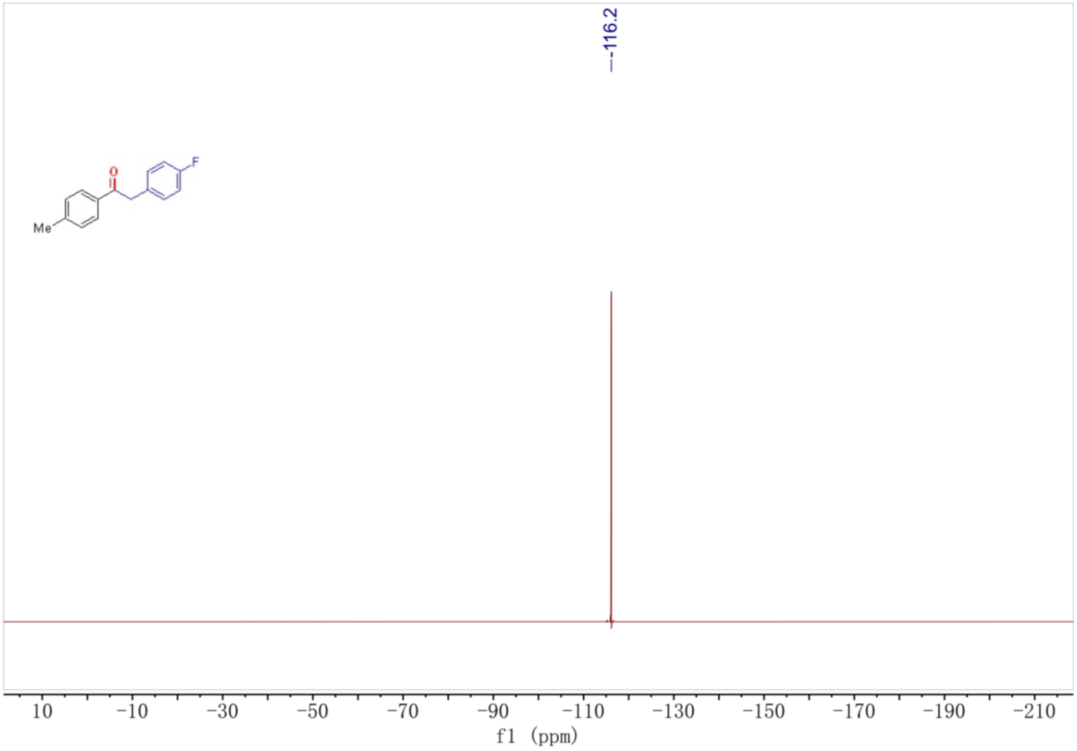

^{1}H NMR (400 MHz, $CDCl_3$) 2-6

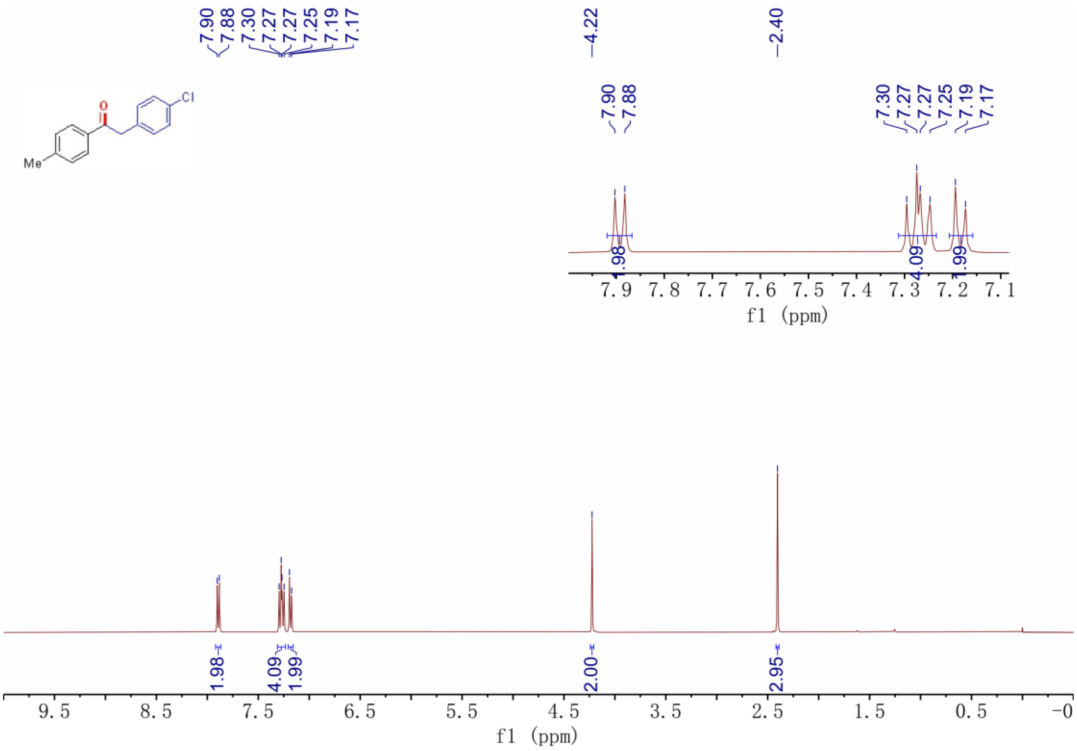

^{13}C NMR (101 MHz, $CDCl_3$) 2-6

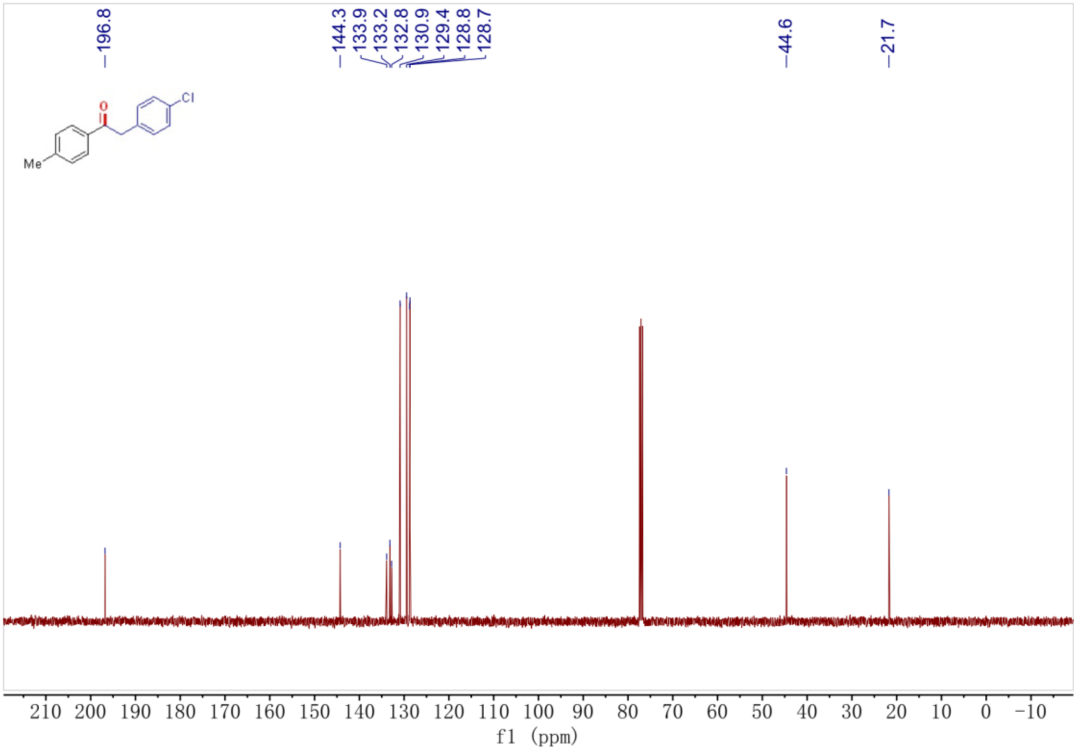

^{1}H NMR (400 MHz, $CDCl_3$) 2-7

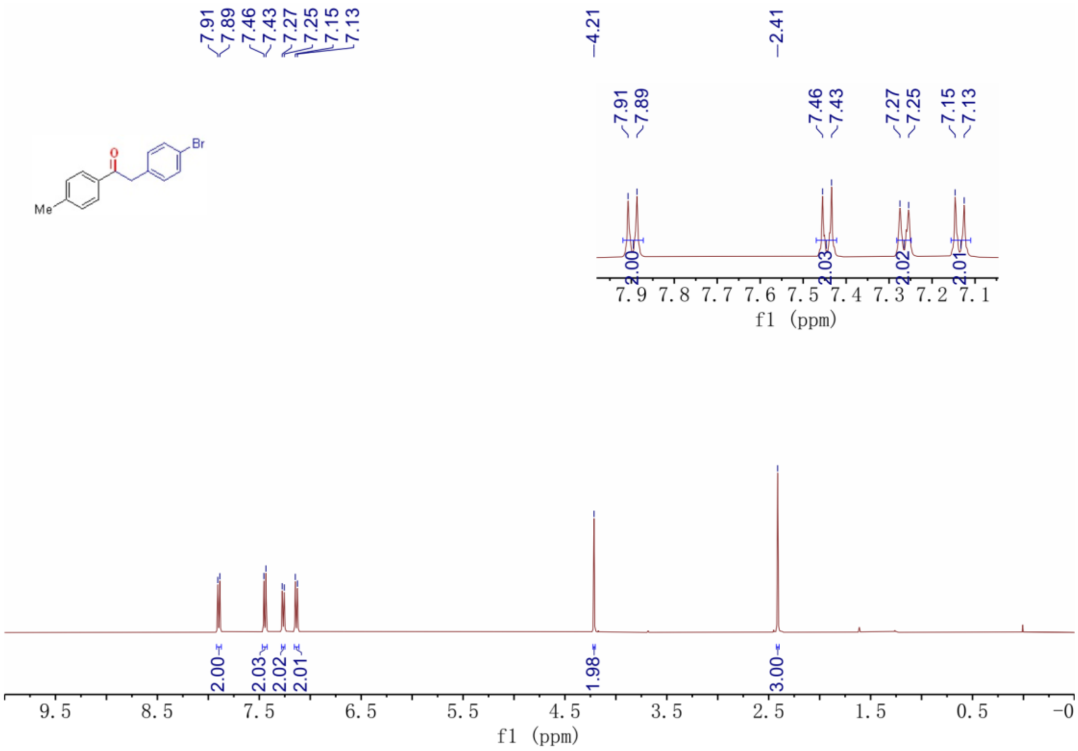

^{13}C NMR (101 MHz, $CDCl_3$) 2-7

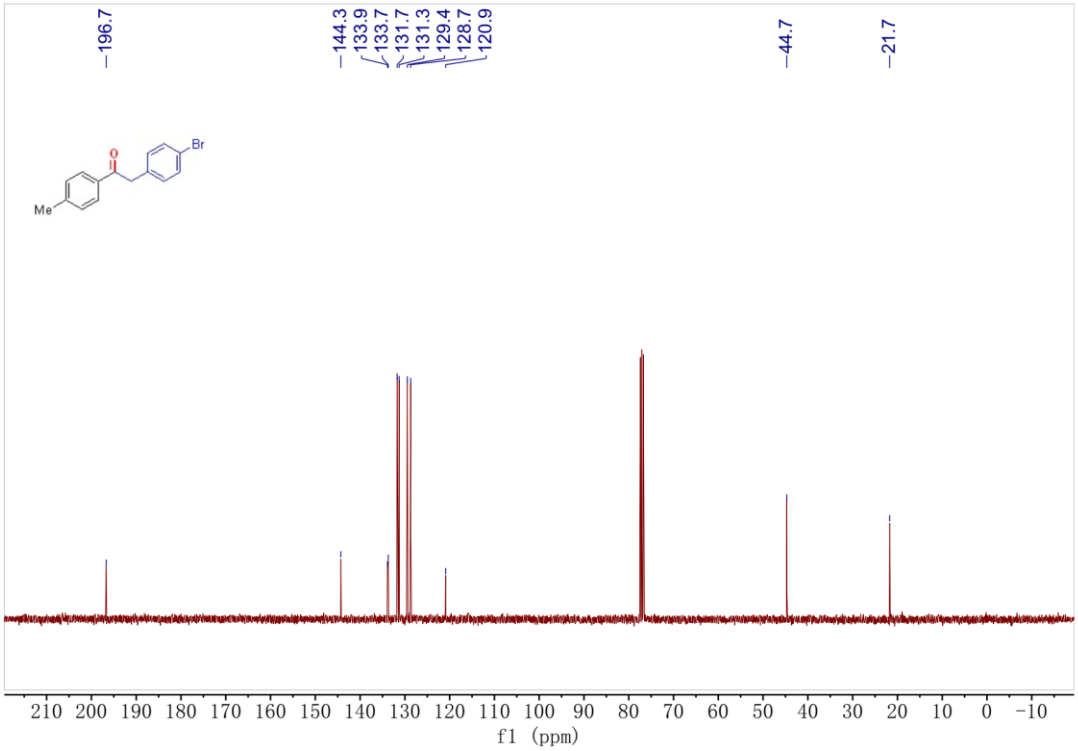

^{1}H NMR (400 MHz, $CDCl_3$) 2-8

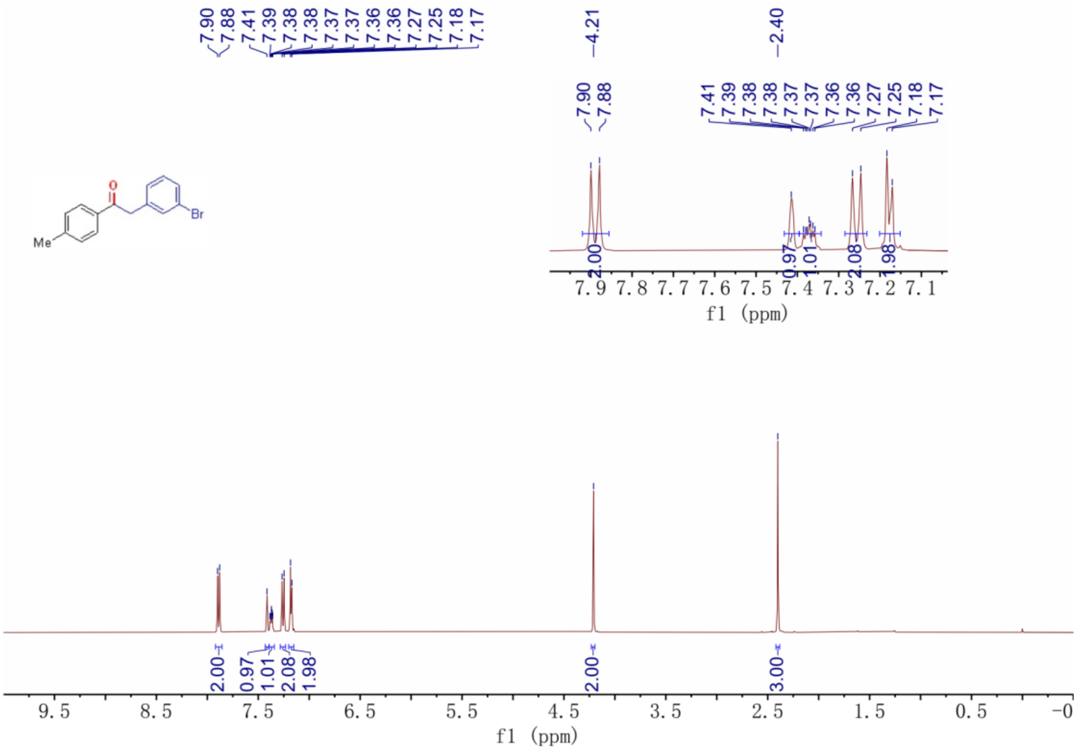

^{13}C NMR (101 MHz, $CDCl_3$) 2-8

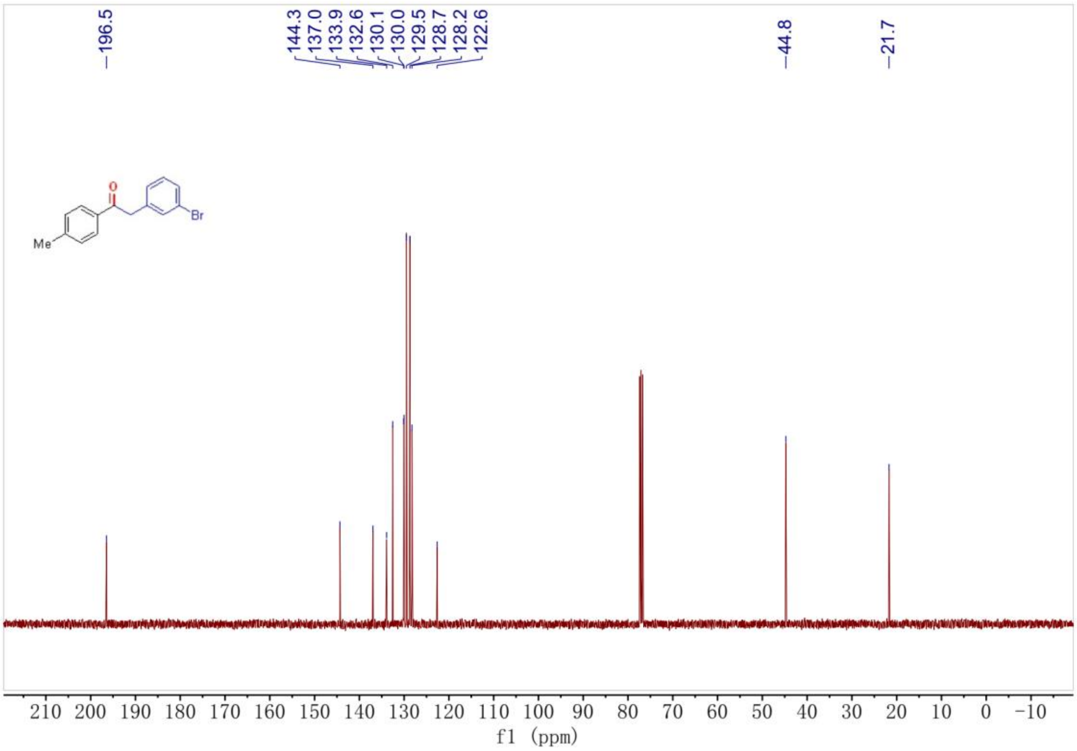

^{1}H NMR (400 MHz, $CDCl_3$) 2-9

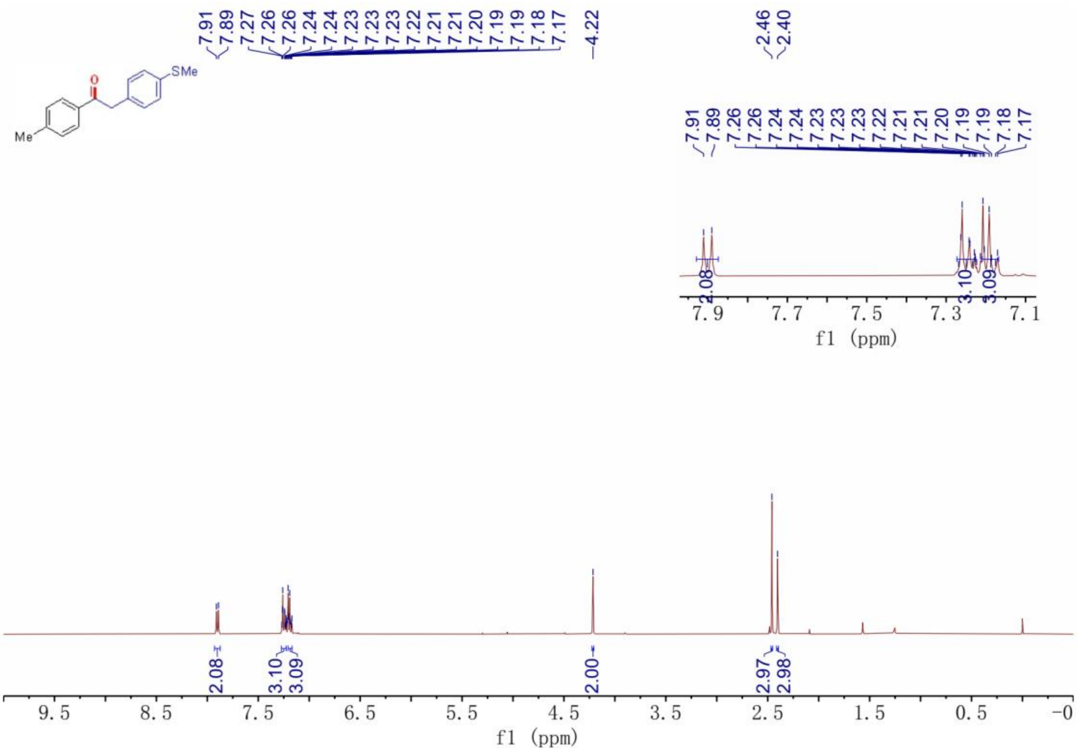

^{13}C NMR (101 MHz, $CDCl_3$) 2-9

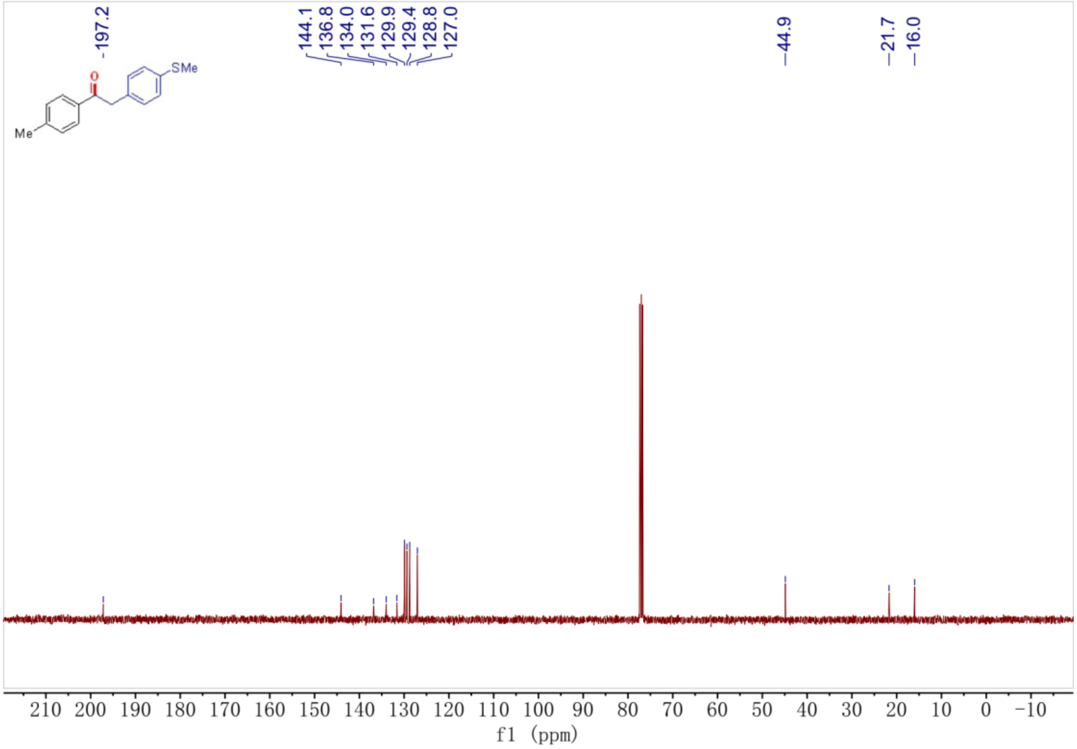

^{1}H NMR (400 MHz, $CDCl_3$) 2-10

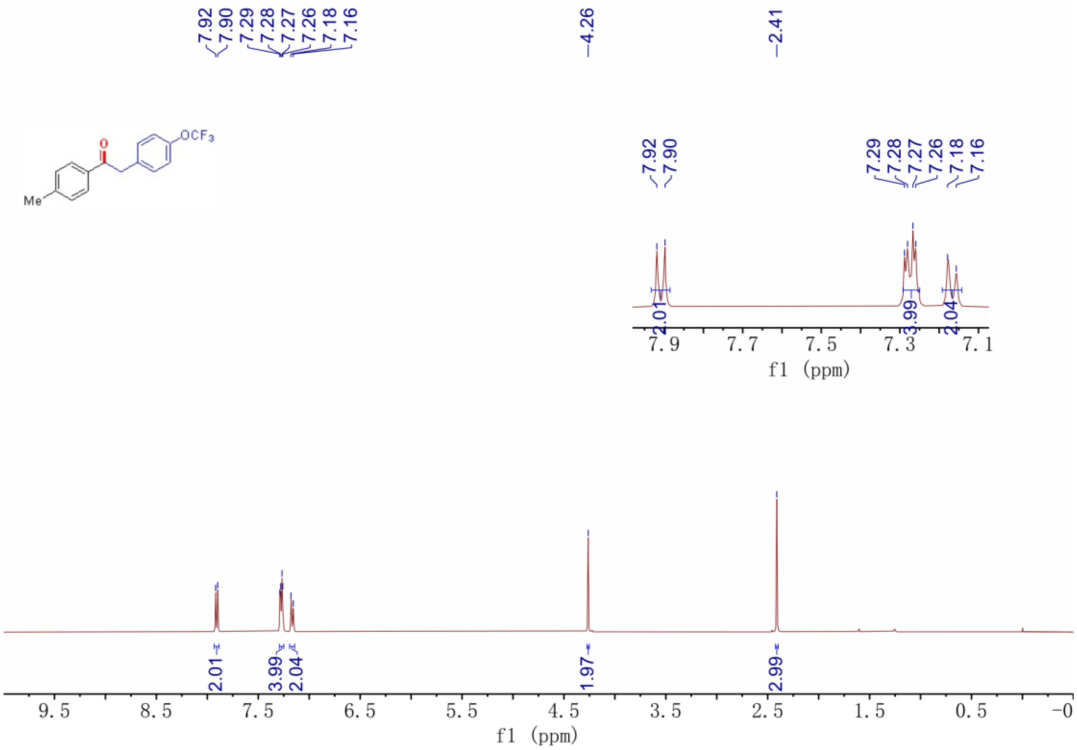

^{13}C NMR (101 MHz, $CDCl_3$) 2-10

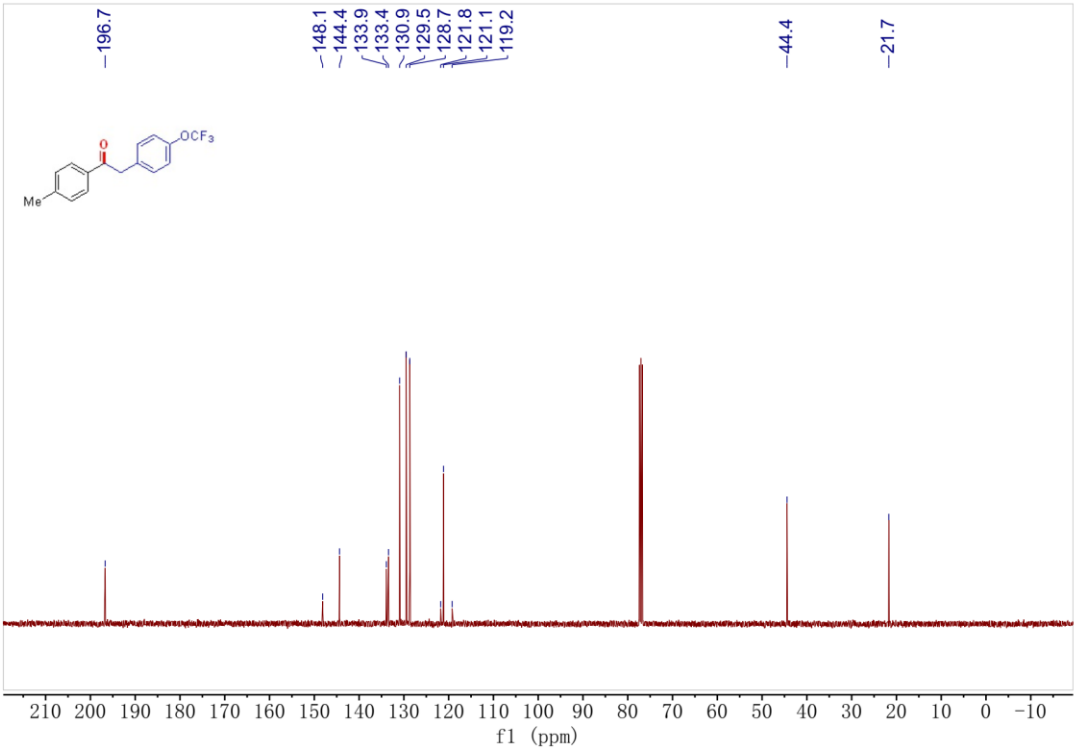

^{19}F NMR (376 MHz, $CDCl_3$) 2-10

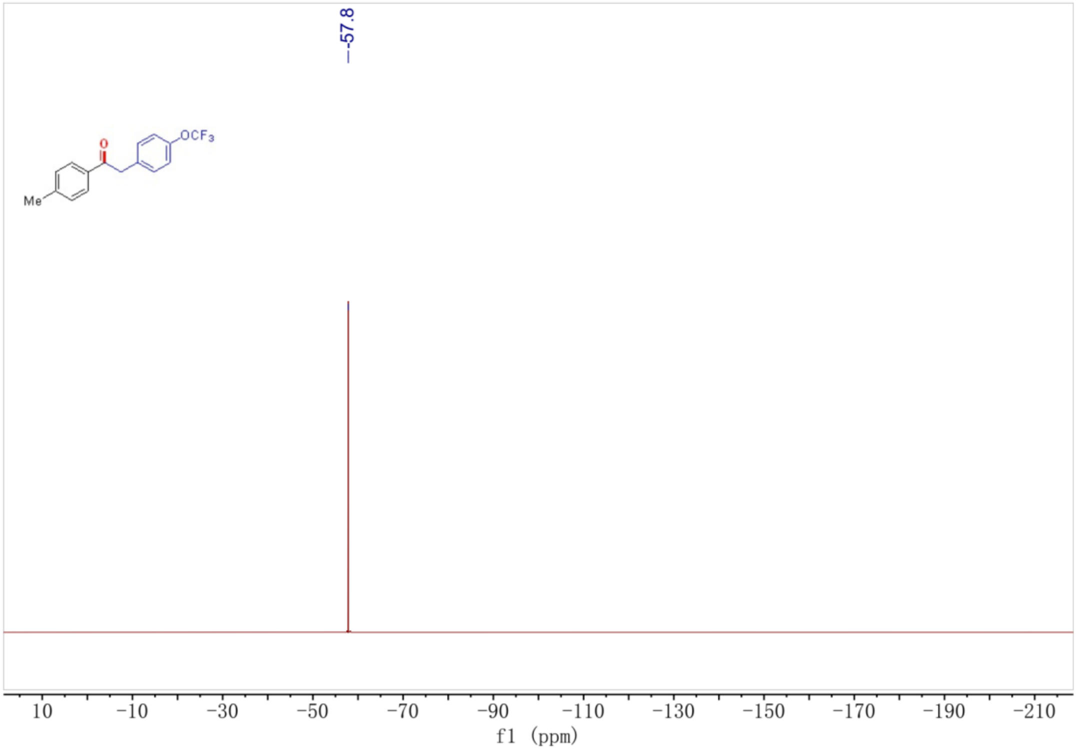

^{1}H NMR (400 MHz, $CDCl_3$) 2-11

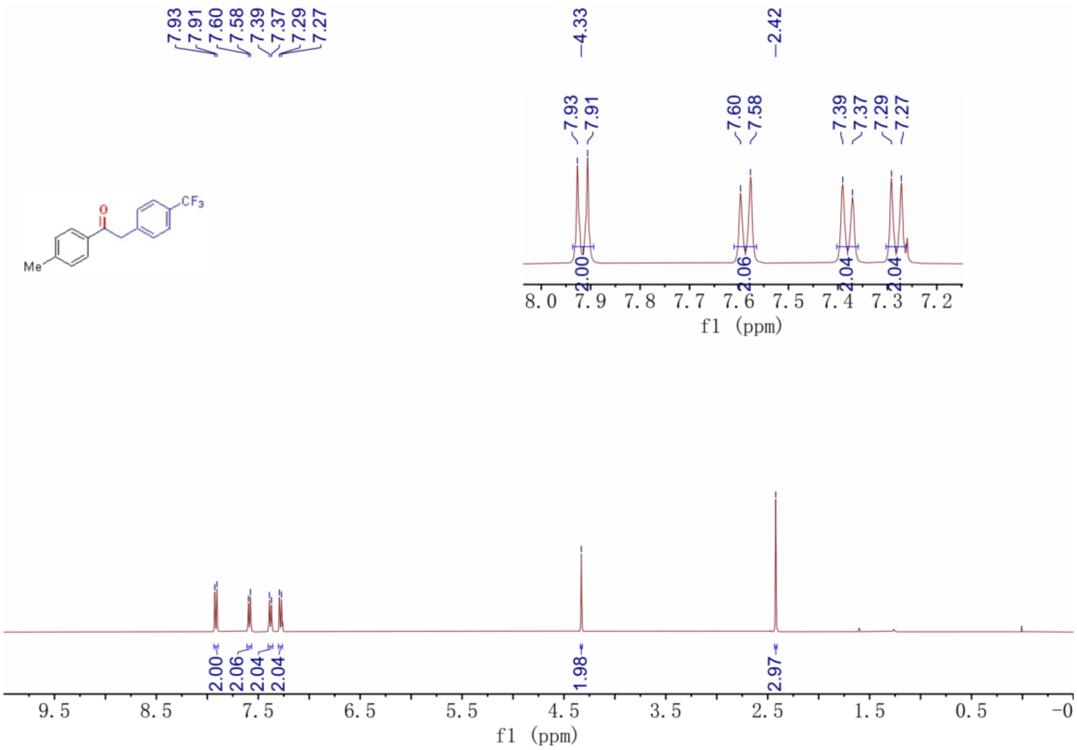

^{13}C NMR (101 MHz, $CDCl_3$) 2-11

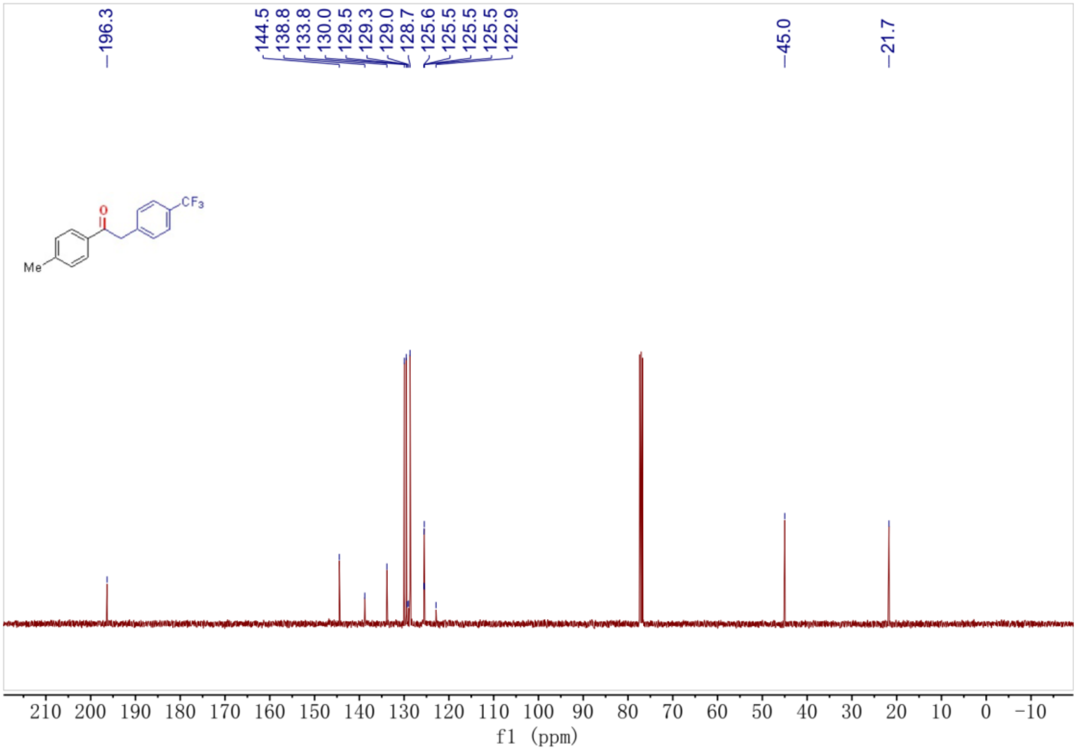

^{19}F NMR (376 MHz, $CDCl_3$) 2-11

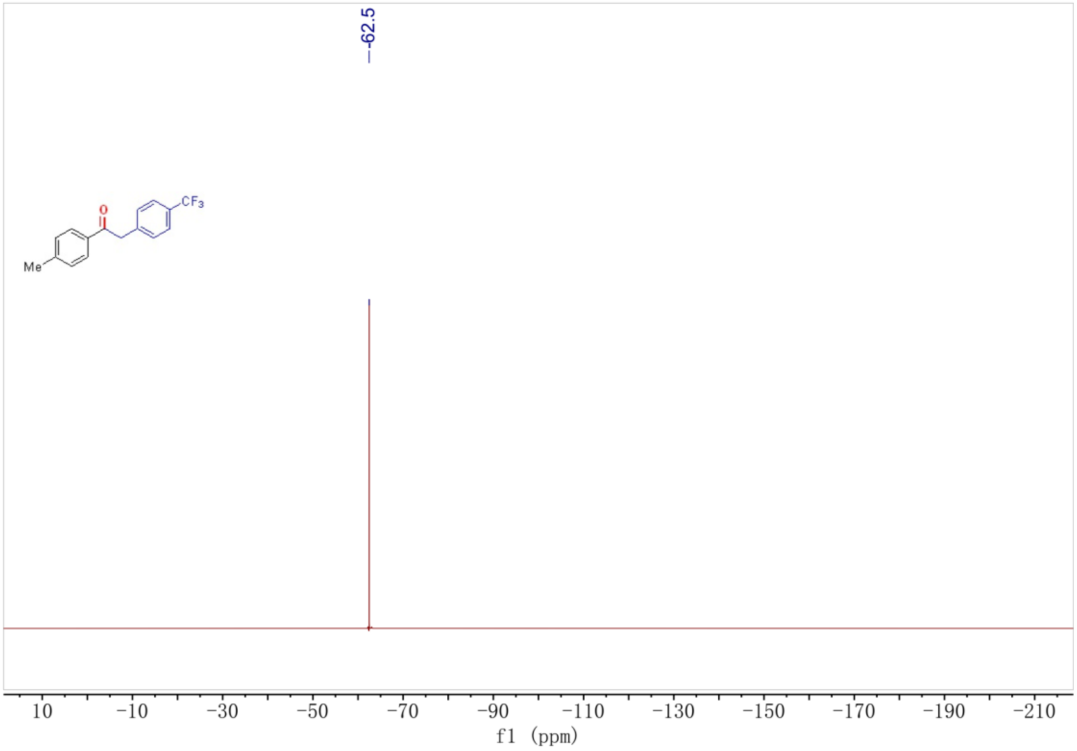

^{1}H NMR (400 MHz, $CDCl_3$) 2-12

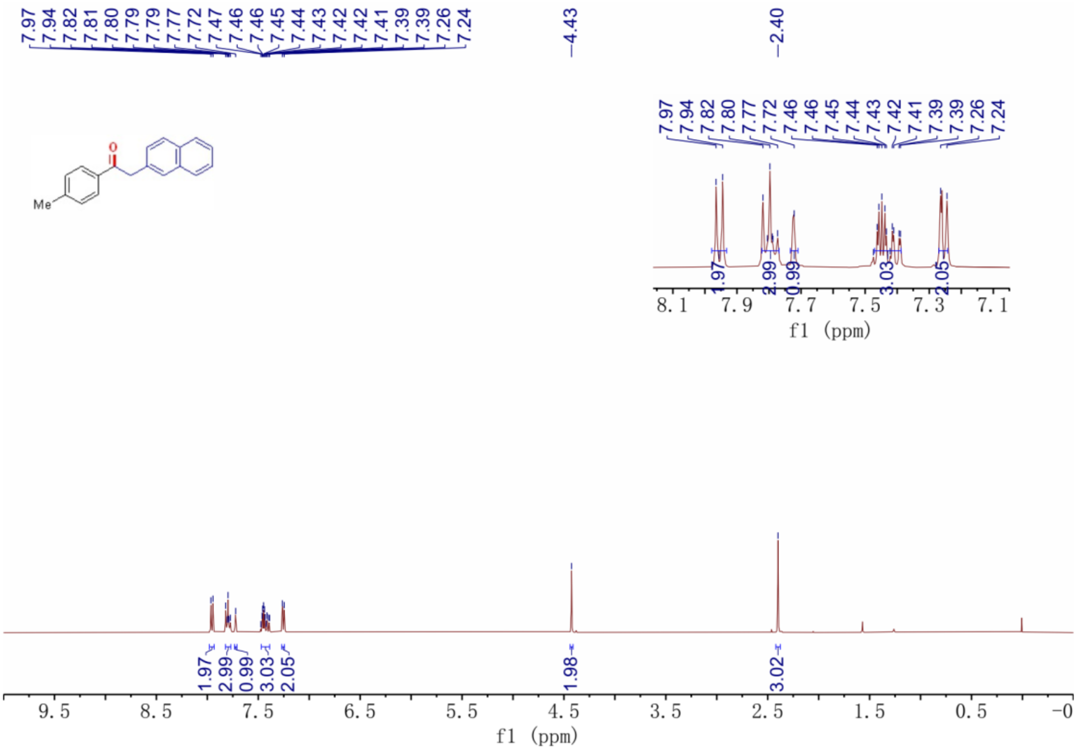

^{13}C NMR (101 MHz, $CDCl_3$) 2-12

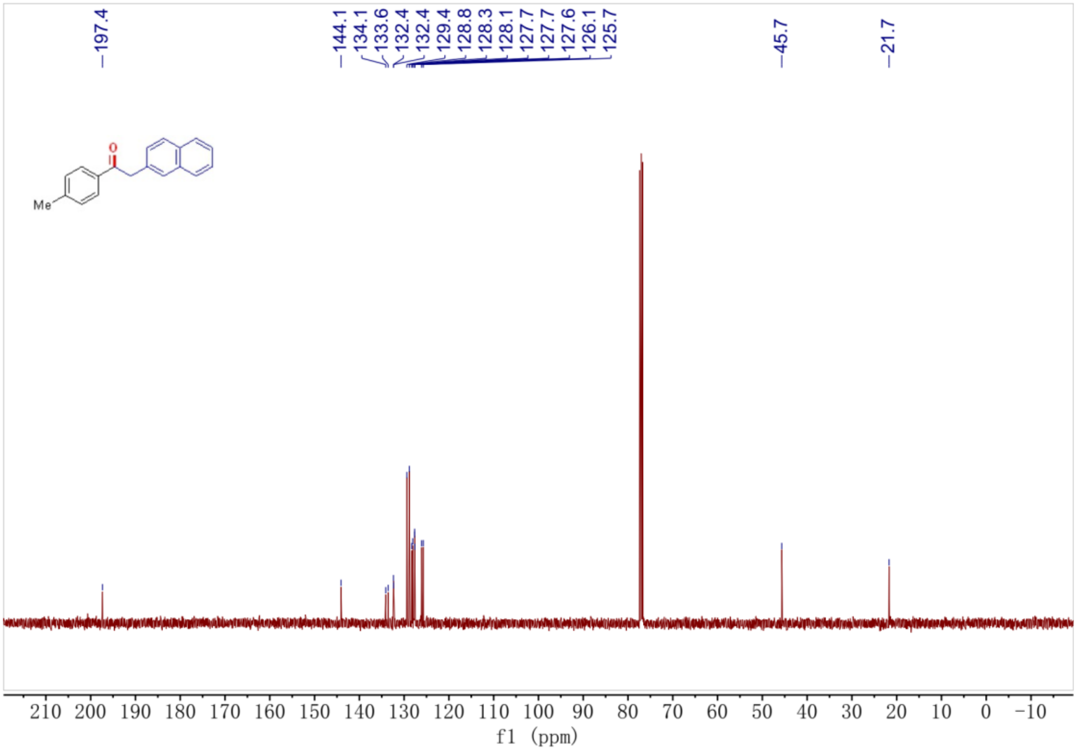

^{1}H NMR (400 MHz, $CDCl_3$) 2-13

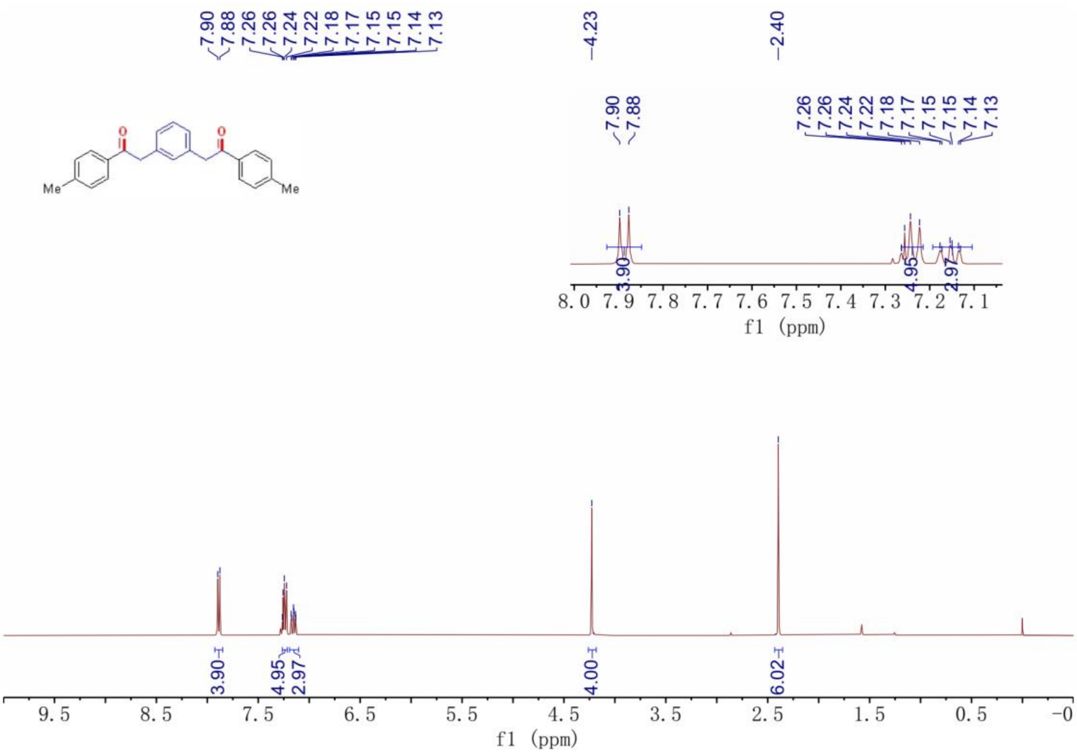

^{13}C NMR (101 MHz, $CDCl_3$) 2-13

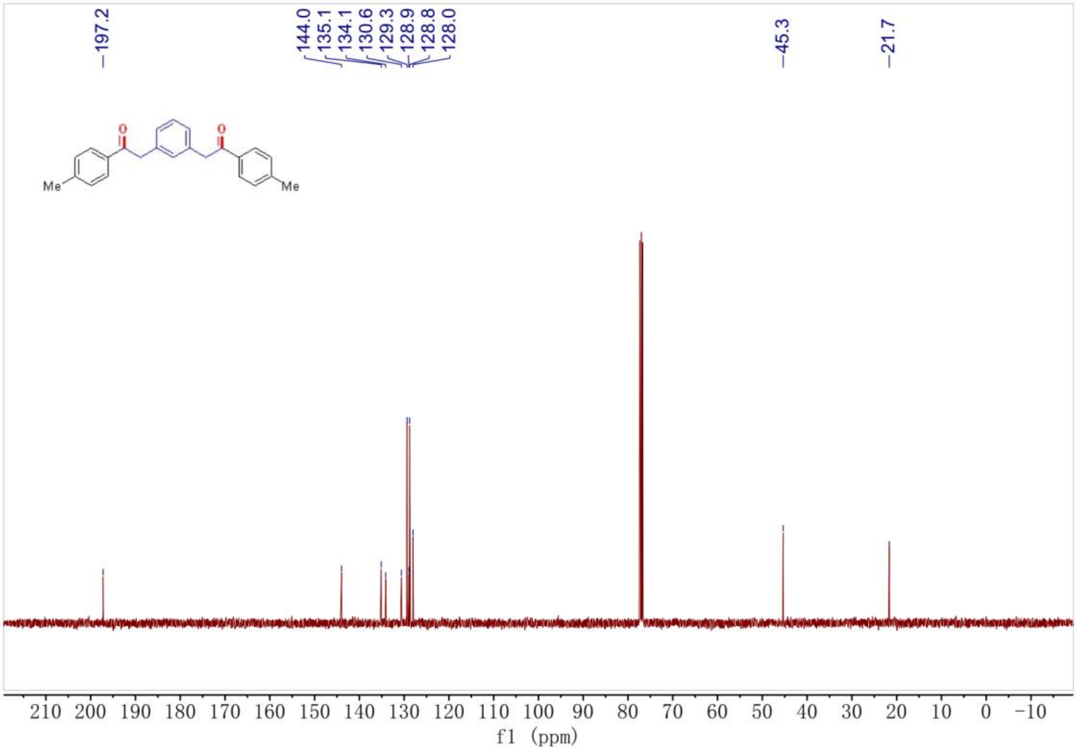

^{1}H NMR (400 MHz, $CDCl_3$) 2-14

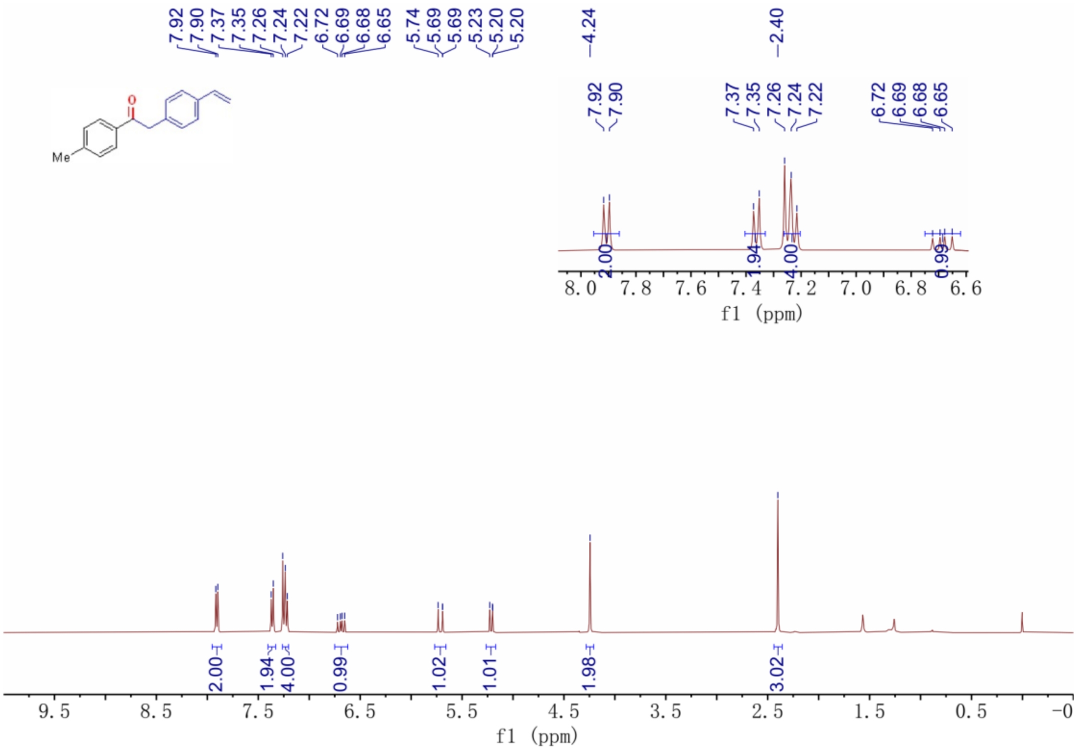

^{13}C NMR (101 MHz, $CDCl_3$) 2-14

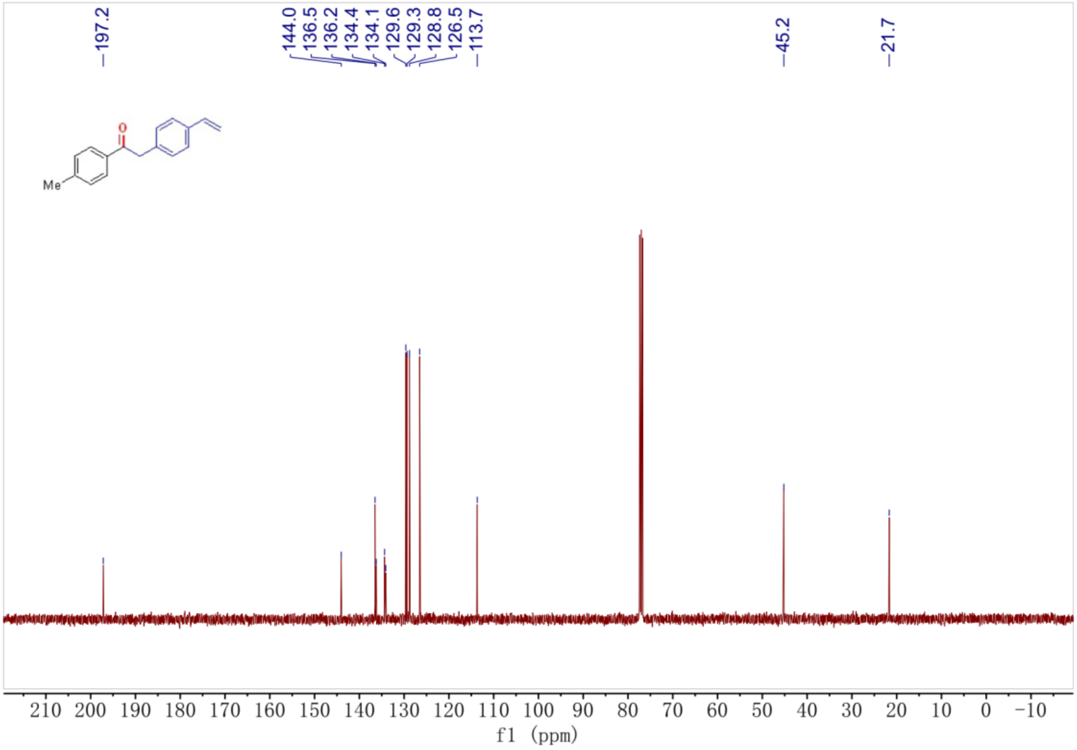

^{1}H NMR (400 MHz, $CDCl_3$) 2-15

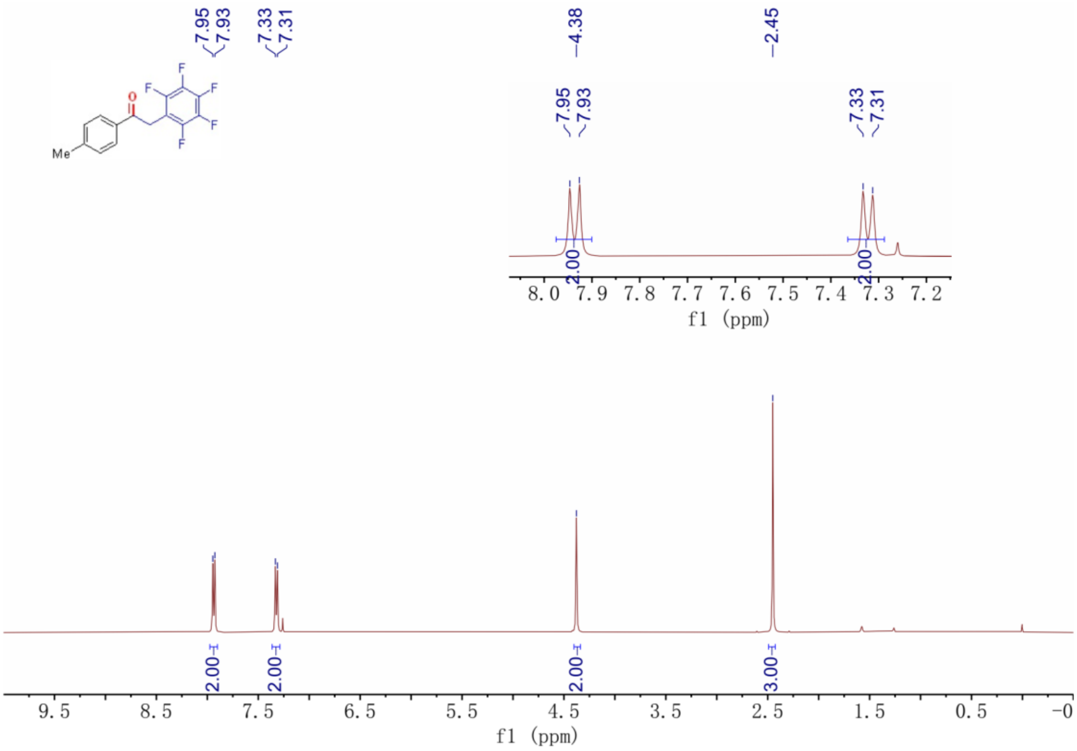

^{13}C NMR (101 MHz, $CDCl_3$) 2-15

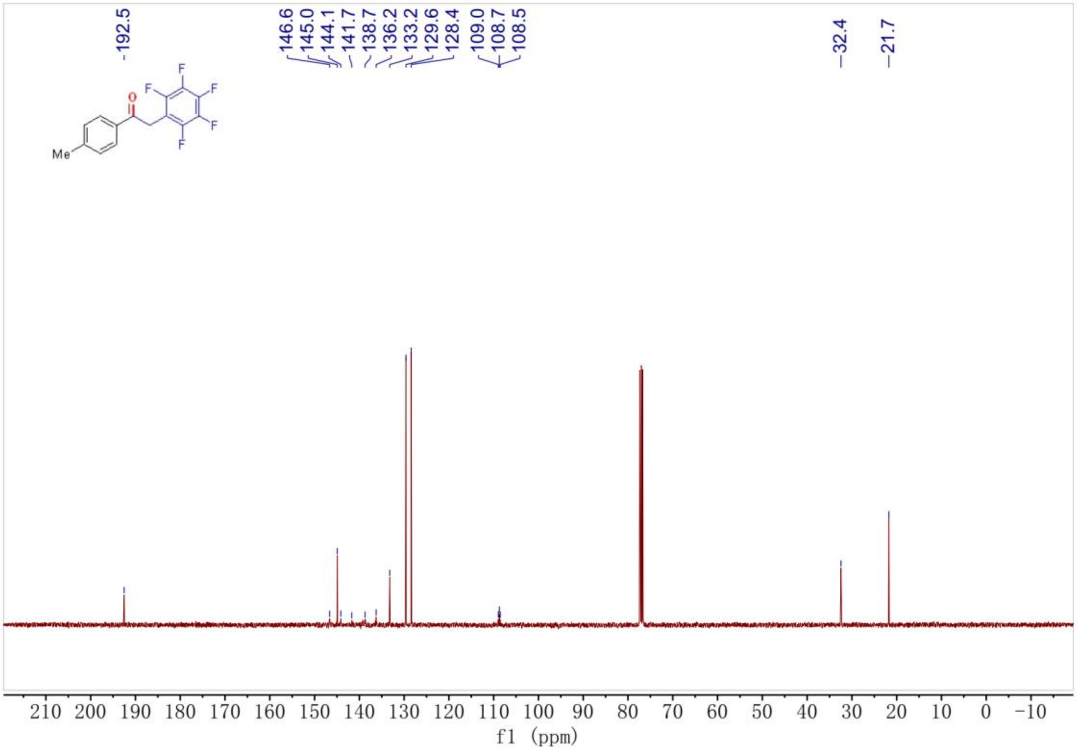

^{19}F NMR (376 MHz, $CDCl_3$) 2-15

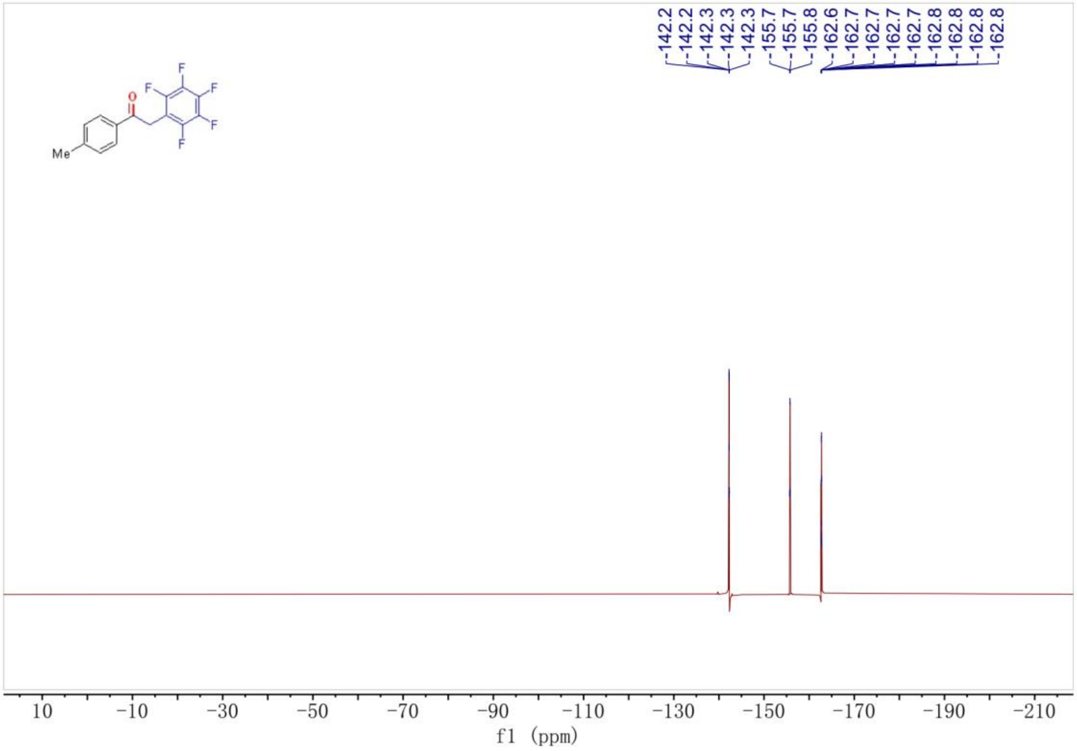

Supplement: SC-014-D3SC02402D-s001 [file SC-014-D3SC02402D-s001.pdf]
